# Trace Elements—Role in Joint Function and Impact on Joint Diseases

**DOI:** 10.3390/ijms26157493

**Published:** 2025-08-02

**Authors:** Łukasz Bryliński, Katarzyna Brylińska, Filip Woliński, Jolanta Sado, Miłosz Smyk, Olga Komar, Robert Karpiński, Marcin Prządka, Jacek Baj

**Affiliations:** 1 Department of Forensic Medicine, Medical University of Lublin, Jaczewskiego 8b, 20-090 Lublin, Poland; lukbry2@gmail.com (Ł.B.);; 2 Department of Correct, Clinical and Imaging Anatomy, Medical University of Lublin, ul. Jaczewskiego 4, 20-090 Lublin, Poland; 3 Department of Machine Design and Mechatronics, Faculty of 1 Mechanical Engineering, Lublin University of Technology, Nadbystrzycka 36, 20-618 Lublin, Poland; 4 Institute of Medical Sciences, The John Paul II Catholic University of Lublin, ul. Konstantynów 1H, 20-708 Lublin, Poland; 5 Department of Orthopedics and Movement Traumatology, Provincial Integrated Hospital, Szpitalna 45, 62-504 Konin, Poland

**Keywords:** trace elements, metallomics, joints, rheumatoid arthritis, osteoarthritis, psoriatic arthritis, anjylosing spondylitis, rheumatology

## Abstract

Proper joint function has a significant impact on people’s quality of life. Joints are the point of connection between two or more bones and consist of at least three elements: joint surfaces, the joint capsule, and the joint cavity. Joint diseases are a serious social problem. Risk factors for the development of these diseases include overweight and obesity, gender, and intestinal microbiome disorders. Another factor that is considered to influence joint diseases is trace elements. Under normal conditions, elements such as iron (Fe), copper (Cu), cobalt (Co), iodine (I), manganese (Mn), zinc (Zn), silver (Ag), cadmium (Cd), mercury (Hg), lead (Pb), nickel (Ni) selenium (Se), boron (B), and silicon (Si) are part of enzymes involved in reactions that determine the proper functioning of cells, regulate redox metabolism, and determine the maturation of cells that build joint components. However, when the normal concentration of the above-mentioned elements is disturbed and toxic elements are present, dangerous joint diseases can develop. In this article, we focus on the role of trace elements in joint function. We describe the molecular mechanisms that explain their interaction with chondrocytes, osteocytes, osteoblasts, osteoclasts, and synoviocytes, as well as their proliferation, apoptosis, and extracellular matrix synthesis. We also focus on the role of these trace elements in the pathogenesis of joint diseases: rheumatoid arthritis (RA), osteoarthritis (OA), psoriatic arthritis (PsA), ankylosing spondylitis (AS), and systemic lupus erythematosus (SLE). We describe the roles of increased or decreased concentrations of individual elements in the pathogenesis and development of joint diseases and their impact on inflammation and disease progression, referring to molecular mechanisms. We also discuss their potential application in the treatment of joint diseases.

## 1. Introduction

A joint is a point where two or more bones meet. Joints play a key role in movement. Each joint consists of at least three elements. The first element is the articular surface formed by the tips of the articular bones, the second one is the joint capsule, and the third is the articular cavity [1,2,3]. The proper functioning of joints can be compromised, often due to arthritis, which presents a significant socioeconomic challenge. Ankylosing spondylitis (AS) affects approximately 1 in 200 people [4]. However, osteoarthritis (OA) is the most prevalent form of arthritis, with its high frequency being attributed to various contributing factors [5]. Factors such as overweight and obesity can exacerbate the progression of joint diseases, while weight loss and maintaining a healthy body mass index (BMI) are crucial for symptom reduction, which highlights the important role of lifestyle [6]. Gender can also influence disease incidence, as seen in psoriatic arthritis (PsA). Female patients with PsA often report more severe pain and activity limitations compared to men, and their treatment outcomes tend to be less favourable [7]. Furthermore, the deterioration of the intestinal microbiome has been linked to the development of certain diseases [8]. Accurate diagnosis of joint diseases can be challenging due to varied symptoms and the lack of specific biomarkers, which necessitates a collaborative approach among different specialists [9]. The treatment methods for joint diseases vary depending on complications, the etiology of the disease, and the specific type of disease. Approaches include administering painkillers and anti-inflammatory drugs. Biologics are increasingly used to treat or alleviate symptoms, and disease-modifying anti-rheumatic drugs (DMARDs) can improve patients’ clinical status. In some cases, surgical intervention may be required [10].

Of the elements in the periodic table, 60 are found in the human body, but only about 20 of them are considered essential for life. Among them are trace elements, which, as elements essential for life, are present in the human body in small concentrations due to their catalytic or structural effects on larger molecules and perform specific functions [11,12]. On the other hand, trace elements can also have a toxic effect on cell function. This can occur as a result of increased oxidative stress due to the stimulation of reactive oxygen species (ROS) production, a reduction in cellular antioxidant and free radical scavenger reserves, or a reduction in the activity of enzymes involved in ROS metabolism [13]. However, the distribution of these elements in the body is not uniform. There are differences in their concentrations in tissues and body fluids, which means that these metals can accumulate in various organs and systems such as the heart, brain, kidneys, blood vessels, and immune system, thereby disrupting their functioning and contributing to the pathogenesis of diseases. These elements can also affect the musculoskeletal system [14,15].

In this review, we discuss and analyze the current knowledge on the role of trace elements in joint function and their impact on the development and progression of joint diseases, such as rheumatoid arthritis (RA), OA, PsA, AS, and systemic lupus erythematosus (SLE). This work focuses on the molecular mechanisms by which selected trace elements (including iron (Fe), copper (Cu), cobalt (Co), iodine (I), manganese (Mn), zinc (Zn), silver (Ag), cadmium (Cd), mercury (Hg), lead (Pb), nickel (Ni) selenium (Se), boron (B), and silicon (Si)) affect the cells that constitute joint tissues, including chondrocytes, osteoblasts, osteoclasts, and synoviocytes, as well as their potential application in the prevention and treatment of rheumatic diseases.

## 2. Effects of Trace Elements on Joint Function

Serving as a cofactor for numerous enzymes involved in chemical reactions that are related to the survival, growth, and metabolism of cells, Fe plays an important role in the functioning of the elements that make up the joints. Haemostasis of Fe ensures their proper functioning [16]. Overexposure to Fe can lead to cartilage damage called ferroptosis, i.e. chondrocyte death caused by a decrease in glutathione peroxidase (GPx) 4 activity with excessive production of ROS and lipid peroxides, which damages the structure and function of the whole cell [17]. By influencing osteoblasts and osteoclasts, Fe can also affect bone formation: with an increase in the Fe concentration in bone, there is an increase in the number of osteoclasts, which is associated with Fe-dependent oxidative stress. In turn, increased levels of ROS, which occur in osteoblasts exposed to excess Fe, impede the proliferation, resulting in a reduction in the population of these cells [18]. Furthermore, Fe overload can also cause inflammation of the synovial membrane: in patients with haemophilic arthritis (HA) with intra-articular bleeding, Fe overload may cause M1 macrophage polarisation and the secretion of inflammatory cytokines via p53 acetylation [19].

As for Fe, the Cu balance also plays an important role in the functioning of joint components. Cu acts as a cofactor for enzymes such as lysyl oxidases and enzymes involved in the synthesis and cross-linking of collagen and elastin, and therefore directly contributes to the integrity and strength of joint structures such as cartilage and ligaments. Moreover, Cu is a component of ceruloplasmin ferroxidase/haefestin, enzymes which are involved in the metabolism of Fe, an element whose correct concentration is also important for the proper functioning of joints [20]. It has also been shown that Cu can affect bone turnover: extracellular Cu reduces the uptake of osteoclasts, limiting their function, and adequate Cu concentrations can also improve the viability and growth of human osteoblast-like cells [21].

Due to its involvement in the production of vitamin B12, Co is an element that plays an important role in the body. However, excessive exposure to this element can harm the body, disrupting the functioning of the cardiovascular, nervous, endocrine, haematopoietic, and respiratory systems and the skin [22]. Co may also harm joints due to its impact on osteoblasts. Co ions can increase the secretion of interleukin (IL) -8 and monocyte chemotactic protein-1°(MCP-1) in primary human osteoblasts and inhibit their function, as demonstrated by observed reductions in alkaline phosphatase activity and calcium deposition [23]. Co may also cause a decrease in collagen synthesis in osteoblasts by inhibiting the expression of collagen 1 genes through disruption of the transforming growth factor β (TGF-β) signalling pathway, which activates COL1A1 synthesis, as well as disrupting collagen network formation and local changes in collagen density in the extracellular matrix [24,25]. This element also harms synovial fibroblasts by increasing the expression of genes that indicate hypoxia responses, inducing cellular stress and promoting local inflammation [26].

I is an element that is particularly important for hormonal balance: it is part of the thyroid hormones (THs) thyroxine (T4) and triiodothyronine (T3), which are key regulators of metabolism, growth, and development throughout human life [27]. The I in the THs affects the cells that build up the joint: it stimulates the maturation of chondrocytes and the progression of intra-articular ossification and is essential for linear growth. In the case of osteoblasts, I T3 increases the expression of the differentiation markers collagen I, osteocalcin, osteopontin, alkaline phosphatase, matrix metalloproteinase (MMP) 9, and MMP13 in osteoblasts and is also involved in modulating the proliferation and differentiation of these cells. Interestingly, THs, through increasing the expression of receptor activator of nuclear factor kappa-Β ligand (RANKL) and other cytokines involved in osteoclastogenesis, also indirectly stimulate osteoclasts [28].

As an indispensable cofactor of many enzymes, Mn is involved in many chemical reactions in the human body: it is a component of many metalloproteins, such as oxidoreductases, transferases, hydrolases, lyases, isomerases, and ligases [29]. Mn is also necessary for the regulation of bone development and remodelling. Dual-positive Mn ions influence osteoblasts by promoting their adhesion, viability, and proliferation via integrin activation. Furthermore, Mn is also necessary for the synthesis of proteins in bone tissue, such as type 1 collagen. In contrast, Mn has an inhibitory effect on the formation of osteoclasts. However, in the case of chondrocytes, Mn deficiency can inhibit their proliferation and induce apoptosis [30].

Zn is also an element that is essential for the body to function properly. Interestingly, it is the bones that are the main store of Zn in the human body. Zn also influences the function of the cells that make up the joint components. Zn promotes osteoblast proliferation and increases alkaline phosphatase (ALP) activity. Interestingly, these desirable effects occur in the dose range of 1–50 µM, where higher doses inhibit osteogenic activity and lower doses have no measurable effects. Very high doses such as 600 and 900 µM have cytotoxic properties. The positive effect of Zn on osteoblast development is probably related to its positive effect on Runt-related transcription factor 2 (RUNX2) expression, whose expression is essential for this process. Zn also plays a protective role by protecting osteoblasts from apoptosis. In the case of osteoclasts, although Zn is necessary for osteoclastogenesis, subnanomolar concentrations of Zn can inhibit osteoclastogenesis: inhibition of osteoclastogenesis occurs at concentrations greater than 1 µM. Its effects on this process may involve direct pathways such as inhibition of calcineurin phosphatase activity, which reduces the nuclear levels of defossified nuclear factor of activated T cells 1 (NFATc1), a key transcription factor for osteoclastogenesis, as well as indirect pathways such as inhibition of nuclear factor kappa-light-chain-enhancer of activated B cells (NF-κB) signalling and reduction of RANKL expression in other cell types. Zn can also modulate the activity of mature osteoclasts by reducing tartrate-resistant acid phosphatase (TRAP) activity, a marker of osteoclast function and bone resorption, and can induce osteoclast apoptosis. Zn also affects chondrocytes through exerting positive effects on chondrogenesis and matrix synthesis. However, its effects are biphasic, meaning that, while optimal concentrations promote beneficial effects, high levels can be cytotoxic or nullify positive results [31].

Due to its antibacterial, antifungal, and antiviral properties, Ag is used in orthopaedics in the form of Ag nanoparticles (AgNPs) in the treatment of infections. These nanoparticles may penetrate through bacterial cell walls, changing the structure of cell membranes and perhaps leading to cell death [32]. Their effect on osteoclasts and osteoblasts is not fully understood, but it has been shown that AgNPs can increase the ROS levels in osteoclasts and thus enhance their bactericidal activity, although this may also be toxic to these cells [33,34].

Overexposure to Cd can adversely affect the human body. This includes the joints: Cd can interfere with the function of the components that build the joints [35]. Cd can interfere with the differentiation of bone marrow mesenchymal stem cells (BMSCs) into osteoblasts by increasing the intracellular Ca^2+^ concentration and inhibiting receptor activator of nuclear factor-κB ligand/receptor activator of nuclear factor-κB/osteoprotegerin (RANKL/RANK/OPG) signalling. Another negative effect is the direct induction of osteoblast apoptosis through activation of the extracellular signal-regulated kinase (ERK) signalling pathway. Cd may also interfere with bone remodelling: this element mainly affects osteoclast activation, promotes bone resorption, and induces osteoblast damage and oxidative stress, which lead to the apoptosis of osteoclasts. Cd also harms chondrocytes by disrupting extracellular matrix synthesis [36,37]. Exposure to Cd also negatively affects synovial membrane cells, shortening their survival time [38].

Hg is a toxic metal, the main sources of which are contaminated seafood consumption and occupational exposure. Adverse effects of Hg exposure are commonly observed in the nervous, respiratory, and endocrine systems, as well as renal and bone marrow [39,40]. Due to Hg’s autoimmune, inflammatory, and epigenetic properties, the joints may also be affected. Hg can undergo uptake in synovial membrane cells, articular chondrocytes, and periosteal cartilage cells and disrupt their function [41].

Pb is also a toxic element that, depending on the length of exposure, can cause a variety of symptoms: increased blood pressure, slowed nerve conduction, fatigue, mood swings, drowsiness, impaired concentration, impaired fertility, reduced sexual desire, headaches, constipation, and, in severe cases, encephalopathy or death [42]. A 2021 study shows that Pb can also be deposited in joint components [43]. There, it can affect the cells that build up the joints. It has been shown that Pb can have a toxic effect on osteoblasts and lead to their apoptosis. This process may occur through inhibition of wingless-related integration site (Wnt)/β-catenin signalling, a critical anabolic pathway for osteoblastic bone formation [44]. The negative effects of Pb may also affect chondrocytes. This trace element can induce apoptotic death in mesenchymal stem cells (MSCs) from the bone marrow, which are the precursors of chondrocytes, by increasing the expression of the proteins p53, Bcl-2-associated X protein (Bax), and caspases 3 and 9. Also, in these cells, it was observed to significantly decrease the expression of the COL2A1 gene, which is essential for cartilage [45].

The role of Ni in the human body is not fully understood. Although no Ni-containing enzymes are known to exist in higher organisms, experiments on animals with a Ni-deficient diet have shown certain effects, such as impaired Fe absorption, reduced concentrations of other metals (Cu, Zn) in the liver, and reduced activity of enzymes involved in carbohydrate and amino acid metabolism [46]. As for the effect of Ni on joints, it may have a toxic effect on osteocytes, causing their apoptosis [47].

As a component of selenoproteins, Se has many important functions in the body. It takes part in antioxidant defence and immunomodulation and regulates cell cycle progression and proliferation. It is also important for the proper functioning of joints [48,49]. As an essential component of glutathione peroxidase (GSH-Px), Se participates in reducing oxidative stress in cells [50]. Expression of selenoproteins by bone cells may contribute to protection against oxidative stress in the bone microenvironment, and this prevents osteoporosis. Selenoproteins also have an impact on bone formation: thioredoxin reductase 1 (TR1) is a selenoprotein that is expressed early in the osteoblast differentiation signalling cascade and is involved in the regulation of this process. In contrast, GPx1, as an antioxidant enzyme in osteoclasts, may inhibit osteoclastogenesis. Furthermore, it is suspected that Se, at least in high doses, may prevent bone resorption by inactivating osteoclasts through its mechanism of regulating osteoblast–osteoclast interactions [51,52]. Se also plays an important role in chondrocytes. Selenoproteins such as iodothyronine deiodinase-2 (DIO2), GPx1, and TR1 protect chondrocytes from damage, apoptosis, and destruction of the cartilage matrix by oxidative stress [53,54]. 

B is an element that has a beneficial effect on bones and joints. B is involved in bone metabolism, influencing the activity of osteoblasts and osteoclasts. It is suggested that a deficiency of B leads to a reduction in osteoblast activity and promotes the formation of osteoclasts. It plays a role in maintaining the balance of calcium and other minerals that are essential for healthy bone structure. B also supports the activation of vitamin D, which is essential for the proper functioning of the skeletal and joint systems [55,56,57]. In the context of joints, B has a beneficial effect on chondrocytes. Studies indicate that B can increase chondrocyte proliferation and support the formation of the cartilage matrix. These effects are crucial for cartilage health and its ability to regenerate itself [58]. In addition, B has anti-inflammatory and antioxidant properties that can protect joint cells from damage caused by oxidative stress and inflammation [58,59,60].

Si has a significant impact on bone and joint cells. It has a dual effect on bone metabolism by simultaneously stimulating osteoblasts and inhibiting osteoclasts [61]. Bioactive silica nanoparticles and soluble Si directly stimulate osteoblasts to form bone and mineralise, increasing the alkaline phosphatase activity and proliferation while inhibiting the osteoclast activity and formation, which contributes to a reduction in bone resorption and an increase in bone mineral density [62,63,64,65]. Si also plays a role in the formation of articular cartilage and connective tissue. In vivo studies have shown that its presence is physiologically required for the proper development of these tissues, and Si deficiency is associated with abnormalities in the articular cartilage, such as reductions in the cartilage volume and reductions in matrix components, including collagen and glycosaminoglycans. Si supplementation, on the other hand, can increase the amount of joint cartilage and its components, such as hexosamines. It appears that Si is essential for the formation of the organic matrix of bone and cartilage. However, the formation of this matrix is more susceptible to Si deficiency than it is to mineralisation [66,67].

**Figure 1:**
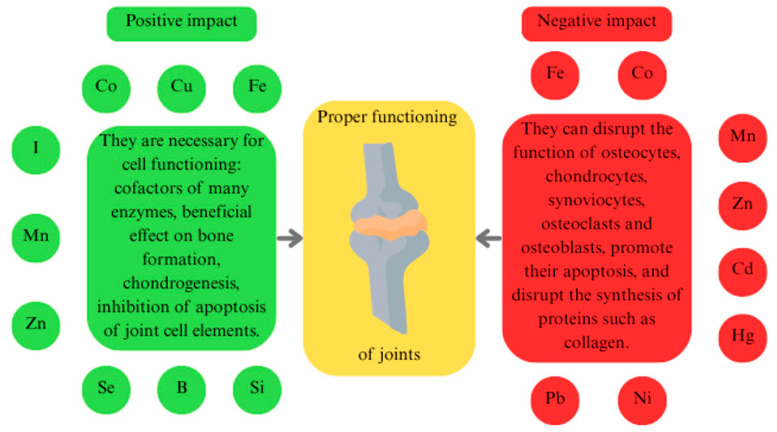
The role of trace elements in the functioning of joint components. Abbrevitions: Ag—silver; BMSCs—bone marrow mesenchymal stem cells; B—boron; Cd—cadmium; Co—cobalt; Cu—copper; Fe—iron; GSH-Px—glutathione peroxidise; Hg—mercury; I—iodine; Mn—manganese; MSCs—mesenchymal stem cells; Ni—nickel; Pb—lead; Se—selenium; Si—silicon; TH—thyroid hormones; Zn—zinc.

The role of trace elements in the functioning of joint components is summarised in Figure 1.

## 3. Beneficial and Detrimental Interactions Between Trace Elements for the Human Body

**Figure 2:**
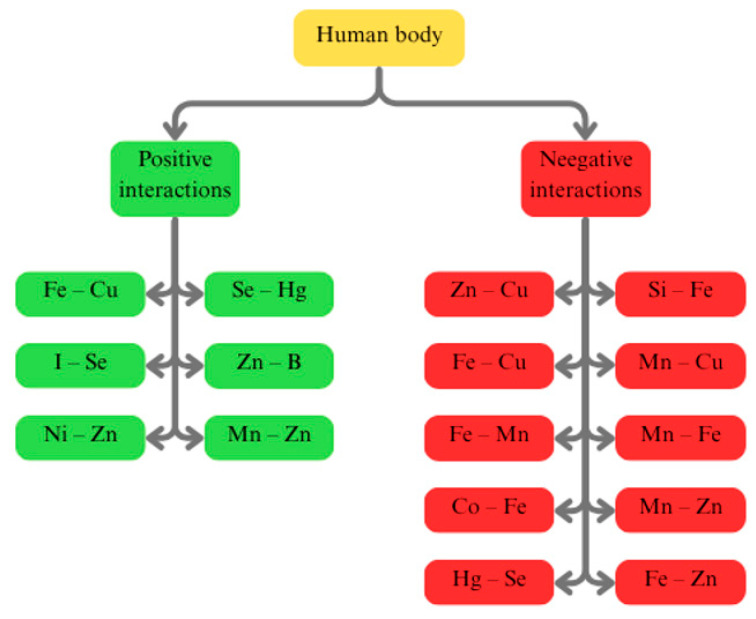
Beneficial and detrimental interactions between trace elements in the human body. Abbreviations: B—boron; Co—cobalt; Cu—copper; Fe—iron; Hg—mercury; I—iodine; Mn—manganese; Ni—nickel; Se—selenium; Si—silicon; Zn—zinc.

An important aspect of trace elements in the human body is their interaction. These interactions are complex processes that have significant consequences for human health. They can be either synergistic or antagonistic (Figure 2). A classic example of antagonism is the competition between Zn and Cu for intestinal absorption, where excessive Zn intake can lead to secondary Cu deficiency, which affects immune and cardiovascular functions [68]. Similarly, high doses of Fe can cause Cu deficiency, even though Cu positively affects Fe homeostasis through ceruloplasmin [69]. Fe and Zn compete for absorption in the gastrointestinal tract, which results from competition for the same transport pathways, such as the divalent metal transporter 1 (DMT1), zinc-regulated transporter, and iron-regulated transporter-like protein (Zip14) pathways [70,71]. High doses of Fe can significantly reduce Zn absorption, especially when they are taken simultaneously in the form of supplements on an empty stomach, although this effect is less pronounced when they are taken with food [72,73,74]. Furthermore, long-term or high Zn supplementation may inhibit Fe absorption, leading to a decrease in the serum Fe concentration and even causing Fe deficiency anaemia [75,76]. As well, Fe and Mn exhibit synergistic interactions, especially in the brain, where both a deficiency and excess of Fe can increase the Mn concentration, exacerbating neurotoxicity [77]. Another important interaction is that between I and Se, where the effective use of I in the synthesis of thyroid hormones depends on selenoproteins such as DIOs, and Se deficiency can impair thyroid function and immunity [78,79]. Although essential for erythropoiesis as a component of vitamin B12, it can compete with Fe for binding sites and, in excess, contribute to oxidative stress and carcinogenesis [80,81]. Ni directly interacts with intracellular Zn, affecting its homeostasis by increasing its labile pool in respiratory epithelial cells. This released Zn is crucial for the activation of the metal transcription factor-1 (MTF-1) and its translocation to the cell nucleus. As a result, this interaction between Ni and Zn leads to the induction of metallothionein (MT2A) expression; MT2A is a protein with a protective effect [82]. Among the toxic elements, Hg exhibits a complex interaction with Se, where Hg binds selenoproteins, which leads to functional Se deficiency, although Se can also reduce Hg toxicity [83]. Ag accumulates in tissues in the form of silver selenide, which is the result of Se’s strong tendency to precipitate it and promote the retention of Ag in the body. However, the resulting silver selenides are considered inert and do not cause irreversible toxic changes [84]. Zn and B form a stable, non-toxic complex that is well absorbed and metabolised in the body. This interaction may reduce the potential toxicity of B and leads to increased expression of alpha2-macroglobulin, which suggests health benefits [85]. Silica can bind mitochondrial Fe, which leads to oxidative stress, disruption of Fe homeostasis in the cell, and inflammation in the body [86]. One study found that exposure to high doses of Mn that are administered orally significantly affects the levels of other essential metals, such as Cu, Fe, and Zn, in rat tissues. Among the metals that were studied, the levels of Cu were the most significantly increased. The key tissues in which these changes were observed were the liver and the frontal cortex of the brain. It was found that intragastric Mn administration had a greater effect on the metal levels in tissues than administration in the diet or drinking water. The results suggest that excess Mn in the liver may interfere with Cu excretion, indicating a complex relationship between these elements. Altered metal levels, especially in the brain, may complicate the neurological effects of Mn toxicity, as elevated concentrations of Cu, Zn, and Fe are also associated with a risk of neurodegenerative diseases [87]. Mn and Zn also play a cooperative role in preventing carcinogenesis by influencing mitochondrial metabolism, transcription regulation, and chromatin dynamics, as well as the immune response [88]. Many other interactions between the elements we describe herein remain less well documented in the context of direct physiological interactions in humans. Understanding these complex interdependencies is crucial for safe and effective supplementation, which requires consideration of the entire mineral profile of the body.

## 4. The Role of Trace Elements in Joint Diseases

In this chapter, we discuss the role of the trace elements Fe, Cu, Co, I, Mn, Zn, Ag, Cd, Hg, Pb, Ni Se, B, and Si in the pathogenesis of the joint diseases RA, OA, PsA, AS, and SLE, as well as their effects on tissue levels and their use in treatment.

### 4.1. The Role of Trace Elements in Rheumatoid Arthritis

RA is one of the most prevalent autoimmune arthropathies and is estimated to affect 0.5–1% of the population, with women being two to three times more affected than men [89,90]. The risk factors for RA include genetics (especially inheritance of HLA-DRB1*01 and HLA-DRB1*04), female sex, obesity, smoking, and silica exposure [91]. Initially, RA manifests as pain, tenderness, and morning stiffness that mainly affects the small joints of the hands and feet, often symmetrically. However, as the disease progresses, it begins to affect larger joints, e.g., the knee joint, and other systems and organs, such as the kidneys, eyes, respiratory system, and cardiovascular system, which may predispose the afflicted individual to premature death [92,93].

The exact etiology is not fully understood, but it is suggested that RA may be caused by synovial macrophages, which secrete proinflammatory factors such as tumor necrosis factor-alpha (TNF-α), IL-1, and IL-6, which induce inflammation and damage to the cartilage and bones [94]. Additionally, the accumulation of macrophages, plasma cells, and fibroblast-like synovial cells (FLSs) in the sublining of the synovial membrane results in the formation of an inflamed "pannus" tissue at the bone–cartilage interface; the cells present in this pannus can produce rheumatoid factor (RF) autoantibodies and autoantibodies against citrullinated peptides (APCAs), which lead to further joint destruction [95,96].

#### 4.1.1. The Role of Fe in Rheumatoid Arthritis

Fe metabolism disorders are a frequently reported problem among people with RA (Table 1). Research results indicate a reduced Fe concentration in the serum and synovial fluid in patients with RA [97,98,99]. Studies by Khalaf et al. [100] have shown that Fe and red blood cell parameters are lower in people with RA than in the healthy population. This may be due to increased levels of hepcidin in the body due to chronic inflammation, which inhibits Fe absorption in the intestine, blocks the release of Fe from the macrophages of the reticuloendothelial system (RES), and, therefore, may contribute to the high incidence of anemia of chronic disease (ACD) or Fe deficiency anemia (IDA) in people with RA [100,101]. The correlation between the ongoing inflammation in the body and its markers (CRP, IL-6, prohepcidine) and Fe levels was confirmed by the study by Stefanova et al. [102], which was conducted on a group of 114 patients with RA, whose serum Fe levels were significantly lower than that of the control group. Additionally, the comparison between the subgroup of patients with RA, when divided based on disease activity, showed a reduced Fe concentration regardless of disease activity. The importance of prohepcidine and soluble transferrin receptor (sTfR) parameters as a reliable prognostic indicator for monitoring Fe metabolism disorders in patients with RA was also demonstrated [102]. Synovial membrane biopsies were taken from patients with RA and OA and then compared with each other for the presence of Fe deposits. In the patients with RA, Fe deposits were detected that were almost twice as high as those in the patients with OA, which may indicate a relationship between their presence and the pathophysiology of chronic inflammation in people with RA. Additionally, the hemoglobin values and the number of deposits were correlated in the patients studied, and an inverse correlation between them was obtained [103]. Increased Fe deposition in the synovial membrane may induce macrophage ferroptosis, which is likely to be involved in the development of RA; currently, research is underway on the importance of Fe the pathomechanism of this disease, as well as on its therapeutic use in the treatment of RA [104,105]. However, despite the significant predominance of studies suggesting a decrease in the serum Fe concentration in patients with RA, there are also studies that report no significant differences in serum Fe concentrations between patients with RA and healthy individuals [106]. This shows that the current state of knowledge on this subject is insufficient and that further research is required to understand the nature of the disease better and to create appropriate tools for its monitoring and treatment.

#### 4.1.2. The Role of Cu in Rheumatoid Arthritis

Numerous studies have been conducted to demonstrate the association between the Cu levels and the occurrence of RA in the studied patients, with most of them indicating increased serum Cu levels [98,107,108]. Strecker et al.’s analyses [109] of blood and hair samples showed that the concentration of Cu in the studied samples was statistically higher in patients with RA in the control group, while the concentration of Cu in erythrocytes in these patients was reduced [109]. This may be related to chronic inflammation, in which there is increased synthesis of IL-1, IL-6, and TNF-α, which stimulates hepatocytes to synthesise ceruloplasmin, resulting in an increased serum Cu concentration [107,110]. In contrast to the increased serum Cu concentration, a frequently reported phenomenon, the decreased Cu concentration in the erythrocytes of patients with RA, is not often studied and raises some contradictions in the literature. Some researchers do not demonstrate this effect, while those who report it suggest the influence of abnormal superoxide dismutase activity, which leads to reduced Cu concentration in the cells [109]. Additionally, Ullah et al. [111] suggested that an increased dietary Cu intake due to the use of Cu cooking utensils in the studied population may also influence the serum Cu concentration in the studied patients [111]. Research is ongoing on the relationship between cuproptosis and the development of RA, as a correlation has been shown between the overexpression of cuproptosis-related genes (CRGs) in chondrocytes, which results in chondrocyte death and cartilage damage and consequently may contribute to disease progression. Therefore, research is ongoing on the use of drugs that affect cuproptosis to limit RA progression [112].

#### 4.1.3. The Role of Co in Rheumatoid Arthritis

Heavy metals, including Co, are considered potential contributors to the induction of RA. These metals can generate ROS, which leads to oxidative tissue damage and thus contributes to the development and progression of RA [113]. Unfortunately, compared to other heavy metals, there is a lack of studies focusing on the effect of Co on RA. However, Co, specifically Co (II) nanocomplexes, has been investigated as a therapeutic option for RA. These nanocomplexes have been shown to have anti-inflammatory properties and may be potentially useful in the treatment of RA. They are also stable and environmentally friendly. However, it should be emphasised that these results were obtained in an animal model of RA, so further research is needed to confirm the efficacy and safety of this approach in humans [114].

#### 4.1.4. The Role of Mn in Rheumatoid Arthritis

Another important element in the context of RA is Mn. One study showed elevated Mn concentrations in granulocytes isolated from patients with RA. However, the erythrocytes and platelets in these patients usually contained normal amounts of Mn. The accumulation of Mn in granulocytes appears to be intrinsically linked to the intensity of the inflammatory process, as evidenced by positive correlations with inflammatory markers such as the erythrocyte sedimentation rate (ESR) or serum haptoglobin [115]. However, another study showed increased Mn concentrations in red blood cells in people with RA [116]. The Mn levels in synovial fluid were also examined and found to be lower in patients with RA compared to healthy individuals. In plasma, the Mn levels were similar between patients with RA and the control group [117]. In another study of element levels, including Mn, the control group had higher levels than the group of people with arthritis (including RA), but there was no significant difference between the two groups [118]. Another study confirms that there is no significant association between RA and serum Mn levels [119]. However, a study by Khadim et al. [99] that was conducted on 120 patients with RA and 60 healthy women provided different results. Lower Mn concentrations were observed in women with RA compared to the control group [99]. However, testing the Mn levels in 20 patients with RA and 20 healthy individuals indicated higher average Mn concentrations in the patients with RA than in the control group [120]. The Mn concentrations in scalp hair samples were also examined, and patients with RA were found to have reduced Mn levels compared to healthy control groups. Interestingly, it was observed that the Mn concentrations did not differ significantly between scalp samples from smokers and samples from non-smokers [121]. Mn, as a key component of Mn superoxide dismutase (MnSOD), may play an important role in antioxidant defence in RA. A 1984 study shows that a decrease in the MnSOD activity in granulocytes may be characteristic of RA and contribute to tissue damage through impaired regulation of superoxide radicals [122]. The therapeutic potential of MnSOD was investigated in rats with RA-like arthritis. After using MnSOD in treatment, a significant reduction in paw oedema was observed [123]. In another study on a rat model of arthritis, the therapeutic effect of the compound M40403 was tested. This synthetic Mn-containing SOD mimetic was found to be effective in alleviating joint disease in an animal model of RA. A reduction in swelling and a decrease in damage to the joint cartilage and bone were observed. Histopathologically, a reduction in inflammatory cell infiltrates was observed. M40403 also reduced the level of the inflammatory marker nitrotyrosine. This confirms its antioxidant and anti-inflammatory effects and indicates the therapeutic potential of Mn compounds in the treatment of RA [124]. Another Mn-containing SOD mimetic, MnIIMe2DO2A, showed a significant effect in reducing joint pain in rats with induced arthritis. This compound reduced the mechanical and thermal hypersensitivity in the joint area. Its analgesic effect was associated with a reduction in inflammation and oxidative stress in joint tissues. This study also indicates the potential therapeutic effect of Mn compounds and the possible alleviation of RA symptoms [125].

The potential use of Mn dioxide (MnO_2_) nanoparticles in the treatment of RA is also being investigated. These nanoparticles utilise acidic pH conditions, in which MnO_2_ can effectively decompose and consume abnormally elevated hydrogen peroxide, a key ROS, while simultaneously producing oxygen. This dual action may affect RA by reducing hypoxia and removing excess ROS. In addition to these capabilities, MnO_2_ nanoparticles also contribute to the regulation of the inflammatory microenvironment by promoting the phenotypic repolarisation of macrophages from the pro-inflammatory M1 phenotype to the anti-inflammatory M2 phenotype. An important advantage of MnO_2_ nanoparticles is their biodegradability. They decompose into harmless, water-soluble Mn^2+^ ions, which are rapidly excreted by the kidneys. The use of MnO_2_ nanoparticles in therapy offers the possibility of directly influencing many interrelated pathogenic features of RA, such as excessive ROS, hypoxia, and M1 macrophage dominance [126]. The UMnEH nanocomposite exhibits very similar activity, including antioxidant properties and oxygen generation, in microwave-assisted RA therapy. It was created using Mn tetroxide (Mn_3_O_4_) nanoparticles [127]. In addition to their properties, Mn oxide nanoparticles can serve as effective drug carriers. In study from 2024, methotrexate (MTX) was loaded into nanoparticles, which were then modified with bovine serum albumin and incorporated into hyaluronic acid-based soluble microneedles for transdermal administration. This innovative approach significantly improved the efficiency of drug delivery, reduced inflammation in RA, and effectively modulated macrophage polarisation towards an anti-inflammatory phenotype [128]. The properties of MnO_2_ were also used in the creation of nanoplatforms for MTX delivery in RA. In the studied model, inflammation was alleviated and joint damage was reduced [129]. A nanocarrier was also designed in which Mn is not only a structural element but, more importantly, an active ingredient that transforms an enzyme-like complex (manganoporphyrin). This synthesised manganoporphyrin exhibits activity mimicks of superoxide dismutase and catalase. Nanomedicine is currently developing rapidly, and the use of Mn and its anti-inflammatory and immunomodulatory effects offers new possibilities in the treatment of RA [130].

#### 4.1.5. The Role of Zn in Rheumatoid Arthritis

Another element tested in the serum of patients with RA is Zn. One such study found no difference between the Zn concentrations in people with RA and those in a healthy control group [119]. However, most available studies report reduced Zn concentrations in people with RA [99,107,108,120,131,132,133]. In one study, serum Zn levels were measured using colourimetry in patients with RA and healthy individuals. Here, too, significantly lower serum Zn concentrations were found in the patients with RA compared to the control group. In addition, a negative correlation was also found between the serum Zn levels and the disease activity (according to the DAS-28 scale) and oxidative DNA damage [134]. In female patients with RA, a negative correlation was also found between the serum Zn levels, ESR, acute phase proteins, and pro-inflammatory cytokine levels, such as those of IL-1β and TNF-α, which may also suggest that lower Zn levels are associated with higher disease activity [135]. However, another study found no correlation between Zn levels and disease activity, as determined by DAS28 in people with RA, but did find a negative correlation between serum Zn levels and disease duration [136]. Low Zn levels were also observed in hair samples from patients with RA [121,136,137]. This Zn deficiency may result from impaired absorption [138]. A study has shown no causal relationship at the genetic level between Zn and the development of RA. This suggests that any correlations between this element and RA are due to factors other than genetics [139]. The effect of Zn on RA has been the subject of numerous studies, one of which proved that an increased supply of Zn in the diet of patients with RA was associated with a reduced risk of developing osteopenia or osteoporosis. Therefore, it seems potentially beneficial to include supplementation of this element in the treatment of patients with RA [140]. This is particularly true as it has been shown that patients with RA had a statistically lower dietary intake of Zn compared to a healthy control group. Low Zn levels may contribute to increased oxidative stress, as Zn is an essential cofactor for many antioxidant enzymes. Compared to the control group, lower superoxide dismutase (SOD) and GPx activities were observed in patients with RA. Reduced antioxidant activity may exacerbate the inflammatory processes and tissue damage characteristic of RA [132]. Increased oxidative stress in the cells of patients with RA is also confirmed by a study which found significantly higher levels of 8-hydroxy-2-deoxyguanosine (8-OHdG) in the serum of patients with RA compared to the control group. Importantly, a correlation was found between low serum Zn levels and high 8-OHdG levels [134]. This Zn deficiency in patients with RA may have other consequences. One study suggests that deficiency and reduced expression of the Zn finger protein A20 (a protein of which Zn is an integral component) in RA synovial tissues may lead to increased pyroptosis in RA synoviocyte-like fibroblasts through dysregulation of the NLR family pyrin domain containing 3 (NLRP3)/caspase-1 pathway [141]. Therefore, attempts to use Zn in the treatment of RA seem reasonable. In a study by Hasan et al., rats with induced arthritis were administered Zn aspartate and Zn citrate for 4 weeks. After the treatment, a reduction in paw swelling and a decrease in RF, ACPA, and CRP levels were observed compared to the control group. The use of Zn aspartate and Zn citrate also resulted in a reduction in joint erosion and osteophyte formation. Importantly, no adverse effects were observed [142]. However, in 1976, a study was conducted in which patients with RA were given oral Zn sulphate. Twenty-four patients with chronic, resistant RA participated in the study. The patients treated with Zn showed better improvement compared to the control group (receiving a placebo) in terms of joint swelling and pain, morning stiffness, and the patient’s overall assessment of the disease’s activity. This therapy was well tolerated by patients [143]. However, two studies conducted 6 years later do not confirm the therapeutic effect of orally administered Zn sulphate on RA [144,145]. Therefore, the validity of using Zn sulphate in RA is not entirely clear. As with the previous element, the development of nanomedicine has also led to the development of Zn products that can be used to treat RA. Zn nanoparticles in the form of Zn ferrite nanoparticles (ZF-NPs) that are targeted at fibroblast activating protein (FAP) induce apoptosis of synoviocyte-like fibroblasts in RA, i.e., cells that play a key role in joint destruction in this disease. In an animal model of adjuvant-induced arthritis, these FAP-targeted ZF-NPs led to the alleviation of symptoms and disease progression by suppressing synovial inflammation, inhibiting angiogenesis in synovial tissue, protecting articular cartilage from damage, and reducing the infiltration of M1 (pro-inflammatory) macrophages into the synovial membrane [146]. The use of Zn oxide nanoparticles (ZnO NPs) also showed beneficial effects in a rat model of RA. ZnO NPs were administered orally for 14 days. The results of the study showed that ZnO NPs significantly reduced the increased production of pro-inflammatory mediators in the RA model. This included IL-1β and TNF-α, among others. There was also a reduction in the ACPA levels and total leukocyte count [147]. A good therapeutic effect was also demonstrated for chitosan nanoparticles loaded with Zn gluconate (ZG-Chit NPs). In rats with arthritis that were treated with ZG-Chit NPs, a reduction in arthritis was observed through a reduction in joint swelling and erythema [148].

#### 4.1.6. The Role of Ag in Rheumatoid Arthritis

In studies, Ag, or more precisely its nanoparticles, appears in the context of its use in the treatment of RA. One study evaluated the therapeutic potential of AgNPs, biosynthesised using an extract from the Commiphora mukul plant (G-AgNPs), in the treatment of RA. The efficacy of G-AgNPs was evaluated in an adjuvant-induced arthritis mouse model and they showed strong anti-inflammatory activity, manifested by a reduction in paw oedema and a decrease in inflammatory cell infiltration in ankle joint tissues. G-AgNPs also increased the activity of antioxidant enzymes (SOD and CAT), which suggests their ability to combat oxidative stress, an important factor in the pathogenesis of RA [149]. A similar therapeutic effect, a reduction in ROS and a shift of macrophage polarisation from the M1 to M2 phenotype, was observed with Ag-modified cerium oxide nanoparticles loaded with celastrol (Ag-CeNP@Cel) [150]. Another study focused on fructose-coated Ångstrom-scale Ag particles (F-AgÅPs) and their efficacy was tested in two recognised mouse models of RA: one resembling the classic model of autoimmune arthritis and the other corresponding to acute antibody-induced arthritis. The results showed that F-AgÅPs alleviated arthritis in both models by reducing paw swelling and synovial inflammation in the joints, and also effectively inhibited osteoclast formation. As a result, they reduced bone and cartilage damage in the joints. They also reduced the levels of pro-inflammatory cytokines such as TNF-α, IL-1β, and IL-6. This multidirectional effect suggests that Ag molecules may represent a promising new therapeutic strategy in RA [151].

Proteus mirabilis is considered one of the potential factors that causes or exacerbates RA, and the use of Ag as an antibacterial agent has been suggested. The in vitro efficacy of 10 different Ag preparations—both ionic Ag [Ag(I)] and nanoparticle Ag (NPS) [Ag(0)]—against Proteus strains was evaluated, and it was found that Ag (both in ionic and nanoparticle form) is very effective in inhibiting the growth of and killing Proteus bacteria. This means that Ag may have an indirect therapeutic effect on RA by combating these potentially pathogenic bacteria [152].

#### 4.1.7. The Role of Cd in Rheumatoid Arthritis

Cd also plays an important role in the pathogenesis and development of RA. A study conducted between 1999 and 2018 showed that high concentrations of Cd in the urine and blood are positively correlated with the risk of developing RA [153]. A study by Joo SH et al. also showed a correlation between a higher incidence of RA in women and elevated serum Cd levels [119]. Cd is also deposited in the hair of people with RA, and chronic exposure to Cd, which, due to its ease of storage and availability, can also be used to study and monitor the exposure to this element [137]. People who are occupationally exposed to Cd and who smoke cigarettes, due to their Cd-containing smoke, are more likely to develop RA. It has been shown that inhalation exposure to Cd may predispose antigen-presenting cells to producing citrullinated peptides and proteins by increasing their calcium concentration and activating the enzyme peptidyl arginine deiminase, which replaces the amino acid arginine with citrulline in proteins. This process may lead to the production of ACPA and RF, which play an important role in the pathogenesis of RA. Furthermore, inhalation exposure to Cd is strongly associated with the formation of nodules (granulomas) in the lungs of patients with RA. Cd may also have pro-inflammatory effects due to its promotion of ROS production, which may also contribute to the development of RA [154,155,156]. In addition, Cd may raise the risk of death from RA. Elevated blood Cd levels may be a risk factor for increased mortality risk in patients with RA, which may be linked to the induction of severe systemic inflammation, according to a study looking at the relationship between blood Cd levels and all-cause mortality in adult patients with RA [157]. Despite its influence on the pathogenesis and development of RA, Cd also demonstrates potential usefulness in the treatment of this disease. A study conducted in rats showed that intra-articular injections of Cd can inhibit joint destruction by inhibiting the growth of and inducing apoptosis of hypertrophied inflammatory synoviocytes and inflammatory effector cells. However, further studies are needed to assess the systemic and local effects [158].

#### 4.1.8. The Role of Hg in Rheumatoid Arthritis

Hg, as a toxic metal, plays a role in RA. A 2015 study showed that people with RA had higher levels of Hg in their blood and hair than the control group. These levels were higher in cigarette smokers, which indicates cigarette smoke as a source of this element [131]. Hg may have a toxic effect on synovial membrane cells in people with RA. A study conducted on these cells taken from donors with RA showed that Hg disrupts the DNA function of these cells and inhibits collagen synthesis [159]. Exposure to Hg can also cause autoimmune dysfunction and systemic inflammation. Studies conducted on a cohort of miners occupationally exposed to Hg showed higher concentrations of the pro-inflammatory cytokines IL-1β, TNF-α and IFN-γ, as well as higher titres of autoantibodies directed against antigens located in the nucleus (antinuclear antibodies, or ANA). Oxidative imbalance and pro-inflammatory effects can lead to autoimmune diseases such as RA [160,161]. Hg may also be used in the treatment of RA. In a study on a mouse model of RA, it was shown that a hydrogel with Hg sulphide nanoparticles had an antagonistic effect on acute inflammation and, in a mouse model of RA, inhibited swelling of the finger joints and synovial membrane hypertrophy. However, further research into its use is required, as it may exhibit potential nephro- and hepatotoxicity [162].

#### 4.1.9. The Role of Pb in Rheumatoid Arthritis

Elevated levels of Pb in the blood and urine have also been found in people with RA. Due to causing oxidative damage and its inflammatory and immunological properties, Pb may contribute to the development of this disease [99,153,163]. Similarly to Cd, Pb is also deposited in the hair of people with RA, and its concentration can help monitor exposure to Pb. Interestingly, the levels of this element were higher in the group of patients who smoked compared to the group of patients who did not smoke [137]. Pb also disrupts the function of syncytiocytes by inhibiting collagen synthesis and disrupting the DNA function of these cells [159].

#### 4.1.10. The Role of Ni in Rheumatoid Arthritis

A study conducted on patients with RA showed that the Ni levels in their sweat, serum, and urine were higher compared to those in the control group [164]. A second study from 2014 also showed that, in the RA group, the average Ni levels in scalp hair and blood samples were higher compared to those in the healthy control group [165]. In contrast, a study from 1962 showed that the levels in people with RA were comparable to those in the control group [166]. However, looking at the reported results, it seems that increased Ni levels may contribute to the development of RA by disrupting antioxidant function, depleting glutathione, increasing ROS production, and inducing inflammation [99].

#### 4.1.11. The Role of Se in Rheumatoid Arthritis

Due to its antioxidant properties, low Se levels in the body appear to be positively correlated with the risk of developing RA. A study conducted on a Finnish cohort of 18,709 adult men and women showed that low serum Se levels increased the risk of developing RA [167]. Lower serum Se levels were also found in the group of patients with RA compared to the healthy control group in several other studies [108,118,168]. The Se level was also measured in the synovial fluid of patients with RA. As in blood plasma, its concentration was lower compared to that in the healthy control group [97]. Se is responsible for the activity of the GSH-Px enzyme, which is involved in the regulation of oxidative stress. A study conducted on six patients with RA showed that the GSH-Px activity in red blood cells and serum was reduced compared to that in a healthy control group. Interestingly, after Se supplementation, the GSH-Px activity in the study group increased [169]. In addition to its effect on redox balance, Se may also inhibit osteoclastogenesis by suppressing RANKL expression on CD4+ T lymphocytes, reducing the pro-inflammatory cytokines CRP and IL-6, and inhibiting Th17 cells. IL-6 and Th17 cells are key mediators of inflammation in RA. The above-mentioned mechanisms lead to a reduction in bone destruction and alleviation of inflammation, which delays the onset of the disease and alleviates joint pathologies [170].

Se supplementation may play a supportive role in the treatment of RA. A 2023 meta-analysis showed that Se supplementation improved the disease activity score 28 (DAS28) in people treated with DMARDs [171]. Similar results were obtained by Mehrpooya et al. [172], whose study involved 200 μg of Se as a supplement to conventional therapy for 12 weeks. After this period, a decrease in clinical symptoms and joint pain was observed in the Se supplementation group. A similar relationship was not observed in the placebo group [172]. Another study showed that Se supplementation caused a slight but noticeable improvement in two RA biomarkers, ESR and ACPA, compared to the placebo group [173]. A 2024 study also demonstrated the role of adequate Se supply in reducing inflammation and disease activity in patients with RA, which were assessed using the simple disease activity index (SDAI), clinical disease activity index (CDAI), and systemic immune-inflammation index (SII) [174]. Other conclusions were reached in a study conducted in 2001, where no significant differences in the course of RA were observed between the group supplemented with Se and the group receiving a placebo [175]. Due to the potential positive value of an adequate Se status in the body, patients with RA should be properly educated to avoid deficiencies of this element in their diet [176].

Furthermore, Se nanoparticles (SeNPs) show potential usefulness in the treatment of RA. They exhibit antioxidant properties by restoring the expression levels of the microRNAs (mRNAs), GPx1, CAT and inhibiting the formation of ROS, as well as anti-inflammatory properties by regulating the expression of cyclooxygenase-2 (COX-2), TNF-α, IL-1β, IL-6, and MCP-1 [177,178]. Another potentially useful molecule in the treatment of RA is lentinan-Se (LNT-Se). This compound, as surface-active SeNPs, shows increased accumulation in cells. Importantly, LNT-Se is metabolised to SeCysteine, which is incorporated into selenoproteins and thereby effectively modulates the expression of the selenoprotein GPx1, a key antioxidant enzyme. This enzyme is involved in the removal of ROS, thereby reducing oxidative stress. Oxidative stress is associated with the activation and differentiation of osteoclasts. By reducing oxidative stress and activating selenoproteins, LNT-Se significantly inhibits osteoclastogenesis, thereby preventing pathological bone loss [179]. Another study conducted on a rat model demonstrated that Se-methionine-folic acid nanoparticles (SeMetFa NPs) also show potential for use in the treatment of RA due to their ability to increase the concentration of the antioxidant enzymes GPx, SOD, and CAT in the liver, kidneys, and spleen, which reduces the concentration of the inflammatory biomarkers TNF-α, CRP, and PGE2 in the serum and reduces paw joint swelling. In addition, SeMetFa NPs showed good bioavailability and low toxicity [180]. Another way to administer Se is to use Selemax®, which is inactive Saccharomyces cerevisiae yeast enriched with organic Se. A study conducted on a mouse model showed that Selemax® reduced the number of inflammatory cells present in the knee joint cavity and caused a decrease in the levels of the pro-inflammatory cytokines: TNF-α and IL-1β; and the chemokine (CXC motif) ligand 1/keratinocyte chemoattractant (CXCL1/KC) in the periarticular tissue of mice. Clinically, there was a reduction in paw oedema [181].

However, despite the potential benefits, excessive Se supplementation can cause symptoms of Se overload, which include hair and nail loss and brittleness, digestive problems, skin rash, reduced haemoglobin levels, garlic breath, and nervous system abnormalities [182].

#### 4.1.12. The Role of B in Rheumatoid Arthritis

B has been shown to influence inflammatory pathways by modulating cytokine production (e.g., TNF-α, IL-1α, IL-6), supporting antioxidant defense systems, and regulating enzyme activity and cell membrane function [183]. In RA, where chronic inflammation leads to joint destruction, B may exert beneficial effects by dampening immune-mediated tissue injury and reducing oxidative stress.

Newnham et al. [56] reported significantly lower B concentrations in the bones and synovial fluid of patients with arthritis, including RA, compared to healthy individuals. Additionally, in geographic regions with naturally low B intake (<1 mg/day), the prevalence of arthritis was markedly higher (up to 70%) than in regions with higher intake (3–10 mg/day, prevalence <10%). Animal studies further confirmed that B supplementation reduces the severity of experimentally induced arthritis, decreases joint inflammation, and accelerates bone healing [56]. In a randomized, placebo-controlled clinical trial involving patients with RA who were maintained on etanercept, the addition of boron supplements—specifically calcium fructoborate (CFB) and sodium tetraborate (NTB)—resulted in significant clinical and biochemical improvements. After 60 days of treatment, the patients receiving B had lower disease activity scores (DAS28, SDAI, CDAI) and significant reductions in their serum ESR, hsCRP, TNF-α, IL-1α, and IL-6 levels, with CFB showing greater efficacy than NTB [183]. Beyond nutritional supplementation, B has also been investigated as a carrier in boron neutron capture synovectomy (BNCS)—a targeted radiotherapeutic approach. In a rabbit model of antigen-induced arthritis, intra-articular or intravenous administration of boronated compounds such as BPA and GB-10 achieved therapeutic concentrations (>20 ppm) in inflamed synovial tissue without significant accumulation in healthy structures. This suggests the potential for selective synovial ablation with minimal off-target toxicity [184].

Current evidence supports the hypothesis that boron supplementation may have an adjunctive role in the management of RA, especially in combination with conventional therapies. Moreover, innovative applications such as BNCS offer promising avenues for the local control of synovitis. While the existing data are encouraging, larger randomized trials and mechanistic studies are needed to fully elucidate boron’s therapeutic potential in RA.

#### 4.1.13. The Role of Si in Rheumatoid Arthritis

To determine the effect of Si on chronic inflammation in patients with RA, Prescha et al. conducted a study that demonstrated increased serum Si concentrations in patients with RA, along with decreased levels of endogenous antioxidants and elevated levels of oxidant molecules in the blood, compared to healthy controls. The increased oxidative stress seen in patients with RA is likely reduced by Si, which, by inhibiting the release of IL-6, which exacerbates inflammation and induces bone resorption by osteoclasts, helps limit inflammation and bone destruction [185].

A frequently reported problem in the literature is occupational exposure to Si compounds, which, when inhaled, accumulate in the pulmonary alveoli and activate macrophages, inducing the secretion of proinflammatory cytokines and resulting in an enhanced immune response. Si particles do not decompose, so the macrophages stimulated by them activate NADPH oxidase and increase the release of ROS, causing apoptosis and the release of Si particles. Chronic exposure can activate antigen-presenting cells and promote antibody secretion. Additionally, a decrease in Treg lymphocytes may result in chronic inflammation and an increased risk of autoimmune diseases, including RA (Caplan’s syndrome) [186,187,188]. The association between chronic occupational exposure to Si compounds and an increased risk of developing RA, as well as an increase in the severity of its course, was demonstrated in a meta-analysis conducted by Morotti et al. [189], as well as in numerous studies conducted over the last years [189,190,191,192,193].

The use of Si-filled breast implants may be an additional risk factor for the development of autoimmune diseases such as RA. A study conducted by Tervaert et al. [194] included a group of patients with silicone breast implants who experienced silicone implant incompatibility syndrome (SIIS), such as silicone allergy symptoms or systemic symptoms (arthralgia, weakness, fever). The study showed that half of the women studied experienced humoral immunodeficiency (hypogammaglobulinemia or an IgG subclass deficiency), which may result from the absorption of immunoglobulins by the silicon compounds contained in the implant and consequently lead to the development of autoimmune diseases due to an impaired humoral response. However, further research is needed to confirm this [194].

In summary, silicon may alleviate the symptoms of RA by inhibiting inflammation and oxidative stress. Studies have shown that patients with RA have elevated blood silicon levels, which may be related to the protective effects of silicone, which include the limitation of IL-6 secretion. This cytokine exacerbates inflammation and bone destruction. On the other hand, chronic exposure to Si compounds (e.g., Si dust inhalation) may increase the risk of developing RA by activating the immune system and inducing chronic inflammation.

**Table 1:** Summary of the role of trace elements in RA.

Trace Element	Level in Disease	Impact on the Disorder	Additional Information
Iron (Fe) [97,98,99,100,101,102,103,104,105]	Decreased in serum; elevated in the synovial membrane	Patients have Fe deficiency anaemia; Fe deposits may indicate a link between their presence and the pathophysiology of chronic inflammation in people with RA;	Due to the potential involvement of ferroptosis in the pathogenesis of RA, it appears to be a potential therapeutic target;
Copper (Cu) [98,107,108,109,110,111,112]	Elevated	Elevated Cu concentration may be related to chronic inflammation, in which there is increased synthesis of interleukin (IL)-1), IL-6, and tumor necrosis factor-alpha (TNF-α), stimulating hepatocytes to synthesise ceruloplasmin;	Cuproptosis may be associated with RA progression, and research is ongoing into the use of drugs that affect cuproptosis to limit the progression of RA;
Cobalt (Co) [113,114]	-	Co can generate reactive oxygen species (ROS), leading to oxidative tissue damage; Co (II) nanocomplexes with potential therapeutic applications due to their anti-inflammatory properties;	-
Manganese (Mn) [99,115,116,117,118,119,120,121,122,123,124,125,126,127,128,129,130]	Elevated/unchanged/decreased in serum; decreased in hair and synovial fluid;	Mn superoxide dismutase (MnSOD) reduces swelling; Mn-containing SOD mimetics reduce swelling, decrease damage to joint cartilage and bone, reduce inflammatory cell infiltration, and alleviate joint pain; Mn nanoparticles remove excess ROS, promote the polarisation of macrophages from the M1 to M2 phenotype; Mn nanoparticles can also serve as effective drug carriers;	-
Zinc (Zn) [99,107,108,120,121,131,132,133,134,135,136,137,138,139,140,141,142,143,144,145,146,147,148]	Decreased	Zn supplementation reduces the risk of developing osteopenia or osteoporosis; Zn aspartate and Zn citrate lower the levels of rheumatoid factor (RF), antibodies against citrullinated proteins (ACPA) and C-reactive protein (CRP), and reduce joint erosion and osteophyte formation; Zn nanoparticles reduce M1 macrophage infiltration into the synovial membrane, levels of IL-1 and TNF-α, and ACPA;	-
Silver (Ag) [149,150,151,152]	-	Ag nanoparticles exhibit therapeutic effects: anti-inflammatory and antioxidant. Reduction of swelling and inflammatory cell infiltration; reduction of pro-inflammatory cytokines such as TNF-α, IL-1β and interleukin-6; shift of macrophage polarisation from M1 to M2 phenotype; reduction in ROS and increase in superoxide dismutase (SOD) and catalase (CAT) activity; inhibition of osteoclast formation;	-
Cadmium (Cd) [153,154,155,156,158]	Elevated	Cd increases the production of ACPA and RF; Cd increases ROS production; by inhibiting the growth and inducing apoptosis of hypertrophied inflammatory synoviocytes and inflammatory effector cells Cd shows a potential role in RA treatment;	Cd is strongly associated with the formation of nodules in the lungs of patients with RA;
Mercury (Hg) [131,159,160,161,162]	Elevated	Hg disrupts the DNA function of these cells and inhibits collagen synthesis of synovial membrane cells; Hg can cause autoimmune dysfunction and systemic inflammation;	Hg sulphide nanoparticles show a potential role in the RA treatment;
Lead (Pb) [99,137,153,159,163]	Elevated	Pb causes oxidative damage, and has inflammatory and immunological properties; Pb also disrupts the function of synoviocytes by inhibiting collagen synthesis and disrupting the DNA function of these cells;	-
Nickel (Ni) [99,164,165]	Elevated	Pb disrupts antioxidant function, depletes glutathione, increases ROS production and causes inflammation.	-
Selenium (Se) [97,108,118,167,168,169,170,177,178,179,180,181]	Decreased	Se is responsible for the activity of the glutathione peroxidase (GSH-Px) enzyme; Se may also inhibit osteoclastogenesis by suppressing receptor activator of nuclear factor kappa-Β ligand (RANKL) expression on CD4+ T lymphocytes; Se nanoparticles (SeNPs) show potential usefulness in the treatment of RA because of their antioxidant and anti-inflammatory properties;	Excessive Se supplementation can cause symptoms of Se overload, which include hair and nail loss and brittleness, digestive problems, skin rash, reduced haemoglobin levels, garlic breath and nervous system abnormalities;
Boron (B) [56,183,184]	Decreased	Reduces inflammatory markers (TNF-α, IL-1α, IL-6, CRP, ESR); improves clinical scores (DAS28, CDAI, SDAI); acts as an adjuvant to biological drugs like etanercept in RA therapy.	Significantly lower B levels were found in serum, bone, and synovial fluid of RA patients; B supplements (e.g., calcium fructoborate, sodium tetraborate) improved symptoms in clinical and preclinical studies.
Silicon (Si) [185,186,187,188,189,190,191,192,193,194]	Elevated	Silicon inhibits inflammation and oxidative stress	Chronic exposure to silicon may increase the risk of developing RA

Abbreviations: ACPA—antibodies against citrullinated proteins; Ag—silver; AgNPs—silver nanoparticles; B- boron; CAT—catalase; Cd—cadmium; Co—cobalt; CRP—C-reactive protein; Cu—copper; ESR- erythrocyte sedimentation rate; FA-AgNPs—folic acid-modified silver nanoparticles; F-AgÅPs—Fructose-coated Ångstrom-scale silver particles; Fe—iron; G-AgNPs—guggul-mediated biosynthesized silver nanoparticles; GSH-Px—glutathione peroxidase; Hg—mercury; IL-1—interleukin-1; Mn—manganese; Ni—nickel; Pb—lead; RA—rheumatoid arthritis; RANKL—receptor activator of nuclear factor kappa-Β ligand; RF—rheumatoid factor; ROS—reactive oxygen species; Se—selenium; SeNPs—selenium nanoparticles; Si—silicon; SOD—superoxide dismutase; TNF-α—tumor necrosis factor α; Zn—zinc.

### 4.2. The Role of Trace Elements in Osteoarthritis

OA is a chronic degenerative joint disease characterised by an imbalance between the resilience of the cartilage and the mechanical forces exerted upon it. The progressive degeneration of the cartilage results in mild synovial inflammation, subchondral bone sclerosis, and, ultimately, the formation of osteophytes [195]. In 2020, 595 million people around the world suffered from OA, a number which is predicted to increase by 74.9% by 2050 [196]. Common symptoms include joint pain, swelling, and stiffness. Gradually, these symptoms may exacerbate, potentially resulting in disability [197]. Risk factors include a high BMI, advanced age, female gender, and abnormal joint loading. Furthermore, malalignment is particularly significant in cases of knee OA [198].

#### 4.2.1. The Role of Fe in Osteoarthritis

The association between Fe overload disorders, including hereditary hemochromatosis, myelodysplastic syndromes, thalassemias, and hemoglobinopathies, and the onset of OA is well documented (Table 2). Elevated Fe levels, as measured by either serum concentration or transferrin saturation, have been associated with an increased risk of developing OA, particularly in larger joints such as the knee and hip. Furthermore, a positive correlation between total hip replacement and Fe levels has been established. Fe exhibits a distinctive capacity to disrupt joint homeostasis, which is implicated in the pathogenesis of OA [199,200,201]. Another piece of evidence for Fe’s role in primary OA pathogenesis comes from studies on disease-prone guinea pigs. These animals showed lower histologic OA scores when fed a Fe-deficient diet for 19 weeks, and these were accompanied by decreased disintegrin and metalloproteinase with thrombospondin motifs (ADAMTS) 4 in their synovium [202].

A recently identified novel form of cell death—ferroptosis [203]– has been suggested to influence the development of OA. Ferroptosis is characterised by the Fe-dependent accumulation of lipid hydroperoxides that attain cytotoxic levels. Morphologically, the affected cells demonstrate an aberrant mitochondrial ultrastructure, as observed through electron microscopy, which reveals a reduction in the mitochondrial volume, an increase in membrane density, and the disappearance of mitochondrial cristae. The subsequent formation of lipid peroxides, alongside a compromised antioxidant system, directly contributes to the initiation of ferroptosis. Excess Fe can also produce ROS through the Fenton reaction and activation of Fe-containing enzymes, such as lipoxygenase, which promotes lipid peroxidation [204,205].

The byproducts of lipid peroxidation, specifically malondialdehyde and 4-hydroxynonenal, form adducts with proteins and DNA, which results in cytotoxic effects and cellular dysfunction. Furthermore, Fe contributes to or worsens the progression of cartilage damage. In conditions of excess Fe, this element accelerates chondrocyte apoptosis, which in turn leads to the expression of matrix-degrading enzymes such as MMP3 and MMP13 and a reduction in type II collagen [206,207,208]. Intracellular Fe overload can also M1 polarise synovial macrophages by phosphorylating 4E-BP1 in the mTORC1-p70S6K/4E-BP1 pathway. This leads to increased production of proinflammatory agents such as IL-6 and TNF-α, which exacerbate cartilage damage [209]. Elevated Fe levels in the synovium of OA patients influence gene expression (such as that of JUN and ZFP36), further promoting ferroptosis, disrupting mast cell activation, altering macrophage polarisation, and facilitating immune infiltration of the synovium [210].

#### 4.2.2. The Role of Cu in Osteoarthritis

Cu demonstrates a complex and only partially understood relationship with osteoarthritis that involves multiple signalling pathways and interactions with crucial proteins [211]. Elevated serum Cu levels were positively correlated with an increased risk of osteoarthritis [212]. Similar observations were made regarding higher genetically predicted Cu levels [213]. Additionally, patients with osteoarthritis exhibited significantly higher concentrations of Cu in their synovial fluid compared to a healthy control group. Moreover, a positive correlation has been proven between the concentrations of Cu and Zn in synovial fluid [97,214]. However, some studies have found the connection between Cu levels and OA to be weak [215].

At the molecular level, Cu may contribute to cartilage damage through multiple mechanisms. One notable example is Cu-mediated cell death, referred to as cuproptosis, which occurs following the disruption of Cu homeostasis through various pathways. Studies have demonstrated that, in transgenic mice with an ATP7B knockout, a model for Wilson’s disease, an overload of Cu resulted in the fragmentation of cardiolipin (a crucial element of mitochondrial membranes). Furthermore, Cu can interact with hydrogen peroxide and reducing agents through Fenton-like chemistry, forming hydroxyl radical species. These species have been shown to induce lipid fragmentation and loss of mitochondrial cristae. Additionally, Cu can disrupt the tricarboxylic acid cycle by reducing the activity of the essential enzyme, citrate synthase. Moreover, as Cu serves as a cofactor for complex IV in cellular respiration, Cu deprivation can lead to decreased ATP synthesis, mitochondrial swelling, and, ultimately, apoptosis [216].

Dysregulated Cu(I) in the matrix could impair the maturation of [4Fe-4S] clusters (cofactors found in a wide variety of proteins, especially in redox enzymes, e.g., respiratory chain complexes I and III) by substituting the Fe bound to cysteine residues in iron-sulfur cluster assembly 2 (ISCA2) with a high binding affinity. The inhibition of complex I could be associated with the decrease in Fe-S cluster synthesis [217]. Cu(I) is linked to the activation of iron regulatory protein 1 (IRP1), the protein involved in Fe uptake from the cytosol. The Fe accumulated in mitochondria was indicated to oxidise Cys-containing peptides and increase intracellular oxidative stress. Cu levels also correlate with higher levels of inflammatory cytokines (IL-6 and CRP) [218].

#### 4.2.3. The Role of Co in Osteoarthritis

Prosthetic components used in total joint replacement (TJR)—a common intervention for osteoarthritis—are often composed of Co-containing metal alloys due to their superior strength, wear resistance, and hardness. These materials are engineered to be passivated by a surface layer of metal oxides or other protective films that minimise ion release [219]. However, in practice, this protective barrier can be compromised by electrochemical corrosion, mechanical wear—especially in highly loaded joints such as the knee and hip—and tribocorrosion. As a result, metal ions and wear debris can be released into surrounding tissues and enter systemic circulation, which leads to a condition known as metallosis, which is associated with both local and systemic toxicity [220,221,222].

Locally, the dispersion of Co particles within the joint environment can provoke inflammatory responses in bone and soft tissue. Clinically, this often manifests as pain, joint weakness, and instability, which may necessitate surgical revision. Elevated serum Co levels have been correlated with increased postoperative pain severity [223]. Systemically, Co ions may disseminate via the bloodstream, contributing to a range of adverse effects, including neuropsychiatric disorders, cardiomyopathy, vision and hearing loss, nephrotoxicity, and thyroid dysfunction [219].

Notably, Co-related metallosis may be more common than anticipated. One study reported that over half of its subjects developed cobalturia following TJR, with elevated Co levels being found in the urine, blood, and synovial fluid—each of which was correlated with the others [224].

In vitro studies have shown that Co^2+^ exerts concentration-dependent biological effects. At low to moderate sub-toxic concentrations, Co^2+^ exhibits anti-inflammatory properties, including suppression of IL-1β expression in chondrocytes and promotion of M2 macrophage polarisation [225]. In contrast, higher concentrations trigger pro-inflammatory responses, including increased expression of TNF-α and IL-6, M1 macrophage polarisation, and activation of the NF-κB signalling pathway. Additionally, elevated Co^2+^ levels activate the phosphoinositide 3-kinase/protein kinase B (PI3K/Akt) pathway, upregulating TLR4 membrane receptor expression and further reinforcing the M1 inflammatory phenotype [226].

#### 4.2.4. The Role of I in Osteoarthritis

The role of I in the pathogenesis of osteoarthritis remains inadequately characterised. Existing investigations that propose a potential involvement are limited in scope, and are often derived from studies conducted within small endemic populations or based on animal models.

Moreno-Reyes et al. [227] conducted a study on a group of Tibetan children and compared the relationship between I and Se deficiencies and the occurrence of Kashin–Beck disease (KBD). KBD is an endemic arthropathy that occurs in certain regions of China, North Korea, and Siberia, usually affecting children aged 5–15. It manifests as increasing joint deformity, chronic pain, limited mobility, and, in more severe cases, necrosis of the joint cartilage and bone growth plates, which leads to impaired growth. The study showed that I deficiency in the children’s diet was a risk factor for developing KBD. This may be due to hypothyroidism secondary to I deficiency, which disrupts normal bone development [227,228].

A study on rats found that a combination of Se and I deprivation impaired bone and cartilage formation [229]. On the other hand, a study conducted on rats showed that chronic I overdose causes damage to the joint cartilage and epiphyseal growth plates, increasing the risk of developing osteoarthritis [230].

#### 4.2.5. The Role of Mn in Osteoarthritis

Mn may have a protective effect on the development of OA [231], probably due to its activity as a cofactor of many enzymes, including mitochondrial SOD2 and glycosyltransferase (GTF). SOD2 is a strong antioxidant that neutralises ROS, reducing oxidative stress induced by inflammation and protecting cartilage from damage, while GTF participates in the synthesis of glycosaminoglycan, proteoglycan, and type II collagen, which builds cartilage and supports its regeneration [58]. This is probably why the correct concentration of Mn can slow down cartilage degeneration, which is why new forms of treatment are being tested in which the combination of kinesiotherapy and Mn supplementation allows for better treatment effects and a reduction in symptoms in patients with OA [232].

The results of a meta-analysis conducted by Haoyan Shi et al. [233] showed that patients with OA have lower serum Mn levels, which confirms the findings of previous studies; however, this research did not show a causal relationship between low Mn levels and the development of OA [118,233]. Additionally, it has been shown that chronic exposure to Cd may negatively affect Mn levels and its activity in cartilage, leading to its degradation [234].

In the study by Fernandez-Moreno et al. [235], increased levels of SOD2 were found in patients with OA, which confirms the increased level of oxidative stress that results from ongoing inflammation [235]. In the study by Ostalowska et al. [236], it was suggested that, with an increasing ROS concentration, the antioxidant activity decreases, which was measured using antioxidant enzyme activity measurements. The results of studies on patients with OA show increased activity of antioxidant enzymes, which is contrary to the hypothesis of this and previous studies, although this may be the result of measurements being made in a different period of disease activity than was previously conducted. Probably in the initial stage of the disease, when acute inflammation dominates, there is a significant accumulation of ROS in the synovial fluid, and consequently, an increase in the activity of antioxidant enzymes, whereas, with the progression of the disease, antioxidant reserves are depleted and their activity decreases [236].

Decreased Mn levels may also be caused by overexpression of the Zn transporter ZIP8, which causes Zn accumulation in the cartilage and reduced Mn resorption in the kidneys, resulting in the predominance of cartilage-degrading factors [237].

#### 4.2.6. The Role of Zn in Osteoarthritis

The role of Zn levels in OA remains controversial. Some recent studies report elevated Zn levels in the serum of OA patients. Higher dietary intake of Zn also correlated with an increased risk of OA [238,239]. However, those results were not achieved in all studies, as some did not find elevated Zn levels in serum but only in synovial fluid [214,240]. Furthermore, a study by Jakoniuk et al. [241] found that, in the case of spine OA, patients have lower Zn levels than healthy controls [241]. A meta-analysis by Shi et al. [233] concluded that the correlation between Zn and OA risk does not reach statistical significance [233]. 

At the molecular level, Zn may contribute to the pathogenesis of OA through multiple mechanisms. Notably, upregulation of the Zn influx transporter Zrt- and Irt-like Protein 8 (ZIP8), an enzyme involved in the Zn-ZIP8-MTF1 axis in chondrocytes, has been observed in OA cartilage [242]. Pro-inflammatory cytokines and HIF molecules upregulate it. Hypoxia-inducible factor 1 alpha (HIF-1α) and HIF-2α are homologs that undergo oxygen-dependent degradation and are involved in hypoxic responses of the cell. One of these responses effects catabolic target genes, coding matrix-degrading enzymes. HIF-2α increases Zn^2+^ influx in OA chondrocytes by activating the Zn-ZIP8-MTF1 cascade, which produces downstream transcription factor MTF1, a novel transcriptional regulator of HIF-2α, creating a vicious cycle. This sequence of events enhances the activity of matrix-degrading enzymes such as MMP (MMP-3 and MMP-13) and ADAMTS-5, thereby promoting extracellular matrix breakdown, which leads to cartilage damage. Furthermore, the HIF-2α pathway amplifies inflammatory mediators such as IL-6 and nicotinamide phosphoribosyltransferase (NAMPT), further activating MMPs [243].

In addition to its regulation of ZIP8 and MTF1, Zn plays a role in pathogenesis by supporting the activity of Zn-dependent MMPs, particularly MMP-13, which are crucial to cartilage matrix degradation. Metallothioneins (MTs), which regulate intracellular Zn levels and oxidative stress, may also contribute to cartilage protection, although their role is more clearly defined in RA. Zn affects redox-sensitive pathways such as NF-κB, indirectly mitigating inflammation and oxidative damage. Genetic variants in Zn-related genes, including Zn-finger proteins such as zinc finger protein 345 (ZNF345), have been linked to susceptibility to OA. Furthermore, Zn deficiency amplifies OA risk factors, including an impaired immune response, altered hormone levels, and diminished bone integrity, particularly among older adults [154].

#### 4.2.7. The Role of Cd in Osteoarthritis

Cd exposure is a recognised risk factor for the development and progression of OA [58,244]. Elevated serum Cd levels have been correlated with both the duration and severity of OA, particularly in patients with spinal involvement, who consistently show higher levels compared to healthy controls. Cd contributes to several hallmark features of OA, including cartilage degeneration, osteophyte formation, subchondral sclerosis, synovitis, and chondrocyte apoptosis [245]. Importantly, Cd is considered a mechanistic link between cigarette smoking and OA, as it is primarily absorbed through the respiratory tract (via smoking) and the gastrointestinal tract (mainly through the consumption of seafood and rice). It is estimated that smoking one pack of cigarettes deposits approximately 2–4 µg of Cd into the lungs. Once absorbed, Cd tends to accumulate in bones and joints, where it exerts toxic effects [137,231,246].

At the molecular level, Cd influences joint health through several pathways. In vitro studies indicate that its effects on osteoblasts depend on both the concentration and cell differentiation stage. Cd can inhibit or stimulate osteoblast proliferation, suppress type I collagen synthesis, and modulate gene expression, including upregulation of the expression of *bone morphogenetic protein 2* (BMP2) and *fibroblast growth factor 1* (FGF1). Cd downregulates most bone formation markers at higher concentrations except integrin β1, which is essential for osteoblast adhesion to collagen. Alterations in integrin expression are believed to contribute significantly to Cd’s toxicity in bone tissue [247]. In vivo, Cd exposure has been shown to reduce the bone mineral density and disrupt the microarchitecture in animal models, particularly by impairing osteoblast proliferation, differentiation, and mineralisation [248].

Cd also promotes matrix degradation by upregulating MMP1, MMP3, MMP9, and MMP13 and decreasing the expression of cartilage matrix genes such as COL2A1 and aggrecan core protein (ACAN). These effects coincide with a reduction in glycosaminoglycans and proteoglycans, primarily driven by increased inflammatory signalling (IL-1β, IL-6) and oxidative stress. Mechanistically, Cd displaces Fe^2+^ and Cu^2+^ from various enzymes and proteins, enhancing hydroxyl radical formation and interfering with antioxidant defences like glutathione, catalase, and SOD [246]. Studies on cell cultures also demonstrated that Cd elevates the expression of Bcl-2-associated X-protein 1 (BAX1), *Bcl-2 antagonist/killer 1* (BAK1), *caspase 3*, *and caspase 9*—key apoptotic molecules of the intrinsic pathway [249]. Additionally, Cd exposure leads to the depletion of essential trace elements (Zn, Ni, Fe, Mn, Cr) in chondrocyte cultures—elements that vital for maintaining cartilage integrity [234].

#### 4.2.8. The Role of Hg in Osteoarthritis

The relationship between Hg exposure and OA is controversial. Experimental studies on animals have demonstrated that Hg tends to accumulate within synovial cells and articular chondrocytes, tissues that are frequently impacted in OA and RA. Notably, methylmercury has been identified as altering adipokine secretion from adipose tissue, which results in increased levels of resistin, adiponectin, ROS, and 4-hydroxynonenal (4-HNE), all of which may contribute to localised joint inflammation. Furthermore, Hg might induce epigenetic modifications of MMP and increase the level of MMP-9, a mechanism progressively acknowledged in OA’s pathogenesis [41,250,251]. Additionally, Hg accumulates in bone tissue, particularly in older individuals, which raises concerns regarding its potential long-term effects on skeletal and joint health [252].

Notwithstanding these biological insights, the existing epidemiological evidence does not substantiate a clear association between Hg exposure and OA-related outcomes, such as pain severity, radiographic changes, or overall disease risk. Additional research is needed [231,253].

#### 4.2.9. The Role of Pb in Osteoarthritis

Pb is a known environmental pollutant that has been studied for its neurotoxicity and systemic effects. Its possible role in OA has been illustrated in clinical cases involving retained intraarticular Pb bullets, which lead to arthritis, chronic synovitis, and even systemic Pb poisoning [254].

In a population study by Nelson, Shi et al. [255], elevated serum Pb levels correlated with increased odds of knee OA. Each one-unit rise in serum Pb is linked to a 26% higher radiographic OA risk. The risk of bilateral OA increased by 32% per unit of Pb. For symptomatically defined OA, a one-unit Pb increase was associated with a 16% rise in the likelihood of having OA, a 17% increase in severe symptoms, and a 25% rise in bilateral knee symptoms. Further investigation by the same researchers revealed that Pb levels are related to bone and cartilage turnover biomarkers. In women, Pb levels correlated with mineralised cartilage metabolism, while, in men, both mineralised and unmineralized cartilage turnover were affected [255]. Another cross-sectional study of 90 Egyptian adults, based on clinical symptoms and radiographic changes, found a significant correlation between serum Pb levels and knee OA severity [256]. All these findings suggest Pb may be a risk factor that contributes to OA pathogenesis.

Animal studies provide further evidence for a possible role of Pb. Pb appears to alter systemic bone remodelling processes. In a murine model, Carmouche et al. [257] exposed mice to environmentally relevant concentrations of Pb for six weeks before inducing tibial fractures. The Pb-exposed mice exhibited delayed healing, reduced collagen II formation, delayed cartilage calcification, and prolonged cartilage maturation. At higher exposure levels, fibrous nonunions developed, pointing to impaired endochondral ossification. These findings suggest that Pb disrupts the bone and cartilage turnover, processes that are also implicated in OA progression [257].

Both low and high levels of Pb exposure in vivo led to fibrillation and degeneration of the articular surface. In both in vivo and in vitro settings, Pb exposure reduced the expression of type II collagen—a marker of healthy cartilage—and increased the expression of type X collagen, which is associated with chondrocyte hypertrophy and matrix degradation. In parallel, the activity of MMP-13 and the expression of active caspases 3 and 8 rose dose-dependently. Additionally, Pb suppressed TGF-β signaling (essential for maintaining chondrocyte phenotype and cartilage integrity). These effects reflect that Pb exposure may phenotypically shift chondrocytes toward a degenerative state [258]. On a molecular level, chronic Pb exposure activates the NF-κB signaling pathway and exacerbates oxidative stress, ultimately promoting cell apoptosis [259]. In zebrafish models, Pb exposure led to structural cartilage abnormalities, bone loss, a shorter body length, and an increased deformity rate. Gene expression analyses revealed downregulation of chondrocyte- and osteoblast-related genes and inhibition of bone matrix maturation and mineralisation. Pb also promoted osteoclast formation and disrupted the growth hormone/insulin-like growth factor 1 (IGF-1) axis, factors that are critical to skeletal growth and joint maintenance [260].

Pb has been shown to accumulate at the tidemark—the transition zone between non-calcified and calcified cartilage. Pb has a higher affinity than calcium for binding to osteocalcin and can interfere with Ca^2+^ signaling within cells by competing for calcium-binding sites. Furthermore, Pb may displace calcium from hydroxyapatite crystals, potentially altering the material properties of cartilage and bone. These interactions could compromise the structural integrity of cartilage and bone and also, contribute to joint degradation [261].

#### 4.2.10. The Role of Ni in Osteoarthritis

Ni is frequently used in orthopedic alloys, particularly in joint prostheses. Through mechanical wear, corrosion, and tribocorrosion, Ni ions may be released into surrounding tissues and the bloodstream. Numerous studies have indicated elevated Ni concentrations in serum and urine up to 13 years following arthroplasty, both with metal-on-metal and metal-on-polyethylene prostheses [262,263]. However, the findings display inconsistency; some studies reveal only transient or marginal elevations, and these are often confined to isolated cases [264]. Despite these discrepancies, Ni tends to accumulate preferentially in connective tissues, including joints and menisci, which has raised concerns regarding its potential involvement in the pathogenesis of OA, particularly considering its known molecular effects. Additionally, its accumulation in tissues correlates with other trace elements such as Cd, Pb, Cu, Zn, Mg, and Ca. Significantly, smoking has been demonstrated to further augment the tissue Ni content [265].

At the molecular level, several in vitro investigations propose that Ni may contribute to OA through multiple pathways. Osteoarthritic osteoblasts exposed to Ni ions (Ni^2+^) at concentrations as low as 0.25 mM exhibit cytotoxicity and nuclear accumulation, which potentially results in DNA damage and leads to cell apoptosis. These cells also display increased expression of pro-inflammatory cytokines, such as IL-1β and IL-8, in response to Ni; this could be attributed to prior sensitisation or the intrinsic inflammatory nature of OA. Furthermore, exposure to Ni-titanium alloy has been evidenced to upregulate genes associated with inflammation and cartilage degradation, including IL-1β, IL-6, IL-8, and MMP-1, while simultaneously downregulating MMP-2, all of which are pivotal in the pathology of OA [266]. Ni additionally escalates oxidative stress, subsequently contributing to chondrocyte apoptosis and extracellular matrix degradation [156].

#### 4.2.11. The Role of Se in Osteoarthritis

Despite the well-established association between Se deficiency and KBD, the literature concerning its role in OA remains inconclusive. Se is hypothesised to exert a protective effect against OA due to its antioxidant and anti-inflammatory properties. Evidence supporting this hypothesis derives from in vitro studies conducted on both human chondrocytes and animal models which suggest that Se may alleviate OA-related damage [49,267].

Molecularly, Se activates the nuclear factor erythroid 2-related factor 2 (Nrf2) signaling pathway, an antioxidant defence regulator. This activation enhances the expression of enzymes involved in glutathione synthesis, including the catalytic and modifier subunits of glutamate-cysteine ligase and glutathione synthetase. Se also increases the levels of antioxidant enzymes, such as superoxide dismutase, glutathione peroxidase, and glutathione reductase, collectively reducing the oxidative stress within joint tissues. It acts by suppressing the activation of the NF-κB pathway, which results in decreased expression of pro-inflammatory cytokines (IL-1β, TNF-α, IL-17A) and matrix-degrading enzymes (MMP-1, MMP-3, MMP-9, MMP-13, and ADAMTS-4). In rat models, Se supplementation for two weeks resulted in reduced cartilage degradation, increased glutathione synthesis, and lower levels of inflammatory cytokines [54].

An inverse relationship has been documented between one of the selenoproteins—selenoprotein P (SELENOP), the principal Se transport protein—and the functional status in OA, which substantiates that Se levels may impact disease severity [268]. In patients with KBD, the Se concentrations in blood and hair are markedly diminished, correlating with reduced GPx activity and heightened oxidative stress markers, including nitric oxide synthase and malondialdehyde. This oxidative imbalance contributes to chondrocyte apoptosis, as evidenced by elevated levels of p53, B-cell lymphoma 2 (Bcl-2), and BAX, as well as increased expression of caspases 9 and 3 [269].

Comparable findings are observed in mice subjected to a Se-deficient diet, which results in decreased GPx1 mRNA expression and enzyme activity within the liver. These animals also exhibit a reduced femoral trabecular bone volume and number, heightened trabecular separation, and elevated serum markers such as CRP, tartrate-resistant acid phosphatase, and intact parathyroid hormone. These alterations reflect increased bone resorption, a compromised bone microstructure, and impaired antioxidant defence [270]. Furthermore, Se has been demonstrated to promote the proliferation and differentiation of chondrogenic progenitor cells [58]. 

However, epidemiological studies have yielded conflicting results. Some have observed no significant association between Se levels and OA risk, disease progression, pain, or radiographic changes [231,241,267]. Notably, McClung et al. [271] reported that excessive Se intake could be detrimental, as it promotes overexpression of GPx1, which interferes with insulin signaling pathways [271]. Overexpressing GPx1 can lead to hyperglycemia and obesity, which are recognized contributors to systemic inflammation and the pathogenesis of OA [238,272].

#### 4.2.12. The Role of B in Osteoarthritis

B is an element that performs various functions in the body, including supporting proper osteogenesis, acting as an antioxidant, reducing the secretion of pro-inflammatory cytokines, and influencing the hormonal balance by increasing the bioavailability and half-life of vitamin D, estrogen, and testosterone [273,274,275].

A significant reduction in the B concentration has been observed in patients with OA, in the serum, bone, and synovial fluid, compared to healthy controls. The B concentration has also been shown to decrease with the disease’s exacerbation and duration [56,58,276]. Additionally, studies conducted on children with OA have demonstrated reduced B concentrations, likely resulting from the consumption of crops grown in soils that are deficient in this element [277,278].

Due to the likely impact of B deficiency on the development of OA, research is ongoing into the use of B supplementation as a therapy to treat and alleviate OA symptoms. Clinical studies conducted in rats using intra-articular B administration have yielded promising results, confirming the anti-inflammatory and regenerative effects of this element. Positive results have also been obtained in patients with OA, for whom oral B supplementation enabled remission of symptoms such as joint pain and itching, while maintaining patient safety [56,58,59,275].

### 4.3. The Role of Trace Elements in Psoriatic Arthritis

PsA is a systemic inflammatory disease that affects the skin, joints, and tendon attachments. It affects 0.1–1% of the general population worldwide, and approximately 20% of people with psoriasis [279]. Typically, joint symptoms appear years after the onset of skin lesions, but in rare cases, PsA may precede skin manifestations. It is characterised by a chronic and progressive course. If left untreated, PsA can lead to permanent joint damage, significant disability, and a reduced quality of life [280]. The pathogenesis of PsA is multifaceted and complex. It results from the interaction of certain factors, such as genetic predisposition, environmental factors, and immune system dysregulation [281].

#### 4.3.1. The Role of Fe and Cu in Psoriatic Arthritis

In a 1984 study by Oriente et al. [282], the researchers sought to determine whether Fe and Cu concentrations could serve as auxiliary laboratory indicators for PsA and how their levels change in different forms of the disease. To achieve this, they examined the concentrations of Fe and Cu, as well as the serum ceruloplasmin concentrations, in 45 patients with PsA. This group consisted of 20 patients with polyarticular PsA, 12 with mono- or oligoarticular PsA, and 13 with spondyloarthropathic PsA. The mean serum concentrations of Cu, Fe, and ceruloplasmin were significantly increased in the patients with PsA compared to the control group of 60 healthy individuals and to a group of 63 patients with only psoriasis. The number of affected joints showed a significant correlation with changes in the serum concentrations of these parameters. In the polyarticular subgroup, serum Cu, Fe, and ceruloplasmin concentrations were increased, in the mono- or oligoarticular subgroup only Cu and ceruloplasmin concentrations were significantly increased, and, in the spondyloarthropathic form, no significant changes in their concentrations were observed. This would suggest that these indicators may prove potentially helpful in differentiating between forms of PsA [282] (Table 3). In contrast, another study in which 76 people with PsA were tested for the levels of selected trace elements in their serum, including Cu, showed that the concentration of Cu was lower compared to that in healthy adults. In addition, Cu showed a positive correlation with the CRP levels and ESR. Patients with CRP-positive PsA had higher serum Cu concentrations and a higher Cu–Zn ratio than patients with CRP-negative PsA. Higher Cu levels were also observed in patients with a positive ESR compared to those with a negative ESR. In individuals in PsA remission, lower serum Cu concentrations and a lower Cu–Zn ratio were observed compared to the patients with ongoing active disease. These results may suggest the use of Cu as a marker for monitoring disease activity and treating patients with PsA [283]. However, another study that measured the Cu concentrations in 37 individuals with PsA found significantly higher results compared to a group of 50 healthy individuals. Interestingly, after intravenous administration of MTX to patients, this concentration decreased. A therapeutic effect of normalising the Cu concentration in PsA has been suggested [284]. High Cu levels in patients with PsA are also confirmed by a 1995 study in which the concentration of this element was measured in 25 patients with PsA and compared with a control group of 25 individuals [285].

#### 4.3.2. The Role of Mn in Psoriatic Arthritis

As mentioned above, genetics is also one of the predisposing factors for PsA. In a study by Yen et al. from 2004 [286], the researchers sought to determine the impact of MnSOD gene polymorphism on the pathogenesis of PsA. MnSOD is an enzyme that contains Mn as a cofactor and which is encoded by the SOD2 gene. It plays an important role in protecting cellular components from oxidative damage, which intensifies during periods of stress or inflammation. Polymorphisms in the SOD2 gene can affect the activity of MnSOD and its expression levels. The study showed that the frequency of the MnSOD 1183C/T genotype was significantly higher in 52 patients with PsA compared to the control group of 90 healthy individuals. This suggests that patients with this genotype may be more susceptible to developing PsA. Conversely, the frequency of the homozygous MnSOD 1183T/T genotype was reduced in the group of patients with PsA compared to the control group. This may indicate that individuals who inherit two copies of the ‘T’ allele at this specific locus are potentially less susceptible to developing PsA. In contrast, the frequency of the MnSOD 1183C phenotype was significantly increased in patients with PsA compared to healthy individuals. This suggests that MnSOD 1183C polymorphisms may promote the development of PsA [286].

#### 4.3.3. The Role of Zn in Psoriatic Arthritis

Another trace element analysed in our study is Zn. In the study cited above for Cu, the Zn levels were also examined in a group of 76 people with PsA. As in the case of Cu, patients with PsA showed Zn deficiency compared to healthy European adults. However, no correlation was observed between the CRP level or ESR and the Zn level in patients with PsA [283]. In the MTX study, which was also mentioned in relation to Cu, low serum Zn levels were observed in people with PsA compared to the control group. After intravenous administration of MTX to patients, the Zn concentration increased significantly [284]. Therefor, ite appears that Zn may have a therapeutic effect in PsA. This issue was already discussed in 1980. Patients with PsA were given oral Zn sulphate. After this therapy, a reduction in the severity of clinical symptoms such as joint pain and swelling was observed, and, consequently, the need for pain medication decreased. A reduction in serum immunoglobulins (especially IgA and IgM) was also noted. Importantly, the treatment was well tolerated, and no serious adverse effects were reported [287]. However, in a study of 13 people with psoriasis and 13 people with PsA who underwent 6 weeks of Zn sulphate therapy, the effect on the course and activity of PsA was minimal [288]. Further studies are needed to determine whether Zn sulphate may be valuable in the treatment of PsA.

#### 4.3.4. The Role of Cd in Psoriatic Arthritis

A study of patients with IA, including PsA, showed increased blood Cd levels (≥0.65 μg/L) compared to individuals without IA. This result was positively associated with serum inflammatory markers such as ESR, hs-CRP, and COX-2. Furthermore, high Cd concentrations were associated with the disease severity in patients with IA. Over 84% of the IA patients with high blood Cd levels experienced disease exacerbation, while only 15.8% were in remission [289].

#### 4.3.5. The Role of Se in Psoriatic Arthritis

A study of 76 patients with PsA showed, as in the case of Cu and Zn, the presence of Se deficiency in the study group compared to healthy European adults [283]. Measurement of the Se concentrations in a group of 25 patients with PsA also showed low results compared to the control group [285]. Low Se concentrations in the blood of patients with PsA are also confirmed by a cross-sectional study of 272 patients with inflammatory rheumatic diseases of the musculoskeletal system. Among the subjects were 67 people with PsA. The levels of Se, SELENOP, which is a Se transporter, and GPx3, which is responsible for antioxidant activity, were measured. In patients with PsA, in addition to a reduced Se concentration, there was also a reduced level of SELENOP and low GPx3 activity. A positive correlation was observed between the serum GPx3 activity and SELENOP. These results may indicate the role of Se in the pathophysiology of PsA and its potential usefulness in the diagnosis of the disease [268]. In a group of 40 people with PsA who were treated with MTX monotherapy or anti-TNF ± MTX, the Se levels were measured at the beginning, after 6 weeks, and after 6 months of the study to determine the effect of anti-rheumatic therapy on Se levels. An increase in Se concentration was observed, which was visible after 6 weeks and persisted after 6 months. No correlation was observed between the baseline Se level and inflammatory parameters such as the ESR and CRP level, but an increase in its level already causes a decrease in these indicators. This indicates the potential role of Se in the body’s response to therapy, which may help in monitoring patients during treatment and suggests considering possible nutritional support [290].

**Table 3:** Summary of the role of trace elements in PsA.

Trace Element	Level in Disease	Impact on the Disorder	Additional Information
Iron (Fe) [282]	Elevated	-	Elevated Fe concentrations were observed only in the form of polyarticular disease;
Copper (Cu) [282,283,284,285]	Elevated [282,284,285]/decreased [283]	Possible impact on the pathogenesis of imflammation; potentially useful in monitoring disease activity and treatment progress;	High Cu concentrations were observed in both polyarticular and mono- or oligoarticular forms; Higher Cu concentrations were observed in patients with positive inflammatory markers: erythrocyte sedimentation rate (ESR) and C-reactive protein (CRP); Intravenous administration of methotrexate (MTX) reduces Cu concentrations;
Zinc (Zn) [283,284,287,288]	Decreased	The possible therapeutic effect of Zn sulphate-administered orally, it reduces clinical symptoms. However, its role is unclear;	Intravenous administration of MTX causes an increase in Zn concentration;
Cadmium (Cd) [289]	Elevated	Possible influense on the pathogenesis of inflammation;	Cd levels were positively linked to serum inflammatory markers like ESR, CRP and cyclooxygenase-3 (COX-2);
Selenium (Se) [268,283,285,290]	Decreased	Possible impact on the pathogenesis of inflammation. Potentially useful in monitoring patients during treatment;	Reduced levels of selenoprotein P (SELENOP) and low activity of glutathione peroxidise 3 (GPx3) were also observed; MTX therpy causes an increase in Se concentration. Increased Se levels result in a decrease in CRP and ESR;

Abervariattions: Ag—silver; Cd—cadmium; COX-2—cyclooxygenase-2; CRP—C-reactive protein; Cu—copper; ESR—erythrocyte sedimentation rate; Fe—iron; GPx3—glutathione peroxidase 3; MTX—methotrexate; Se—selenium; SELENOP—selenoprotein P; Zn—zinc.

### 4.4. The Role of Trace Elements in Ankylosing Spondylitis

AS is a chronic inflammatory rheumatic disease that primarily affects the axial skeleton, particularly the sacroiliac joints and spine, and often leads to progressive structural damage and ankylosis. Clinically, AS is characterised by inflammatory back pain, morning stiffness, and a gradual decline in spinal mobility, with its onset typically occurring in the third decade of life and a higher prevalence among males (male–female ratio approximately 2:1). The pathogenesis of AS is multifactorial and remains only partially understood. It is believed to result from a complex interplay of genetic predisposition, immune dysregulation, and environmental influences. The strong association between AS and the HLA-B27 allele is well documented, with up to 90% of affected individuals testing positive for this marker. However, HLA-B27 alone does not account for all cases, and genome-wide association studies have identified numerous non-HLA genes, including endoplasmic reticulum aminopeptidase 1 (ERAP1) and interleukin-23 receptor (IL23R), that contribute to susceptibility to this disease [291].

#### 4.4.1. The Role of Fe in Ankylosing Spondylitis

Fe metabolism is increasingly recognised as an important factor in the immunopathogenesis of AS (Table 4). Both Fe overload and its regulatory imbalance may contribute to inflammation, oxidative stress, and tissue damage in AS, although findings vary across clinical and genetic studies. Studies have shown that the intracellular Fe levels in granulocytes and platelets are significantly elevated in patients with AS compared to healthy controls. This cellular Fe overload is positively correlated with inflammatory markers such as CRP levels and the ESR, which suggests that Fe may amplify inflammatory responses through enhanced ROS generation and altered immune cell function [292]. Ferroptosis, an Fe-dependent form of regulated cell death characterised by lipid peroxidation and mitochondrial dysfunction, has been proposed as a potential mechanism of tissue injury in inflammatory arthritis, including AS. Fe-driven oxidative stress promotes damage to joint structures and sustained chronic inflammation. Evidence from AS tissues supports the involvement of ferroptosis through the elevation of oxidised lipid levels and mitochondrial injury markers [293]. Fe homeostasis modulates immune function by affecting macrophage polarization, T-cell responses, and cytokine secretion. Excess Fe may suppress antigen presentation and promote a Th2-skewed immune profile. While this may have regulatory roles, in chronic conditions like AS it could also contribute to immune dysregulation and persistent inflammation [292]. Two Mendelian randomisation (MR) studies explored the causal relationship between systemic Fe levels and AS risk, yielding conflicting results: Wang et al. [294] reported a positive causal association between genetically determined serum Fe levels and AS risk, indicating that elevated Fe levels may increase susceptibility to AS [294]. In contrast, Yang et al. [295] found no significant genetic association between AS and four Fe-related biomarkers (ferritin, serum Fe, total iron binding capacity (TIBC), transferrin saturation (TSAT)), concluding that Fe status is likely not a genetic cause of AS [295].

#### 4.4.2. The Role of Cu in Ankylosing Spondylitis

Cu, an essential trace element, has emerged as a potential contributor to the pathogenesis of AS through its involvement in a novel form of regulated cell death known as cuproptosis. This mitochondrial-dependent process may be linked to the immune dysregulation and inflammation observed in AS. Cuproptosis is a Cu-triggered cell death mechanism that disrupts mitochondrial respiration via aggregation of lipoylated proteins in the tricarboxylic acid (TCA) cycle. In AS, studies suggest abnormally elevated serum Cu levels, which may contribute to this pathway and promote cellular stress and tissue damage. Several studies that employed bioinformatics and machine learning approaches have identified CRGs that are differentially expressed in patients with AS, such as MTF1, ATP7A, and SLC31A1. These genes are linked to immune pathways, osteogenesis, and disease severity. Elevated expression of these genes in AS blood samples was validated through RT-qPCR and ELISA, which supports their relevance to disease processes [296,297]. Consensus clustering based on the expression of CRGs has identified distinct AS subtypes with differing levels of immune cell infiltration. One subtype shows higher levels of neutrophils, mast cells, and plasmacytoid dendritic cells, which indicates an immune landscape that is potentially shaped by Cu toxicity [296]. Despite the biological and transcriptomic evidence of Cu involvement in AS, an MR study found no significant genetic causal association between systemic Cu levels and AS risk. This suggests that, while Cu may influence disease progression, it may not be a primary etiological factor at the genetic level [298].

#### 4.4.3. The Role of Mn in Ankylosing Spondylitis

Mn is a vital trace element that serves as a cofactor for several antioxidant enzymes, most notably MnSOD. In AS, where oxidative stress is believed to contribute to chronic inflammation and tissue damage, Mn and MnSOD are of particular interest as potential modulators of disease activity. The most recent study identified on this topic, conducted by Yen et al. [299], assessed whether specific genetic variants of the MnSOD gene are associated with susceptibility to AS. The study analysed two polymorphisms—C1183T (Ala-9Val) and T5777C (Ile58Thr)—in 70 patients with AS and 93 healthy controls. The study found no significant differences in genotype, allele, or phenotype frequencies between patients with AS and controls. The T5777C polymorphism was not detected in either group. The C1183T polymorphism, which affects the mitochondrial targeting sequence of MnSOD and could theoretically alter the mitochondrial import efficiency, also showed no association with AS risk. These results suggest that MnSOD polymorphisms do not contribute to AS susceptibility in the studied population. Although genetic association was not established, the study emphasised the biological importance of MnSOD. Given the elevated oxidative stress reported in AS, reduced MnSOD activity could hypothetically impair mitochondrial defence mechanisms and enhance inflammation. Thus, Mn and MnSOD remain relevant in the context of the pathophysiology of AS, even in the absence of genetic predisposition [299]. 

#### 4.4.4. The Role of Zn in Ankylosing Spondylitis

Although Zn plays a critical role in immune regulation and inflammatory processes relevant to AS, current genetic evidence does not support a definitive causal relationship. MR analysis conducted by Sun et al. [298] showed a weak, suggestive association between genetically predicted Zn levels and increased AS risk based on IVW and weighted median methods. However, this association was not confirmed by more robust methods such as MR-Egger regression and CAUSE, which account for pleiotropy and statistical power. The minor effect size and inconsistency between analytical models suggest that the observed relationship may be a false-positive finding. Therefore, Zn cannot currently be considered a confirmed risk factor for AS, and further large-scale, well-powered studies are needed to clarify its role in the disease’s pathogenesis [298].

#### 4.4.5. The Role of Cd in Ankylosing Spondylitis

In the context of AS, Cd is hypothesised to contribute to the disease’s pathogenesis by enhancing oxidative stress, disrupting the cytokine balance, and promoting structural impairment. Mechanistically, Cd increases the production of ROS, induces mitochondrial dysfunction, and elevates oxidative DNA damage, as evidenced by increased 8-hydroxy-2’-deoxyguanosine (8-OHdG) levels. It also upregulates inflammatory mediators such as COX-2 and suppresses anti-inflammatory cytokines like IL-10, fostering a pro-inflammatory environment that is conducive to chronic autoimmune activation. In a clinical pilot study conducted in Lower Silesia, Poland, patients with inflammatory arthritis—including those with AS—were found to have significantly elevated blood Cd levels (Cd-B ≥ 0.65 µg/L). These levels were positively correlated with ESR, hs-CRP, COX-2, and 8-OHdG, and negatively correlated with hemoglobin and IL-10 concentrations. Notably, elevated Cd was associated with a 4.4-fold increased risk of arthritis (*p* = 0.038), a 6.1-fold higher risk of arthritis onset (*p* < 0.05), and significantly higher disease activity as measured by the BASDAI [289]. Complementary findings were observed in a population-based study using NHANES 2009–2010 data. Shiue [300] reported that higher urinary Cd concentrations were significantly associated with abnormal spinal mobility, a hallmark feature of AS. Specifically, individuals with elevated urinary Cd had a 2.17-fold-higher likelihood of having an abnormal occiput–wall distance, which suggests that Cd exposure may contribute to axial skeletal changes in the general population [300].

#### 4.4.6. The Role of Se in Ankylosing Spondylitis

Se is an essential trace element with antioxidant, anti-inflammatory, and immunomodulatory functions. Its role in autoimmune and inflammatory diseases, including in AS, has been of growing interest. A 2023 cross-sectional dietary study from Sweden found that patients with radiographic axial spondyloarthritis (r-axSpA) had significantly lower Se intake compared to matched healthy controls. The average intake among patients was 35.1 µg/day, compared to the 46.0 µg/day observed in controls, which was below the Nordic Nutrition Recommendations for both sexes. Additionally, fewer patients with AS met the recommended daily intake levels. This deficiency occurred in parallel with a lower intake of other anti-inflammatory nutrients such as omega-3 fatty acids, vitamins D, E, and K, and dietary fibre. The authors suggested that suboptimal Se intake could potentially contribute to sustained inflammation or impaired immune regulation in r-axSpA, although causality remains unproven [301]. However, a 2022 MR study using genome-wide data found no causal relationship between Se status and the risk of AS. Based on genetic variants associated with blood and toenail Se levels in European populations, none of the MR models (IVW, MR-Egger, weighted median, etc.) provided statistically significant evidence of a causal link. The inverse-variance weighted model yielded an OR of 0.999, and no horizontal pleiotropy or heterogeneity was observed, which suggests that these results are robust [298]. Current evidence suggests that Se deficiency is common in patients with AS and may be associated with increased inflammation due to the known biological functions of Se. However, genetic data do not support a causal role of the Se status in the onset of AS, which highlights the need for future interventional studies to determine whether Se supplementation can improve disease outcomes in Se-deficient patients.

### 4.5. The Role of Trace Elements in Systemic Lupus Erythematosus

Joint involvement is among the most frequent manifestations of SLE, occurring in up to 95% of patients and representing a significant burden on patients’ quality of life and functional status. Historically considered non-erosive, recent studies using advanced imaging techniques (ultrasound and MRI) have revealed a higher prevalence of chronic synovitis and erosions than previously recognised. Genetic polymorphisms (e.g., ITGAM, vitamin D receptor) have been associated with an increased risk of joint involvement in SLE. The presence of ACPA increases the risk of erosions, although the prevalence of erosions is lower than in RA. Joint involvement in SLE is heterogeneous and can vary from mild arthralgia to chronic erosive arthritis with potential functional impairment, including recognized clinical phenotypes such as non-deforming arthritis, which is characterized by migratory, symmetric pain and swelling that predominantly affects the small joints of the hands and wrists and is often accompanied by morning stiffness, Jaccoud’s arthropathy, which is characterized by reducible deformities such as ulnar deviation, swan neck, and Z-thumb, which may progress to fixed deformities over time, and Rhupus, an overlap syndrome with RA-like erosive arthritis and an autoantibody profile [302,303].

#### 4.5.1. The Role of Fe in Systemic Lupus Erythematosus

Dysregulated Fe metabolism plays a key role in the pathogenesis of SLE, particularly in joint involvement. Both Fe deficiency and Fe overload—alongside ferroptosis, a Fe-dependent form of regulated cell death—contribute to immune dysfunction, oxidative stress, and chronic inflammation (Table 5). Patients with SLE frequently exhibit functional Fe deficiency caused by chronic inflammation and IL-6-induced hepcidin overexpression. Hepcidin restricts Fe efflux from macrophages and enterocytes, decreasing the serum Fe availability. This impairs the mitochondrial function in T cells and monocytes, leading to reduced ATP production, increased ROS, and immune dysregulation that contributes to joint inflammation [304,305]. At the same time, excessive intracellular Fe accumulation can trigger ferroptosis, which is characterised by lipid peroxidation and oxidative stress. This process has been documented in macrophages, neutrophils, and T cells in SLE, is associated with the release of DAMPs (damage-associated molecular patterns), and perpetuates systemic and joint-specific inflammation [306,307,308,309]. This is particularly relevant in joint tissues, where activated immune cells are highly susceptible to mitochondrial damage and ferroptosis under inflammatory conditions. Studies have also shown that altered levels of Fe-handling proteins, such as serum transferrin and urinary ferritin, correlate with disease activity, renal involvement, and joint symptoms [310,311]. Genetic factors further influence the Fe metabolism in SLE. The IL-6 −174G>C polymorphism and HFE mutations (e.g., p.Cys282Tyr) have been associated with altered hepcidin expression and Fe availability, potentially modulating disease susceptibility and progression [304].

#### 4.5.2. The Role of Cu in Systemic Lupus Erythematosus

Cu is a vital trace element that is involved as a cofactor in numerous enzymes that regulate immune function and oxidative stress. In SLE, particularly in cases with joint involvement, elevated serum Cu levels have been consistently reported, which indicates its potential role in disease pathogenesis. A study by Tóth et al. [311] found significantly higher Cu levels in the serum of patients with SLE compared to healthy controls [311]. Notably, the Cu distribution among serum protein fractions was altered: in patients with SLE, the majority of Cu was bound to albumin, in contrast to healthy individuals, where ceruloplasmin was the primary carrier. This shift may reflect disrupted metal homeostasis under chronic inflammatory conditions. Chang et al. [310] additionally pointed to Cu’s possible involvement in cuproptosis—a newly identified form of programmed cell death that is Cu-dependent and linked to mitochondrial metabolism and immune gene regulation. Genes associated with cuproptosis (e.g., LIAS) were found to be differentially expressed in patients with SLE, which may influence the disease’s activity and prognosis [310].

#### 4.5.3. The Role of Zn in Systemic Lupus Erythematosus

Zn is a crucial trace element that is involved in numerous biological processes, particularly in immune regulation, the oxidative stress response, and gene expression. In patients with SLE, multiple studies report consistently decreased serum Zn levels compared to healthy controls, which suggests a link between Zn deficiency and autoimmune dysregulation. Elemental fractionation studies also show altered Zn–protein binding patterns in SLE. Normally, Zn is primarily bound to albumin in healthy serum, but in patients with SLE, Zn was found to be redistributed to ceruloplasmin, IgG, and transferrin fractions. This shift may reflect underlying inflammatory changes that affect the bioavailability and transport of Zn, possibly contributing to persistent joint inflammation. Taken together, the evidence suggests that Zn deficiency and the dysregulation of Zn-dependent proteins (e.g., Zn finger proteins) play a significant role in the immunopathology of SLE, particularly concerning joint involvement. These findings highlight the potential of Zn supplementation and Zn homeostasis modulation as therapeutic strategies for SLE [310,311].

#### 4.5.4. The Role of Cd in Systemic Lupus Erythematosus

Cd is a toxic heavy metal with a long biological half-life that accumulates in tissues, where it disrupts immune regulation and redox balance. Emerging evidence supports a link between Cd exposure and the development or exacerbation of SLE, particularly in cases that involve joint inflammation. Mechanistically, Cd promotes oxidative stress by displacing redox-active metal ions such as Zn^2+^ and Cu^+^ from key antioxidant enzymes (e.g., SOD, CAT, GPx), impairing their activity and leading to an overproduction of ROS. Excess ROS activate proinflammatory pathways such as the NF-κB and mitogen-activated protein kinase (MAPK) pathways, increases the production of cytokines including IL-6, IL-8, and IL-18, and induces endoplasmic reticulum stress—all of which are implicated in the pathogenesis of autoimmune and joint-related inflammation. Cd also alters cytokine expression profiles, suppresses the expression of MHC-II and CD40 on dendritic cells (inhibiting their maturation), and triggers polyclonal B-cell activation, which contributes to the loss of immune tolerance. These processes are driven by T-cell-derived cytokines and epigenetic modifications. A human case-control study revealed significantly higher blood Cd levels in women with SLE compared to healthy controls (0.059 vs. 0.017 mg/L), with a 4.45–6.68 times increased risk of developing SLE being observed in high-exposure groups. The Cd levels were also positively correlated with inflammatory markers and negatively correlated with the hemoglobin levels, which further suggests a connection to systemic and joint-specific inflammation [156].

#### 4.5.5. The Role of Hg in Systemic Lupus Erythematosus

Hg, a heavy metal that is widely present in dental amalgams and environmental sources, has been identified as an immune-modulating agent with the potential to trigger or exacerbate systemic autoimmune diseases, including SLE. Increasing evidence links Hg exposure to joint inflammation, particularly through delayed-type hypersensitivity (DTH) and autoantibody production. Hg can act both as an immunostimulant and immunosuppressant, depending on the form, dose, and host susceptibility. It activates type IV hypersensitivity reactions via memory T-cells, which leads to sustained inflammation and tissue damage—a hallmark of SLE joint involvement [312,313]. Hg binds covalently to thiol (-SH) groups in proteins, altering their structure and converting them into neoantigens, which elicit autoreactive T-cell responses. In animal models (e.g., Brown Norway rats), low-dose inorganic Hg exposure induces lupus-like symptoms, including immune complex deposition and skin and joint lesions. Additionally, Hg skews immune responses toward a Th2-dominant phenotype, promoting autoantibody formation (e.g., ANA) and reducing antiviral defences. It may also inhibit the function of regulatory T-cells, further compromising immune tolerance [313]. In a study by Stejskal et al. [312], 47% of patients with SLE showed a positive lymphocyte transformation test (LTT-MELISA®) response to Hg, compared to only 10% of healthy controls (*p* < 0.0001), which indicates a significantly higher rate of Hg hypersensitivity among patients with SLE. The Hg exposure in these cases was mostly attributed to dental amalgams and environmental contamination. Notably, some patients reported clinical improvement in joint symptoms after the removal of Hg-containing dental materials, which supports a potential causal or exacerbating role of Hg in the pathogenesis of lupus and joint inflammation [312].

#### 4.5.6. The Role of Ni in Systemic Lupus Erythematosus

Ni is one of the most prevalent environmental and occupational allergens and has been implicated in the pathogenesis of autoimmune diseases, including SLE with joint involvement. Its role is mainly associated with DTH reactions, which can provoke chronic immune activation and autoimmunity. Ni induces type IV hypersensitivity through activation of memory T cells. Upon exposure (e.g., via dental alloys, jewellery, food, or tobacco smoke), Ni can act as a hapten, binding to self-proteins like collagen and modifying them into neoantigens. These neoantigens are then recognised by the immune system, triggering autoreactive T-cell responses and promoting chronic inflammation—a process that is relevant to joint pathology in SLE. Experimental studies suggest that Ni exposure may promote autoantibody production, tissue fibrosis, and joint pain, with evidence from animal models showing the induction of scleroderma-like autoimmunity and connective tissue changes [313]. In a clinical study that involved 193 patients with connective tissue diseases (CTDs) including SLE, 52% tested positive for Ni hypersensitivity via the MELISA® lymphocyte transformation test, compared to 27% of healthy controls. Although the statistical significance was borderline (*p* = 0.056), this result indicates a strong trend toward increased Ni sensitivity in patients with CTD [312]. Additionally, 87% of the patients with CTD were hypersensitive to at least one metal, and 63% to two or more, with Ni being one of the most frequently identified allergens. Some patients experienced relief from joint pain, fatigue, and mucosal inflammation after the removal of Ni-containing dental materials, which suggests a possible causal or aggravating effect of Ni exposure. Ni may contribute to the initiation and perpetuation of autoimmune inflammation in patients with SLE through hapten-induced hypersensitivity and sustained T-cell activation. The evidence supports Ni as a relevant environmental trigger in joint-related lupus manifestations, particularly in sensitised individuals with dental or dermal exposure [312,313].

#### 4.5.7. The Role of Se in Systemic Lupus Erythematosus

Se is an essential trace element with antioxidant and immunomodulatory properties, and its deficiency has been associated with autoimmune diseases, including SLE. The study by Ye et al. [314] employed a two-sample MR design to investigate the causal relationship between genetically predicted Se levels and the risk of developing SLE. The analysis revealed a significant inverse association between Se levels and SLE risk. Specifically, for every natural log-transformed increase in genetically determined Se concentration, the risk of SLE decreased, which suggests a protective effect of Se on SLE development. Mechanistically, Se contributes to the immune system by regulating the activation, proliferation, and differentiation of immune cells, including T cells and natural killer (NK) cells. Se supplementation has been shown to reduce the number of activated T and NK cells and increase anti-inflammatory cytokine production. Given that oxidative stress and an imbalance between pro- and anti-inflammatory mediators are central to hte pathogenesis of SLE—especially in tissues such as joints—Se’s antioxidant properties may play a crucial role in mitigating joint inflammation. Animal studies further support these findings. In lupus-prone B6.Sle1b mice, Se supplementation impaired macrophage maturation, while in a NZB/NZW F1 lupus models, it enhanced survival and NK cell activity, supporting Se’s role in modulating inflammatory and immune responses in SLE. Taken together, these findings highlight Se’s potential role in modulating immune responses and oxidative stress, through which it contributes to reduced joint involvement in SLE. Further studies are needed to clarify the precise mechanisms and evaluate Se supplementation as a preventive or therapeutic strategy in patients with SLE with joint manifestations [314].

## 5. Conclusions

Proper joint function is an important factor that affects people’s quality of life. Individual trace elements such as Fe, Cu, Co, I, Mn, Zn, and Se are essential for proper joint function due to their involvement in enzyme formation and their effect on osteoblasts, osteoclasts, chondrocytes, and synovial membrane cells. However, excessive exposure to Fe, Co, Mn, and Zn can also be toxic to the cells that build joints. Cd, Hg, Pb, and Ni, on the other hand, have a toxic effect on joints. Co, Ag, Mn, Zn, Hg, and Se show potential roles in the treatment of joint diseases.

Elements can influence the development of joint diseases through various molecular pathways. In RA, high concentrations of Cu and Hg can lead to chronic inflammation, while Se, B, and Si can lead to a reduction in inflammation. Se is also responsible for GSH-Px activity. Zn deficiency can lead to increased pyroptosis in RA synoviocyte-like fibroblasts by disrupting the regulation of the (NLRP3)/caspase-1 pathway. Exposure to Cd increases the production of citrullinated peptides and proteins by increasing the calcium concentration and activating the enzyme peptidyl arginine deiminase. 

In OA, Fe accelerates chondrocyte apoptosis, which in turn leads to the expression of matrix-degrading enzymes such as MMP3 and MMP13 and to a reduction in type II collagen. Excess intracellular Fe can also cause synovial macrophages to polarise to the M1 type through phosphorylation of 4E-BP1 in the mTORC1-p70S6K/4E-BP1 pathway. This leads to increased production of pro-inflammatory factors such as IL-6 and TNF-α, which exacerbate cartilage damage. Higher Co concentrations induce pro-inflammatory responses, including increased expression of TNF-α and IL-6, M1 macrophage polarisation, and activation of the NF-κB signalling pathway. In addition, elevated Co^2+^ levels activate the PI3K/Akt pathway, upregulating the expression of the TLR4 membrane receptor, which further enhances the M1 inflammatory phenotype. Zn participates in the zinc-ZIP8-MTF1- HIF-2α pathway, is a cofactor of enzymes, and mediates the inflammatory response. Cd can inhibit or stimulate osteoblast proliferation, inhibit type I collagen synthesis, and modulate gene expression, including increasing the expression of BMP2 and FGF1. Cd also promotes matrix degradation by upregulating MMP1, MMP3, MMP9, and MMP13 and reducing the expression of cartilage matrix genes such as COL2A1 and ACAN. Cd increases the expression of the Bcl-2, BAX1, BAK, caspase 3, and caspase 9 proteins, which are key molecules in the intrinsic apoptosis pathway. Hg might induce epigenetic modifications of MMP and increase MMP-9. Chronic Pb exposure activates the NF-κB signalling pathway and also promotes osteoclast formation and disrupts the growth hormone/IGF-1 axis, which are key factors for skeletal growth and joint maintenance. Se activates the Nrf2 signalling pathway, an antioxidant defence regulator.

Excessive concentrations of Cu and Cd in PsA have a pro-inflammatory effect, while Se has an anti-inflammatory effect. Similarly, in PsA, Fe and Cd may amplify inflammatory responses. On the other hand, Se is an essential trace element with antioxidant, anti-inflammatory, and immunomodulatory properties. Cu may promote AS through cuproptosis.

In SLE, functional Fe deficiency caused by an increase in hepcidin levels induced by IL-6 leads to mitochondrial dysfunction and immune dysregulation. Zn deficiency and dysregulation of zinc-dependent proteins (e.g., zinc finger proteins) play an important role in the immunopathology of SLE. In turn, Cd increases ROS production, activates the NF-κB/MAPK pathway, impairs the antioxidant enzymes SOD, CAT, and GPx via Zn/Cu displacement, and promotes proinflammatory cytokines and autoimmunity. Se contributes to the immune system by regulating the activation, proliferation, and differentiation of immune cells, including T cells and natural killer (NK) cells.

In RA, the levels of Cu, Cd, Hg, Pb, and Ni were elevated, while the levels of Zn and Se were reduced. The researchers disagreed on the Mn levels, while the Fe levels were reduced in plasma and elevated in the synovial membrane. Patients with RA often suffer from Fe deficiency anaemia. The accumulation of Fe in the synovial membrane may indicate a link between its presence and the pathophysiology of chronic inflammation in RA. Elevated Cu levels may be associated with chronic inflammation. Co can generate ROS, leading to oxidative tissue damage. Mn and Zn compounds have potential applications in the treatment of RA. AgNPs also have therapeutic effects: anti-inflammatory and antioxidant effects. Cd increases the production of ACPA, RF, and ROS. Pb causes oxidative damage and has inflammatory and immunological properties. Ni disrupts antioxidant functions, depletes glutathione, increases ROS production, and causes inflammation. SeNPs show potential for use in the treatment of RA due to their antioxidant and anti-inflammatory properties. B and Si may inhibit chronic inflammation.

OA is characterised by elevated concentrations of Fe, Cu, Zn, Cd, and Pb, reduced levels of Mn and Se, and no changes in the concentration of Co, I, Hg, and Ni. Elevated Fe levels affect chondrocyte ferroptosis, lipid peroxidation, excessive expression of MMP, and increased release of pro-inflammatory cytokines. Cu affects chondrocytes, increasing ROS production, disturbances in the tricarboxylic acid cycle, maturation of Fe-sulphur clusters, and changes in the release of pro-inflammatory cytokines. At low concentrations, Co has an anti-inflammatory effect. At high concentrations, it can cause local and systemic metallosis. Co accumulates as a result of joint replacement. I participates in the formation of bone and cartilage. A deficiency of I in the diet is associated with the development of KBD. Mn is a cofactor of SOD2, which neutralises ROS, and a cofactor of GTF, which participates in the formation of cartilage components. Zn participates in the Zn-ZIP8-MTF1-HIF-2α pathway, is a cofactor for enzymes, and mediates the inflammatory response. Cd stimulates cytokine production, metalloproteinase activity, integrin dysregulation, and bone demineralisation, thereby accelerating cartilage degradation. The effect of Hg on OA has not been confirmed. Pb promotes increased expression of MMP, reduces ROS production, and disrupts normal collagen synthesis. Ni may exacerbate inflammation, oxidative stress, and cell apoptosis. Ni accumulates as a result of joint replacement. Se strengthens antioxidant defence, inhibits inflammation, and reduces the activity of cartilage-degrading enzymes. B supports osteogenesis, acts as an antioxidant, and reduces the level of pro-inflammatory cytokines.

PsA is characterised by elevated Fe, Cu, and Cd concentrations and reduced Zn and Se concentrations. Cu concentrations may be elevated or reduced. Elevated Fe concentrations were observed only in polyarticular disease. Cu may influence the pathogenesis of inflammation and be useful in monitoring disease activity and treatment progress. Elevated Cu concentrations were observed in both polyarticular and mono- or oligoarticular forms. Higher Cu concentrations were found in patients with positive inflammatory markers (ESR and CRP). Oral Zn sulphate may reduce clinical symptoms, but its role is not fully understood. It may influence the pathogenesis of inflammation. Cd levels were positively correlated with inflammatory markers such as ESR, CRP, and COX-2. Cd may influence the pathogenesis of inflammation and be useful in monitoring patients during treatment. Reduced levels of SELENOP and low GPx3 activity were also observed.

In AS, there is an increase in the Cu and Cd concentrations and a decrease in the Zn and Se concentrations. The Fe levels are elevated in granulocytes and platelets, while, systemically, they are dysregulated. No significant changes in Mn levels were found. Fe overload exacerbates oxidative stress and inflammation, and ferroptosis contributes to joint tissue damage. Cu can induce cuproptosis, which is associated with inflammation; it promotes oxidative stress and immune activation. Cd promotes oxidative stress, mitochondrial dysfunction, and the release of pro-inflammatory cytokines, contributing to joint inflammation and structural damage. Cd levels correlate positively with BASDAI, ESR, CRP, and spinal stiffness. Se deficiency may exacerbate oxidative stress and impair immune regulation in AS.

In turn, SLE is characterised by elevated concentrations of elements such as Cu, Cd, and Hg and reduced concentrations of Zn and Se. Fe levels are dysregulated, and intracellular overload occurs in immune cells. Functional Fe deficiency leads to mitochondrial dysfunction and immune dysregulation. Ferroptosis (from Fe overload) causes an increase in ROS and chronic inflammation in joint tissues. Elevated Cu is associated with inflammation, oxidative stress, and possibly cuproptosis. In patients with SLE, Cu moves from ceruloplasmin to albumin. Cd increases ROS production, activates NF-κB/MAPK, impairs antioxidant enzymes, and promotes pro-inflammatory cytokines and autoimmunity. Hg induces type IV hypersensitivity, alters the protein structure, and promotes autoreactive T-cell responses and arthritis. In the case of Ni, hypersensitivity is common. Ni acts as a hapten, binding to autoantibodies and inducing chronic inflammation, and thereby exacerbates arthritis in SLE. Low Se may worsen inflammation and joint damage in SLE. Genetically higher Se levels are associated with a reduced risk of SLE.

## Figures and Tables

**Table 2:** Summary of the role of trace elements in OA.

Trace Element	Level in Disease	Impact on the Disorder	Additional Information
Iron (Fe) [199,200,201,202,203,204,205,206,207,208,209,210]	Elevated	Fe affects ferroptosis of chondrocytes, lipid peroxidation, overexpression of metalloproteinases, and increased release of proinflammatory cytokines;	-
Copper (Cu) [97,211,212,213,214,215,216,217,218]	Elevated	Cu affects cuproptosis of chondrocytes, increased *reactive oxygen species* (ROS) production, disruption in the tricarboxylic acid cycle, maturation of Fe-sulfur clusters, and changes in pro-inflammatory cytokine release;	-
Cobalt (Co) [219,220,221,222,223,224,225,226]	Normal	In low concentrations, Co has anti-inflammatory effects; in high concentrations, Co can cause local and systemic metallosis;	Co accumulates as a result of joint replacement;
Iodine (I) [227,228,229,230]	Normal	I participates in the formation of bones and cartilage;	Dietary I deficiency is involved in the development of KBD;
Manganese (Mn) [58,118,231,232,233,234,235,236,237]	Decreased	Mn is a cofactor of superoxide dismutase 2 (SOD2), which neutralises ROS, and a cofactor of glycosyltransferase (GTF), which participates in the formation of cartilage components;	-
Zinc (Zn) [154,214,233,238,239,240,241,242,243]	Elevated	Zn participates in the Zrt- and Irt-like Protein 8 (zinc-ZIP8)-MTF1- hypoxia-inducible factor 2 alpha (HIF-2α) pathway, is a cofactor of enzymes, and mediates the inflammatory response;	-
Cadmium (Cd) [58,137,231,234,244,245,246,247,248,249]	Elevated	Cd stimulates cytokine production, metalloproteinase activity, integrin dysregulation, and bone demineralisation, therefore accelerating cartilage degradation;	Smoking cigarettes increases the concentration of Cd in the body;
Mercury (Hg) [41,231,250,251,252,253]	Normal	-	The effect of Hg on OA has not been confirmed;
Lead (Pb) [254,255,256,257,258,259,260,261]	Elevated	Pb promotes the upregulation of matrix metalloproteinases, reduces ROS production, and disrupts normal collagen synthesis;	-
Nickel (Ni) [156,262,263,264,265,266]	Normal	Ni may exacerbate inflammation, oxidative stress, and cellular apoptosis;	Ni accumulates as a result of joint replacement;
Selenium (Se) [49,54,58,231,238,241,267,268,269,270,271,272]	Decreased	Se enhances antioxidant defences, suppresses inflammation, and reduces the activity of cartilage-degrading enzymes;	There is research suggesting that increased dietary Se intake accelerates the progression of OA;
Boron (B) [56,58,59,273,274,275,276,277,278]	Decreased	B supports osteogenesis, acts as an antioxidant, reduces pro-inflammatory cytokines, and participates in hormonal balance;	-

Abbreviations: B—boron; Cd—cadmium; Co—cobalt; Cu—copper; Fe—iron; GTF—glycosyltransferase; Hg—mercury; HIF-2α—hypoxia-inducible factor 2 alpha; I—iodine; KBD—Kashin–Beck disease; Mn—manganese; Ni—nickel; Pb—lead; *ROS— reactive oxygen species;* Se—selenium; SOD2—superoxide dismutase 2; Zn—zinc; ZIP8—Zrt- and Irt-like protein 8.

**Table 4:** Summary of the role of trace elements in AS.

Trace Element	Level in Disease	Impact on the Disorder	Additional Information
Iron (Fe) [292,293,294,295]	Elevated intracellularly (in granulocytes, platelets); dysregulated systemically	Fe overload amplifies oxidative stress and inflammation; ferroptosis contributes to joint tissue damage; Fe affects macrophage polarisation, T-cell response, and cytokine production;	MR studies report conflicting results: one suggests a genetic association, another finds no causality; potential therapeutic targets include Fe chelators and ferroptosis inhibitors;
Copper (Cu) [296,297,298]	Elevated	Cu may induce *cuproptosis*—a novel mitochondrial-dependent cell death linked to inflammation; promotes oxidative stress and immune activation;	Cuproptosis-related genes (MTF1, ATP7A, SLC31A1) are upregulated in AS; no significant causal link found in MR study
Manganese (Mn) [299]	Not significantly altered	Mn is a cofactor for Mn superoxide dismutase (MnSOD), which detoxifies ROS in mitochondria; its deficiency could theoretically exacerbate oxidative stress, but no genetic link with AS was found;	A genetic study (Yen et al. [250]) found no association between MnSOD polymorphisms and AS susceptibility;
Zinc (Zn) [298]	Possibly decreased; data inconclusive;	Zn is crucial for immune regulation and inflammation, but MR analysis does not support a definitive causal role in AS;	Slight association in basic models, but not supported by MR-Egger or CAUSE, suggesting a false-positive;
Cadmium (Cd) [289,300]	Elevated (in environmentally exposed groups)	Cd promotes oxidative stress, mitochondrial dysfunction, and proinflammatory cytokine release ↑ *cyclooxygenase 2* (COX-2), ↓ interleukin (IL)-10), contributing to joint inflammation and structural damage;	Polish and NHANES studies: Cd positively correlates with BASDAI, ESR, CRP, and spine stiffness;
Selenium (Se) [298,301]	Decreased (dietary)	Se deficiency may enhance oxidative stress and impair immune regulation in AS, although MR data do not support causality;	Intake below Nordic recommendations in r-axSpA patients; further trials needed to assess effects of supplementation;

Abbrevitions: BASDAI—Bath Ankylosing Spondylitis Disease Activity Index; Cd—cadmium; COX-2—cyclooxygenase 2; CRP—C-reactive protein; Cu—copper; ESR—erythrocyte sedimentation rate; Fe—iron; IL—interleukine; Mn—manganese; MnSOD—manganese superoxide dismutase; MR—mendelian randomization; r-axSpA—radiographic axial spondyloarthritis; ROS—reactive oxygen species; Se—selenium; Zn—zinc.

**Table 5:** Summary of the role of trace elements in SLE.

Trace Element	Level in Disease	Impact on the Disorder	Additional Information
Iron (Fe) [304,305,306,307,308,309]	Dysregulated: often functionally deficient; intracellular overload in immune cells;	Functional Fe deficiency due to IL–6–driven hepcidin ↑ leads to mitochondrial dysfunction and immune dysregulation. Ferroptosis (from Fe overload) causes ROS ↑ and chronic inflammation in joint tissues;	Genetic factors (e.g., IL-6 −174G>C, HFE mutations) influence Fe metabolism and may affect SLE susceptibility;
Copper (Cu) [310,311]	Elevated	Elevated Cu is linked to inflammation, oxidative stress, and possibly cuproptosis, which disrupts mitochondrial function and immune gene expression;	In patients with SLE, Cu shifts from ceruloplasmin to albumin. Cuproptosis-related genes (e.g., *LIAS*) are differentially expressed;
Zinc (Zn) [310,311]	Decreased	Zn deficiency impairs immune function and promotes joint inflammation. Zn-binding to abnormal proteins in serum reflects inflammatory changes;	Redistribution of Zn from albumin to IgG, ceruloplasmin, and transferrin in SLE serum samples;
Cadmium (Cd) [156]	Elevated (in high exposure groups)	Cd increases ROS production, activates NF-κB/Mitogen-Activated Protein Kinase (MAPK), impairs antioxidant enzymes (via Zn/Cu displacement), and promotes proinflammatory cytokines and autoimmunity;	Blood Cd levels in women with SLE were 3–4x higher than controls; positively correlated with CRP, negatively with IL-10;
Mercury (Hg) [312,313]	Elevated (in sensitive individuals)	Hg induces type IV hypersensitivity, alters protein structure (neoantigens), and promotes autoreactive T-cell responses and joint inflammation;	LTT-MELISA tested positive in 47% of patients with SLE vs. 10% of controls; symptom improvement reported after amalgam removal;
Nickel (Ni) [312,313]	Hypersensitivity common	Ni acts as a hapten, binding to self-proteins and inducing chronic inflammation via Th1/Th2 imbalance; may aggravate joint inflammation in SLE;	MELISA: 52% of CTD patients (incl. SLE) Ni-sensitive vs. 27% of controls; symptom relief observed after Ni-containing dental material removal;
Selenium (Se) [314]	Decreased (dietary); genetically protective	Se regulates T/NK cell activation and reduces oxidative stress. Low Se may worsen inflammation and joint damage in SLE;	MR study: genetically higher Se levels are associated with reduced SLE risk. Se supplementation improved outcomes in lupus-prone mice;

Abbreviations: Cd—cadmium; CTD—connective tissue disease; Cu—copper; Fe—iron; Hg—mercury; MAPK—mitogen-activated protein kinase; MR—mendelian randomisation; Ni—nickel; ROS—reactive oxygen species; Se—selenium; Zn—zinc.

## Abbreviations

4-HNE	4-hydroxynonenal
8-OHdG	8-hydroxy-2’-deoxyguanosine
ACAN	aggrecan core protein
ACD	anemia of chronic disease
ACPA	anti-citrullinated peptide antibodies
ADAMTS	a disintegrin and metalloproteinase with thrombospondin motifs
AgNP	Ag nanoparticles
ALP	alkaline phosphatase
AS	ankylosing spondylitis
ASDAS	Ankylosing Spondylitis Disease Activity Score
BAK1	*Bcl-2 antagonist/killer 1*
BASDAI	Bath Ankylosing Spondylitis Disease Activity Index
BAX	bcl-2-associated X protein
BAX1	bcl-2 Associated X-protein 1
BMI	body mass index
*BMP2*	*bone morphogenetic protein 2*
BMSCs	bone marrow mesenchymal stem cells
B	boron
CDAI	clinical disease activity index
COX-2	cyclooxygenase-2
CRGs	cuproptosis-related genes
CTDs	connective tissue diseases
DAMPs	damage-associated molecular patterns
DAS28	disease activity score 28
DIO2	iodothyronine deiodinase-2
DMARDs	disease-modifying anti-rheumatic drugs
DMT1	divalent metal transporter 1
DTH	delayed-type hypersensitivity
ERAP1	endoplasmic reticulum aminopeptidase 1
ERK	extracellular signal-regulated kinase
ESR	erythrocyte sedimentation rate
*FGF-1*	*fibroblast growth factor 1*
FLSs	fibroblast-like synovial cells
GPx	glutathione peroxidase
GTF	glycosyltransferase
HIF-1α	hypoxia-inducible factor 1
HLA-B27	human leukocyte antigen B27
hs-CRP	high-sensitivity C-reactive protein
IA	inflammatory arthritis
IDA	iron deficiency anemia
IGF-1	insulin-like growth factor 1
IL	interleukin;
IL23R	interleukin-23 receptor
IRP1	iron regulatory protein 1
ISCA2	iron-sulfur cluster assembly 2
JAK	Janus kinase
KBD	Kashin–Beck disease
LNT-Se	lentinan-Se
MAPK	Mitogen-Activated Protein Kinase;
MCP-1	monocyte chemotactic protein-1
MMP	matrix metalloproteinases
MnSOD	manganese superoxide dismutase
MR	Mendelian randomization
MSCs	mesenchymal stem cells
MTs	metallothioneins
MTF-1	metal transcription factor-1
NAMPT	nicotinamide phosphoribosyltransferase
NFATc1	nuclear factor of activated T cells 1
NF-κB	nuclear factor kappa-light-chain-enhancer of activated B cells
NK	natural killer
Nrf2	nuclear factor erythroid 2-related factor 2
NSAIDs	non-steroidal anti-inflammatory drugs
OA	osteoarthritis
PsA	psoriatic arthritis
RA	rheumatoid arthritis
RANKL	receptor activator of nuclear factor kappa-Β ligand;
RANKL/RANK/OPG	receptor activator of nuclear factor-κB ligand/receptor activator of nuclear factor-κB/osteoprotegerin
r-axSpA	radiographic axial spondyloarthritis
RF	rheumatoid factor
ROS	reactive oxygen species
RUNX2	runt-related transcription factor 2
SDAI	simple disease activity index
SELENOP	selenoprotein P
SeMetFa NPs	selenium-methionine-folic acid nanoparticles
SeNPs	selenium nanoparticles
Si	silicon
SIIS	silicone implant incompatibility syndrome
SII	systemic immune-inflammation index
SLE	systemic lupus erythematosus
SOD	superoxide dismutase
sTfR	soluble transferrin receptor
T3	triiodothyronine
T4	thyroxine
TCA	tricarboxylic acid
TGF-β	transforming growth factor β
TH	thyroid hormones;
TIBC	total iron binding capacity
TJR	total joint replacement
TR1	thioredoxin reductase 1
TRAP	tartrate-resistant acid phosphatase
TSAT	transferrin saturation
Wnt	wingless-related integration site
ZIP8	Zrt- and Irt-like Protein 8
ZNF345	zinc finger protein 345
NLRP3	NLR family pyrin domain containing 3
PI3K/Akt	phosphoinositide 3-kinase/Protein kinase B
Zip14	zinc-regulated transporter, iron-regulated transporter-like protein 14

## References

[1] Leafblad N., Mizels J., Tashjian R., Chalmers P. (2023). Adhesive Capsulitis. Phys. Med. Rehabil. Clin. N. Am..

[2] Cardoneanu A., Macovei L.A., Burlui A.M., Mihai I.R., Bratoiu I., Rezus I.I., Richter P., Tamba B.I., Rezus E. (2023). Temporomandibular Joint Osteoarthritis: Pathogenic Mechanisms Involving the Cartilage and Subchondral Bone, and Potential Therapeutic Strategies for Joint Regeneration. Int. J. Mol. Sci..

[3] Huang H., Lin Y., Jiang Y., Yao Q., Chen R., Zhao Y.Z., Kou L. (2023). Recombinant protein drugs-based intra articular drug delivery systems for osteoarthritis therapy. Eur. J. Pharm. Biopharm..

[4] Hwang M.C., Ridley L., Reveille J.D. (2021). Ankylosing spondylitis risk factors: A systematic literature review. Clin. Rheumatol..

[5] Semenistaja S., Skuja S., Kadisa A., Groma V. (2023). Healthy and Osteoarthritis-Affected Joints Facing the Cellular Crosstalk. Int. J. Mol. Sci..

[6] Coaccioli S., Sarzi-Puttini P., Zis P., Rinonapoli G., Varrassi G. (2022). Osteoarthritis: New Insight on Its Pathophysiology. J. Clin. Med..

[7] Lubrano E., Perrotta F.M. (2023). Sex-related differences in psoriatic arthritis. Lancet Rheumatol..

[8] Zhao T., Wei Y., Zhu Y., Xie Z., Hai Q., Li Z., Qin D. (2022). Gut microbiota and rheumatoid arthritis: From pathogenesis to novel therapeutic opportunities. Front. Immunol..

[9] Gottlieb A., Merola J.F. (2020). Psoriatic arthritis for dermatologists. J. Dermatolog. Treat..

[10] Radu A.F., Bungau S.G. (2021). Management of rheumatoid arthritis: An overview. Cells.

[11] Mertz W. (1981). The essential trace elements. Science.

[12] Zoroddu M.A., Aaseth J., Crisponi G., Medici S., Peana M., Nurchi V.M. (2019). The essential metals for humans: A brief overview. J. Inorg. Biochem..

[13] Jan A.T., Azam M., Siddiqui K., Ali A., Choi I., Haq Q.M.R. (2015). Heavy metals and human health: Mechanistic insight into toxicity and counter defense system of antioxidants. Int. J. Mol. Sci..

[14] Perrelli M., Wu R., Liu D.J., Lucchini R.G., Del Bosque-Plata L., Vergare M.J., Akhter M.P., Ott J., Gragnoli C. (2022). Heavy metals as risk factors for human diseases—A Bayesian network approach. Eur. Rev. Med. Pharmacol. Sci..

[15] Jannetto P.J., Cowl C.T. (2023). Elementary Overview of Heavy Metals. Clin. Chem..

[16] Karim A., Bajbouj K., Qaisar R., Hall A.C., Hamad M. (2022). The role of disrupted iron homeostasis in the development and progression of arthropathy. J. Orthop. Res..

[17] He Q., Yang J., Pan Z., Zhang G., Chen B., Li S., Xiao J., Tan F., Wang Z., Chen P. (2023). Biochanin A protects against iron overload associated knee osteoarthritis via regulating iron levels and NRF2/System xc-/GPX4 axis. Biomed. Pharmacother..

[18] Sun K., Guo Z., Hou L., Xu J., Du T., Xu T., Guo F. (2021). Iron homeostasis in arthropathies: From pathogenesis to therapeutic potential. Ageing Res. Rev..

[19] Pang N., Ding M., Yang H., Zhong Q., Zheng L., Luo D., Yao Y. (2024). Iron overload causes macrophages to produce a pro-inflammatory phenotype in the synovium of hemophiliac arthritis via the acetyl-p53 pathway. Haemophilia.

[20] Griffith D.P., Liff D.A., Ziegler T.R., Esper G.J., Winton E.F. (2009). Acquired copper deficiency: A potentially serious and preventable complication following gastric bypass surgery. Obesity.

[21] Liu Y., Zhu J., Xu L., Wang B., Lin W., Luo Y. (2022). Copper regulation of immune response and potential implications for treating orthopedic disorders. Front. Mol. Biosci..

[22] Leyssens L., Vinck B., Van Der Straeten C., Wuyts F., Maes L. (2017). Cobalt toxicity in humans—A review of the potential sources and systemic health effects. Toxicology.

[23] Queally J.M., Devitt B.M., Butler J.S., Malizia A.P., Murray D., Doran P.P., O’Byrne J.M. (2009). Cobalt ions induce chemokine secretion in primary human osteoblasts. J. Orthop. Res..

[24] Drynda A., Drynda S., Kekow J., Lohmann C.H., Bertrand J. (2018). Differential effect of cobalt and chromium ions as well as cocr particles on the expression of osteogenic markers and osteoblast function. Int. J. Mol. Sci..

[25] McCarthy E.M., Floyd H., Addison O., Zhang Z.J., Oppenheimer P.G., Grover L.M. (2018). Influence of Cobalt Ions on Collagen Gel Formation and Their Interaction with Osteoblasts. ACS Omega.

[26] Eltit F., Noble J., Sharma M., Benam N., Haegert A., Bell R.H., Simon F., Duncan C.P., Garbuz D.S., Greidanus N.V. (2021). Cobalt ions induce metabolic stress in synovial fibroblasts and secretion of cytokines/chemokines that may be diagnostic markers for adverse local tissue reactions to hip implants. Acta Biomater..

[27] Gunnarsdottir I., Dahl L. (2012). Iodine intake in human nutrition: A systematic literature review. Food Nutr. Res..

[28] Kim H.Y., Mohan S. (2013). Role and Mechanisms of Actions of Thyroid Hormone on the Skeletal Development. Bone Res..

[29] Horning K.J., Caito S.W., Tipps K.G., Bowman A.B., Aschner M. (2015). Manganese is Essential for Neuronal Health. Annu. Rev. Nutr..

[30] Taskozhina G., Batyrova G., Umarova G., Issanguzhina Z., Kereyeva N. (2024). The Manganese–Bone Connection: Investigating the Role of Manganese in Bone Health. J. Clin. Med..

[31] O’Connor J.P., Kanjilal D., Teitelbaum M., Lin S.S., Cottrell J.A. (2020). Zinc as a therapeutic agent in bone regeneration. Materials.

[32] Yin I.X., Zhang J., Zhao I.S., Mei M.L., Li Q., Chu C.H. (2020). The antibacterial mechanism of silver nanoparticles and its application in dentistry. Int. J. Nanomed..

[33] Jeyaraman M., Jeyaraman N., Nallakumarasamy A., Potty A.G., Gupta A., Iyengar K.P., Jain V.K. (2023). Silver nanoparticle technology in orthopaedic infections. World J. Orthop..

[34] Aurore V., Caldana F., Blanchard M., Kharoubi Hess S., Lannes N., Mantel P.Y., Filgueira L., Walch M. (2018). Silver-nanoparticles increase bactericidal activity and radical oxygen responses against bacterial pathogens in human osteoclasts. Nanomed. Nanotechnol. Biol. Med..

[35] Engström A., Skerving S., Lidfeldt J., Burgaz A., Lundh T., Samsioe G., Vahter M., Åkesson A. (2009). Cadmium-induced bone effect is not mediated via low serum 1,25-dihydroxy vitamin D. Environ. Res..

[36] Ma Y., Ran D., Shi X., Zhao H., Liu Z. (2021). Cadmium toxicity: A role in bone cell function and teeth development. Sci. Total Environ..

[37] Wang M., Liu J., Zhu G., Chen X. (2022). Low levels of cadmium exposure affect bone by inhibiting Lgr4 expression in osteoblasts and osteoclasts. J. Trace Elem. Med. Biol..

[38] Fernández-Torres J., Plata-Rodríguez R., Zamudio-Cuevas Y., Martínez-Nava G.A., Landa-Solís C., Mendoza Soto L., Olivos-Meza A., Suárez-Ahedo C., Barbier O.C., Narváez-Morales J. (2020). Effect of cadmium on the viability on monolayer cultures of synoviocytes, chondrocytes, and Hoffa: A preliminary study. Toxicol. Ind. Health.

[39] Rodríguez J., Mandalunis P.M. (2018). A review of metal exposure and its effects on bone health. J. Toxicol..

[40] Vianna A.D.S., Matos E.P.D., Jesus I.M.D., Asmus C.I.R.F., Câmara V.D.M. (2019). Human exposure to mercury and its hematological effects: A systematic revie. Cad. Saude Publica.

[41] Pamphlett R., Jew S.K. (2019). Mercury is taken up selectively by cells involved in joint, bone, and connective tissue disorders. Front. Med..

[42] Charkiewicz A.E., Backstrand J.R. (2020). Lead toxicity and pollution in Poland. Int. J. Environ. Res. Public Health.

[43] Li G., Xiong C., Xu W., Mei R., Cheng T., Yu X. (2021). Factors Affecting the Aluminum, Arsenic, Cadmium and Lead Concentrations in the Knee Joint Structures. Front. Public Health.

[44] Beier E.E., Sheu T.J., Dang D., Holz J.D., Ubayawardena R., Babij P., Puzas J.E. (2015). Heavy metal ion regulation of gene expression: Mechanisms by which lead inhibits osteoblastic bone-forming activity through modulation of the Wnt/β-catenin signaling pathway. J. Biol. Chem..

[45] Skalny A.V., Aschner M., Zhang F., Guo X., Buha Djordevic A., Sotnikova T.I., Korobeinikova T.V., Domingo J.L., Farsky S.H.P., Tinkov A.A. (2024). Molecular mechanisms of environmental pollutant-induced cartilage damage: From developmental disorders to osteoarthritis. Arch. Toxicol..

[46] Denkhaus E., Salnikow K. (2002). Nickel essentiality, toxicity, and carcinogenicity. Crit. Rev. Oncol. Hematol..

[47] Kanaji A., Orhue V., Caicedo M.S., Virdi A.S., Sumner D.R., Hallab N.J., Yoshiaki T., Sena K. (2014). Cytotoxic effects of cobalt and nickel ions on osteocytes in vitro. J. Orthop. Surg. Res..

[48] Zhang F., Li X., Wei Y. (2023). Selenium and Selenoproteins in Health. Biomolecules.

[49] Deng H., Liu H., Yang Z., Bao M., Lin X., Han J., Qu C. (2022). Progress of Selenium Deficiency in the Pathogenesis of Arthropathies and Selenium Supplement for Their Treatment. Biol. Trace Elem. Res..

[50] Honkanen V.E.A. (1991). The Factors Affecting Plasma Glutathione Peroxidase And Selenium In Rheumatoid Arthritis: A multiple linear regression analysis. Scand. J. Rheumatol..

[51] Zeng H., Cao J.J., Combs G.F. (2013). Selenium in bone health: Roles in antioxidant protection and cell proliferation. Nutrients.

[52] Yang T., Lee S.Y., Park K.C., Park S.H., Chung J., Lee S. (2022). The Effects of Selenium on Bone Health: From Element to Therapeutics. Molecules.

[53] Cheng A.W.M., Bolognesi M., Kraus V.B. (2012). DIO2 modifies inflammatory responses in chondrocytes. Osteoarthr. Cartil..

[54] Cheng H.L., Yen C.C., Huang L.W., Hu Y.C., Huang T.C., Hsieh B.S., Chang K.L. (2024). Selenium Lessens Osteoarthritis by Protecting Articular Chondrocytes from Oxidative Damage through Nrf2 and NF-κB Pathways. Int. J. Mol. Sci..

[55] Rondanelli M., Faliva M.A., Peroni G., Infantino V., Gasparri C., Iannello G., Perna S., Riva A., Petrangolini G., Tartara A. (2020). Pivotal role of boron supplementation on bone health: A narrative review. J. Trace Elem. Med. Biol..

[56] Newnham R.E. (1994). Essentiality of boron for healthy bones and joints. Environ. Health Perspect..

[57] Hunt C.D. (1994). The biochemical effects of physiologic amounts of dietary boron in animal nutrition models. Environ. Health Perspect..

[58] Li G., Cheng T., Yu X. (2021). The Impact of Trace Elements on Osteoarthritis. Front. Med..

[59] Korkmaz M., Turkmen R., Demirel H.H., Saritas Z.K. (2019). Effect of Boron on the Repair of Osteochondral Defect and Oxidative Stress in Rats: An Experimental Study. Biol. Trace Elem. Res..

[60] Nielsen F.H. (2014). Update on human health effects of boron. J. Trace Elem. Med. Biol..

[61] Mladenović Ž., Johansson A., Willman B., Shahabi K., Björn E., Ransjö M. (2014). Soluble silica inhibits osteoclast formation and bone resorption in vitro. Acta Biomater..

[62] Kim E.J., Bu S.Y., Sung M.K., Choi M.K. (2013). Effects of silicon on osteoblast activity and bone mineralization of MC3T3-E1 cells. Biol. Trace Elem. Res..

[63] Beck G.R., Ha S.W., Camalier C.E., Yamaguchi M., Li Y., Lee J.K., Weitzmann M.N. (2012). Bioactive silica-based nanoparticles stimulate bone-forming osteoblasts, suppress bone-resorbing osteoclasts, and enhance bone mineral density in vivo. Nanomed. Nanotechnol. Biol. Med..

[64] Magnusson C., Ransjö M. (2024). Orthosilicic acid inhibits human osteoclast differentiation and bone resorption. PLoS ONE.

[65] Schröder H.C., Wang X.H., Wiens M., Diehl-Seifert B., Kropf K., Schloßmacher U., Müller W.E.G. (2012). Silicate modulates the cross-talk between osteoblasts (SaOS-2) and osteoclasts (RAW 264.7 cells): Inhibition of osteoclast growth and differentiation. J. Cell. Biochem..

[66] Carlisle E.M. (1976). In vivo requirement for silicon in articular cartilage and connective tissue formation in the chick. J. Nutr..

[67] Carlisle E.M. (2007). Silicon as an essential trace element in animal nutrition. Silicon Biochem..

[68] Kitala K., Tanski D., Godlewski J., Krajewska-Włodarczyk M., Gromadziński L., Majewski M. (2023). Copper and Zinc Particles as Regulators of Cardiovascular System Function—A Review. Nutrients.

[69] Doguer C., Ha J.H., Collins J.F. (2018). Intersection of Iron and Copper Metabolism in the Mammalian Intestine and Liver. Compr. Physiol..

[70] Bodiga S., Krishnapillai M.N. (2007). Concurrent repletion of iron and zinc reduces intestinal oxidative damage in iron- and zinc-deficient rats. World J. Gastroenterol..

[71] Olivares M., Pizarro F., Ruz M., De Romaña D.L. (2012). Acute inhibition of iron bioavailability by zinc: Studies in humans. BioMetals.

[72] Lönnerdal B. (2000). Dietary factors influencing zinc absorption. J. Nutr..

[73] Sandstrom B., Davidsson L., Cederblad A., Lonnerdal B. (1985). Oral iron, dietary ligands and zinc absorption. J. Nutr..

[74] Whittaker P. (1998). Iron and zinc interactions in humans. Am. J. Clin. Nutr..

[75] Donangelo C.M., Woodhouse L.R., King S.M., Viteri F.E., King J.C. (2002). Supplemental zinc lowers measures of iron status in young women with low iron reserves. J. Nutr..

[76] Yanagisawa H., Miyakoshi Y., Kobayashi K., Sakae K., Kawasaki I., Suzuki Y., Tamura J. (2009). Long-term intake of a high zinc diet causes iron deficiency anemia accompanied by reticulocytosis and extra-medullary erythropoiesis. Toxicol. Lett..

[77] Chua A.C.G., Morgan E.H. (1996). Effects of iron deficiency and iron overload on manganese uptake and deposition in the brain and other organs of the rat. Biol. Trace Elem. Res..

[78] Mehri A. (2020). Trace Elements in Human Nutrition (II)—An Update. Int. J. Prev. Med..

[79] Turan E., Karaaslan O. (2020). The Relationship between Iodine and Selenium Levels with Anxiety and Depression in Patients with Euthyroid Nodular Goiter. Oman Med. J..

[80] Ćwiertnia A., Kozłowski M., Cymbaluk-Płoska A. (2022). The Role of Iron and Cobalt in Gynecological Diseases. Cells.

[81] Czarnek K., Terpilowska S., Siwicki A.K. (2015). Selected aspects of the action of cobalt ions in the human body. Cent. J. Immunol..

[82] Nemec A.A., Leikauf G.D., Pitt B.R., Wasserloos K.J., Barchowsky A. (2008). Nickel Mobilizes Intracellular Zinc to Induce Metallothionein in Human Airway Epithelial Cells. Am. J. Respir. Cell Mol. Biol..

[83] Spiller H.A. (2018). Rethinking mercury: The role of selenium in the pathophysiology of mercury toxicity. Clin. Toxicol..

[84] Lansdown A.B.G. (2010). A Pharmacological and Toxicological Profile of Silver as an Antimicrobial Agent in Medical Devices. Adv. Pharmacol. Sci..

[85] Biţă A., Scorei I.R., Bălşeanu T.A., Rău G., Ciocîlteu M.V., Mogoşanu G.D. (2023). Zinc-Boron Complex-Based Dietary Supplements for Longevity and Healthy Life. Curr. Health Sci. J..

[86] Ghio A.J., Tong H., Soukup J.M., Dailey L.A., Cheng W.Y., Samet J.M., Kesic M.J., Bromberg P.A., Turi J.L., Upadhyay D. (2013). Sequestration of mitochondrial iron by silica particle initiates a biological effect. Am. J. Physiol.-Lung Cell. Mol. Physiol..

[87] Mercadante C.J., Herrera C., Pettiglio M.A., Foster M.L., Johnson L.C., Dorman D.C., Bartnikas T.B. (2016). The effect of high dose oral manganese exposure on copper, iron and zinc levels in rats. Biometals.

[88] Rozenberg J.M., Kamynina M., Sorokin M., Zolotovskaia M., Koroleva E., Kremenchutckaya K., Gudkov A., Buzdin A., Borisov N. (2022). The Role of the Metabolism of Zinc and Manganese Ions in Human Cancerogenesis. Biomedicines.

[89] Firestein G.S., McInnes I.B. (2017). Immunopathogenesis of Rheumatoid Arthritis. Immunity.

[90] Venetsanopoulou A.I., Alamanos Y., Voulgari P.V., Drosos A.A. (2023). Epidemiology and Risk Factors for Rheumatoid Arthritis Development. Mediterr. J. Rheumatol..

[91] Smolen J.S., Aletaha D., Barton A., Burmester G.R., Emery P., Firestein G.S., Kavanaugh A., McInnes I.B., Solomon D.H., Strand V. (2018). Rheumatoid arthritis. Nat. Rev. Dis. Prim..

[92] Sparks J.A. (2019). In the Clinic® rheumatoid arthritis. Ann. Intern. Med..

[93] Bullock J., Rizvi S.A.A., Saleh A.M., Ahmed S.S., Do D.P., Ansari R.A., Ahmed J. (2019). Rheumatoid arthritis: A brief overview of the treatment. Med. Princ. Pract..

[94] Wu D., Luo Y., Li T., Zhao X., Lv T., Fang G., Ou P., Li H., Luo X., Huang A. (2022). Systemic complications of rheumatoid arthritis: Focus on pathogenesis and treatment. Front. Immunol..

[95] Smolen J.S., Aletaha D., McInnes I.B. (2016). Rheumatoid arthritis. Lancet.

[96] Jang S., Kwon E.J., Lee J.J. (2022). Rheumatoid Arthritis: Pathogenic Roles of Diverse Immune Cells. Int. J. Mol. Sci..

[97] Yazar M., Sarban S., Kocyigit A., Isikan U.E. (2005). Synovial Fluid and Plasma Selenium, Copper, Zinc, and Iron Concentrations in Patients with Rheumatoid Arthritis and Osteoarthritis. Biol. Trace Elem. Res..

[98] Wang H., Zhang R., Shen J., Jin Y., Chang C., Hong M., Guo S., He D. (2023). Circulating Level of Blood Iron and Copper Associated with Inflammation and Disease Activity of Rheumatoid Arthritis. Biol. Trace Elem. Res..

[99] Khadim R.M., Al-Fartusie F.S. (2023). Evaluation of some trace elements and antioxidants in sera of patients with rheumatoid arthritis: A case–control study. Clin. Rheumatol..

[100] Khalaf W., Al-Rubaie H.A., Shihab S. (2019). Studying anemia of chronic disease and iron deficiency in patients with rheumatoid arthritis by iron status and circulating hepcidin. Hematol. Rep..

[101] Majhi T. (2010). Iron deficiency in rheumatoid arthritic patients especially with in the middle age. Int. J. Syst. Biol..

[102] Stefanova K.I., Delcheva G.T., Maneva A.I., Batalov A.Z., Geneva-Popova M.G., Karalilova R.V., Simitchiev K.K. (2018). Pathobiochemical Mechanisms Relating Iron Homeostasis with Parameters of Inflammatory Activity and Autoimmune Disorders in Rheumatoid Arthritis. Folia Med..

[103] Fritz P., Saal J.G., Wicherek C., Kiinig A., Laschner W., Rautenstrauch H. (1996). Quantitative photometrical assessment of iron deposits in synovial membranes in different joint diseases. Rheumatol. Int..

[104] Zhao T., Yang Q., Xi Y., Xie Z., Shen J., Li Z., Li Z., Qin D. (2022). Ferroptosis in Rheumatoid Arthritis: A Potential Therapeutic Strategy. Front. Immunol..

[105] Liu Y., Luo X., Chen Y., Dang J., Zeng D., Guo X., Weng W., Zhao J., Shi X., Chen J. (2024). Heterogeneous ferroptosis susceptibility of macrophages caused by focal iron overload exacerbates rheumatoid arthritis. Redox Biol..

[106] Vera E., Vallvé J.C., Linares V., Paredes S., Ibarretxe D., Bellés M. (2024). Serum Levels of Trace Elements (Magnesium, Iron, Zinc, Selenium, and Strontium) are Differentially Associated with Surrogate Markers of Cardiovascular Disease Risk in Patients with Rheumatoid Arthritis. Biol. Trace Elem. Res..

[107] Xin L., Yang X., Cai G., Fan D., Xia Q., Liu L., Hu Y., Ding N., Xu S., Wang L. (2015). Serum Levels of Copper and Zinc in Patients with Rheumatoid Arthritis: A Meta-analysis. Biol. Trace Elem. Res..

[108] Sahebari M., Ayati R., Mirzaei H., Sahebkar A., Hejazi S., Saghafi M., Saadati N., Ferns G.A., Ghayour-Mobarhan M. (2016). Serum Trace Element Concentrations in Rheumatoid Arthritis. Biol. Trace Elem. Res..

[109] Strecker D., Mierzecki A., Radomska K. (2013). Copper levels in patients with rheumatoid arthritis. Ann. Agric. Environ. Med..

[110] Önal S., Nazıroğlu M., Çolak M., Bulut V., Flores-Arce M.F. (2011). Effects of different medical treatments on serum copper, selenium and zinc levels in patients with rheumatoid arthritis. Biol. Trace Elem. Res..

[111] Ullah Z., Ullah M.I., Hussain S., Kaul H., Lone K.P. (2017). Determination of Serum Trace Elements (Zn, Cu, and Fe) in Pakistani Patients with Rheumatoid Arthritis. Biol. Trace Elem. Res..

[112] Han J., Luo J., Wang C., Kapilevich L., Zhang X. (2024). Roles and mechanisms of copper homeostasis and cuproptosis in osteoarticular diseases. Biomed. Pharmacother..

[113] Irfan S., Rani A., Riaz N., Arshad M., Kashif Nawaz S. (2017). Comparative Evaluation of Heavy Metals in Patients with Rheumatoid Arthritis and Healthy Control in Pakistani Population. Iran. J. Public Health.

[114] El-Sharkawy R.G., Taha R.H., Ghanem H.B. (2020). Immobilization of novel inorganic nano-complexes onto MWCNT nanomaterials as a novel adsorbent and anti-inflammatory therapy in an induced model of rheumatoid arthritis. Nanotechnology.

[115] Hällgren R., Svenson K., Johansson E., Lindh U. (1985). Elevated granulocyte manganese in rheumatoid arthritis and other connective tissue diseases. J. Rheumatol..

[116] Cotzias G.C., Papavasiliou P.S., Hughes E.R., Tang L., Borg D.C. (1968). Slow turnover of manganese in active rheumatoid arthritis accelerated by prednisone. J. Clin. Investig..

[117] Sarban S., Isikan U.E., Kocabey Y., Kocyigit A. (2007). Relationship between synovial fluid and plasma manganese, arginase, and nitric oxide in patients with rheumatoid arthritis. Biol. Trace Elem. Res..

[118] Guan T., Wu Z., Xu C., Su G. (2023). The association of trace elements with arthritis in US adults: NHANES 2013–2016. J. Trace Elem. Med. Biol..

[119] Joo S.H., Lee J., Hutchinson D., Song Y.W. (2019). Prevalence of rheumatoid arthritis in relation to serum cadmium concentrations: Cross-sectional study using Korean National Health and Nutrition Examination Survey (KNHANES) data. BMJ Open.

[120] Das D.C., Jahan I., Uddin M.G., Hossain M.M., Chowdhury M.A.Z., Fardous Z., Rahman M.M., Kabir A.K.M.H., Deb S.R., Siddique M.A.B. (2021). Serum CRP, MDA, Vitamin C, and Trace Elements in Bangladeshi Patients with Rheumatoid Arthritis. Biol. Trace Elem. Res..

[121] Afridi H.I., Kazi T.G., Brabazon D., Naher S. (2011). Association between essential trace and toxic elements in scalp hair samples of smokers rheumatoid arthritis subjects. Sci. Total Environ..

[122] Pasquier C., Mach P.S., Raichvarg D., Sarfati G., Amor B., Delbarre F. (1984). Manganese-containing superoxide-dismutase deficiency in polymorphonuclear leukocytes of adults with rheumatoid arthritis. Inflammation.

[123] Parizada B., Werber M.M., Nimrod A. (1991). Protective effects of human recombinant mnsod in adjuvant arthritis and bleomycin-induced lung fibrosis. Free Radic. Res..

[124] Salvemini D., Mazzon E., Dugo L., Serraino I., De Sarro A., Caputi A.P., Cuzzocrea S. (2001). Amelioration of Joint Disease in a Rat Model of Collagen-Induced Arthritis by M40403, a Superoxide Dismutase Mimetic. Arthritis Rheum. Off. J. Am. Coll. Rheumatol..

[125] Di Cesare Mannelli L., Bani D., Bencini A., Brandi M.L., Calosi L., Cantore M., Carossino A.M., Ghelardini C., Valtancoli B., Failli P. (2013). Therapeutic effects of the superoxide dismutase mimetic compound MnII MeO2A on experimental articular pain in rats. Mediat. Inflamm..

[126] Jia J., Liu M., Yang H., Li X.F., Liu S., Li K., Zhang J., Zhao X. (2025). Manganese Dioxide-Based pH-Responsive Multifunctional Nanoparticles Deliver Methotrexate for Targeted Rheumatoid Arthritis Treatment. Biomater. Res..

[127] Chen X., Zhang L., Zeng H., Meng W., Liu G., Zhang W., Zhao P., Zhang Q., Chen M., Chen J. (2023). Manganese-Based Immunomodulatory Nanocomposite with Catalase-Like Activity and Microwave-Enhanced ROS Elimination Ability for Efficient Rheumatoid Arthritis Therapy. Small.

[128] Xia T., Zhu Y., Li K., Hao K., Chai Y., Jiang H., Lou C., Yu J., Yang W., Wang J. (2024). Microneedles loaded with cerium-manganese oxide nanoparticles for targeting macrophages in the treatment of rheumatoid arthritis. J. Nanobiotechnol..

[129] Li H., Jin X., Chu B., Zhang K., Qin X., Pan S., Zhao Y., Shi H., Zhang J., Wang H. (2025). Inflammation Targeting and Responsive Multifunctional Drug-Delivery Nanoplatforms for Combined Therapy of Rheumatoid Arthritis. Small.

[130] Yang B., Yao H., Yang J., Chen C., Guo Y., Fu H., Shi J. (2022). In Situ Synthesis of Natural Antioxidase Mimics for Catalytic Anti-Inflammatory Treatments: Rheumatoid Arthritis as an Example. J. Am. Chem. Soc..

[131] Afridi H.I., Talpur F.N., Kazi T.G., Brabazon D. (2015). Estimation of toxic elements in the samples of different cigarettes and their effect on the essential elemental status in the biological samples of Irish smoker rheumatoid arthritis consumers. Environ. Monit. Assess..

[132] Duarte G.B.S., Callou K.R.D.A., Almondes K.G.D.S., Rogero M.M., Pollak D.F., Cozzolino S.M.F. (2022). Evaluation of biomarkers related to zinc nutritional status, antioxidant activity and oxidative stress in rheumatoid arthritis patients. Nutr. Health.

[133] Ala S., Shokrzadeh M., Pur Shoja A.M., Saeedi Saravi S.S. (2009). Zinc and copper plasma concentrations in Rheumatoid arthritis patients from a selected population in Iran. Pak. J. Biol. Sci..

[134] Hassan W.M. (2024). Oxidative DNA Damage and Zinc Status in Patients With Rheumatoid Arthritis in Duhok, Iraq. Cureus.

[135] Zoli A., Altomonte L., Caricchio R., Galossi A., Mirone L., Ruffini M.P., Magaró M. (1998). Serum zinc and copper in active rheumatoid arthritis: Correlation with interleukin 1β and tumour necrosis factor α. Clin. Rheumatol..

[136] Mierzecki A., Strecker D., Radomska K. (2011). A pilot study on zinc levels in patients with rheumatoid arthritis. Biol. Trace Elem. Res..

[137] Afridi H.I., Kazi T.G., Brabazon D., Naher S. (2012). Interaction between zinc, cadmium, and lead in scalp hair samples of pakistani and irish smokers rheumatoid arthritis subjects in relation to controls. Biol. Trace Elem. Res..

[138] Naveh Y., Schapira D., Ravel Y., Geller E., Scharf Y. (1997). Zinc metabolism in rheumatoid arthritis: Plasma and urinary zinc and relationship to disease activity. J. Rheumatol..

[139] Yang M., Su Y., Xu K., Wan X., Xie J., Liu L., Yang Z., Xu P. (2024). Iron, copper, zinc and magnesium on rheumatoid arthritis: A two-sample Mendelian randomization study. Int. J. Environ. Health Res..

[140] Fang D., Jiang D., Shi G., Song Y. (2024). The association between dietary zinc intake and osteopenia, osteoporosis in patients with rheumatoid arthritis. BMC Musculoskelet. Disord..

[141] Zhao Z., Ma X., Dong S., Yin H., Yang Y., Xiong G. (2023). Regulatory effect of zinc finger protein A20 on rheumatoid arthritis through NLRP3/ Caspase-1 signaling axis mediating pyroptosis of HFLS- RA cells. Cell. Mol. Biol..

[142] Hasan M., Yadav P., Ansari M.A., Ali S., Khan H.A. (2024). Therapeutic Dose of Zinc Aspartate and Zinc Citrate Attenuates Disease Activity Indices in Rheumatoid Arthritis. Biol. Trace Elem. Res..

[143] Simkin P.A. (1976). Oral Zinc Sulphate in Rheumatoid Arthritis. Lancet.

[144] Mattingly P.C., Mowat A.G. (1982). Zinc sulphate in rheumatoid arthritis. Ann. Rheum. Dis..

[145] Rasker J.J., Kardaun S.H. (1982). Lack of beneficial effect of zinc sulphate in rheumatoid arthritis. Scand. J. Rheumatol..

[146] Qi W., Jin L., Wu C., Liao H., Zhang M., Zhu Z., Han W., Chen Q., Ding C. (2023). Treatment with FAP-targeted zinc ferrite nanoparticles for rheumatoid arthritis by inducing endoplasmic reticulum stress and mitochondrial damage. Mater. Today Bio.

[147] Gad S.S., Fayez A.M., Abdelaziz M., Abou El-ezz D. (2021). Amelioration of autoimmunity and inflammation by zinc oxide nanoparticles in experimental rheumatoid arthritis. Naunyn Schmiedebergs Arch. Pharmacol..

[148] Ansari M.M., Ahmad A., Mishra R.K., Raza S.S., Khan R. (2019). Zinc Gluconate-Loaded Chitosan Nanoparticles Reduce Severity of Collagen-Induced Arthritis in Wistar Rats. ACS Biomater. Sci. Eng..

[149] Singh A., Boregowda S.S., Moin A., Abu Lila A.S., Aldawsari M.F., Khafagy E.S., Alotaibi H.F., Jayaramu R.A. (2022). Biosynthesis of Silver Nanoparticles Using Commiphora mukul Extract: Evaluation of Anti-Arthritic Activity in Adjuvant-Induced Arthritis Rat Model. Pharmaceutics.

[150] Zhang X., Fu X., Chen W., Chen P., Zhu H., Yang B., Liang J., Zeng F. (2025). Amelioration of the rheumatoid arthritis microenvironment using celastrol-loaded silver-modified ceria nanoparticles for enhanced treatment. J. Nanobiotechnol..

[151] He Z.H., Zou J.T., Chen X., Gong J.S., Chen Y., Jin L., Liu Y.W., Rao S.S., Yin H., Tan Y.J. (2023). Ångstrom-scale silver particles ameliorate collagen-induced and K/BxN-transfer arthritis in mice via the suppression of inflammation and osteoclastogenesis. Inflamm. Res..

[152] Disaanayake D.M.B.T., Faoagali J., Laroo H., Hancock G., Whitehouse M. (2014). Efficacy of some colloidal silver preparations and silver salts against Proteus bacteria, one possible cause of rheumatoid arthritis. Inflammopharmacology.

[153] Chen L., Sun Q., Peng S., Tan T., Mei G., Chen H., Zhao Y., Yao P., Tang Y. (2022). Associations of blood and urinary heavy metals with rheumatoid arthritis risk among adults in NHANES, 1999–2018. Chemosphere.

[154] Frangos T., Maret W. (2021). Zinc and cadmium in the aetiology and pathogenesis of osteoarthritis and rheumatoid arthritis. Nutrients.

[155] Murphy D., Sinha-Royle E., Bellis K., Harrington C., Hutchinson D. (2019). Nodular rheumatoid arthritis (RA): A distinct disease subtype, initiated by cadmium inhalation inducing pulmonary nodule formation and subsequent RA–associated autoantibody generation. Med. Hypotheses.

[156] Castañeda C.R., García-Martínez B., Zamudio-Cuevas Y., Fernández-Torres J., Flores K.M. (2025). Cadmium exposure and its role in joint disease: A brief review of experimental and population-based evidence. J. Trace Elem. Med. Biol..

[157] Liu H., Liu M., Qiao L., Yang Z., He Y., Bao M., Lin X., Han J. (2024). Association of blood cadmium levels and all-cause mortality among adults with rheumatoid arthritis: The NHANES cohort study. J. Trace Elem. Med. Biol..

[158] Bonaventura P., Courbon G., Lamboux A., Lavocat F., Marotte H., Albarède F., Miossec P. (2017). Protective effect of low dose intra-articular cadmium on inflammation and joint destruction in arthritis. Sci. Rep..

[159] Goldberg R.L., Kaplan S.R., Fuller G.C. (1983). Effect of heavy metals on human rheumatoid synovial cell proliferation and collagen synthesis. Biochem. Pharmacol..

[160] Motts J.A., Shirley D.L., Silbergeld E.K., Nyland J.F. (2014). Novel biomarkers of mercury-induced autoimmune dysfunction: A cross-sectional study in Amazonian Brazil. Environ. Res..

[161] Gardner R.M., Nyland J.F., Silva I.A., Maria Ventura A., Maria de Souza J., Silbergeld E.K. (2010). Mercury exposure, serum antinuclear/antinucleolar antibodies, and serum cytokine levels in mining populations in Amazonian Brazil: A cross-sectional study. Environ. Res..

[162] Wang C., Yang Y., Liang R., Wu S., Xuan C., Lv W., Li J. (2024). Preparation and anti-inflammatory effect of mercury sulphide nanoparticle-loaded hydrogels. J. Drug Target..

[163] Bamonti F., Fulgenzi A., Novembrino C., Ferrero M.E. (2011). Metal chelation therapy in rheumathoid arthritis: A case report: Successful management of rheumathoid arthritis by metal chelation therapy. BioMetals.

[164] Pedersen L.M., Christensen J.M. (1986). Chromium, Nickel and Cadmium in Biological Fluids in Patients with Rheumatoid Arthritis Compared to Healthy Controls. Acta Pharmacol. Toxicol..

[165] Afridi H.I., Kazi T.G., Talpur F.N., Naher S., Brabazon D. (2014). Relationship between toxic metals exposure via cigarette smoking and rheumatoid arthritis. Clin. Lab..

[166] Niedermeier W., Creitz E.E., Holley H.L. (1962). Trace metal composition of synovial fluid from patients with rheumatoid arthritis. Arthritis Rheum..

[167] Knekt P., Heliövaara M., Aho K., Alfthan G., Marniemi J., Aromaa A. (2000). Serum selenium, serum alpha-tocopherol, and the risk of rheumatoid arthritis. Epidemiology.

[168] Ma Y., Zhang X., Fan D., Xia Q., Wang M., Pan F. (2019). Common trace metals in rheumatoid arthritis: A systematic review and meta-analysis. J. Trace Elem. Med. Biol..

[169] Tarp U., Hansen J.C., Overvad K., Thorling E.B., Tarp B.D., Graudal H. (1987). Glutathione peroxidase activity in patients with rheumatoid arthritis and in normal subjects: Effects of long-term selenium supplementation. Arthritis Rheum..

[170] Qin J., Huang X., Wang N., Zhou P., Zhang H., Chen Z., Liang K., Gong D., Zeng Q., Niu P. (2021). Supranutritional selenium suppresses ROS-induced generation of RANKL-expressing osteoclastogenic CD4+ T cells and ameliorates rheumatoid arthritis. Clin. Transl. Immunol..

[171] Turk M.A., Liu Y., Pope J.E. (2023). Non-pharmacological interventions in the treatment of rheumatoid arthritis: A systematic review and meta-analysis. Autoimmun. Rev..

[172] Mehrpooya M., Majmasanaye M., Faramarzi F., Eshraghi A., Faress F. (2023). Investigation of the Effect of Oral Selenium on the Reduction of Clinical Symptoms and Joint Pain in Patients With Rheumatoid Arthritis in the Iranian Population. J. Clin. Pharmacol..

[173] Zamani B., Taghvaee F., Akbari H., Mohtashamian A., Sharifi N. (2024). Effects of Selenium Supplementation on the Indices of Disease Activity, Inflammation and Oxidative Stress in Patients with Rheumatoid Arthritis: A Randomized Clinical Trial. Biol. Trace Elem. Res..

[174] Uysal B., Sahin N., Kara H. (2024). Effects of Nutritional Status and Foods Consumed on Inflammation and Disease Activity in Patients with Rheumatoid Arthritis. Medicina.

[175] Peretz A., Siderova V., Neve J. (2001). Selenium supplementation in rheumatoid arthritis investigated in a double blind, placebo-controlled trial. Scand. J. Rheumatol..

[176] Stone J., Doube A., Dudson D., Wallace J. (1997). Inadequate calcium, folic acid, vitamin E, zinc, and selenium intake in rheumatoid arthritis patients: Results of a dietary survey. Semin. Arthritis Rheum..

[177] Rehman A., John P., Bhatti A. (2021). Biogenic selenium nanoparticles: Potential solution to oxidative stress mediated inflammation in rheumatoid arthritis and associated complications. Nanomaterials.

[178] Ren S.X., Zhang B., Lin Y., Ma D.S., Yan H. (2019). Selenium nanoparticles dispersed in phytochemical exert anti-inflammatory activity by modulating catalase, GPx1, and COX-2 gene expression in a rheumatoid arthritis rat model. Med. Sci. Monit..

[179] Zou B., Xiong Z., Yu Y., Shi S., Li X., Chen T. (2024). Rapid Selenoprotein Activation by Selenium Nanoparticles to Suppresses Osteoclastogenesis and Pathological Bone Loss. Adv. Mater..

[180] Shinde V., Desai K. (2024). Selenium-Methionine-Folic Acid Nanoparticles (SeMetFa NPs) and Its In Vivo Efficacy Against Rheumatoid Arthritis. Biol. Trace Elem. Res..

[181] Vieira A.T., Silveira K.D., Arruda M.C.C., Fagundes C.T., Gonçalves J.L., Silva T.A., Neves M.J., Menezes M.A.B.C., Nicoli J.R., Teixeira M.M. (2012). Treatment with Selemax®, a selenium-enriched yeast, ameliorates experimental arthritis in rats and mice. Br. J. Nutr..

[182] Goldhaber S.B. (2003). Trace element risk assessment: Essentiality vs. toxicity. Regul. Toxicol. Pharmacol..

[183] Hussain S.A., Abood S.J., Gorial F.I. (2017). The adjuvant use of calcium fructoborate and borax with etanercept in patients with rheumatoid arthritis: Pilot study. J. Intercult. Ethnopharmacol..

[184] Trivillin V.A., Abramson D.B., Bumaguin G.E., Bruno L.J., Garabalino M.A., Monti Hughes A., Heber E.M., Feldman S., Schwint A.E. (2014). Boron neutron capture synovectomy (BNCS) as a potential therapy for rheumatoid arthritis: Boron biodistribution study in a model of antigen-induced arthritis in rabbits. Radiat. Environ. Biophys..

[185] Prescha A., Zabłocka-Słowińska K.A., Płaczkowska S., Gorczyca D., Łuczak A., Grajeta H. (2019). Silicon intake and plasma level and their relationships with systemic redox and inflammatory markers in rheumatoid arthritis patients. Adv. Clin. Exp. Med..

[186] Boudigaard S.H., Schlünssen V., Vestergaard J.M., Søndergaard K., Torén K., Peters S., Kromhout H., Kolstad H.A. (2021). Occupational exposure to respirable crystalline silica and risk of autoimmune rheumatic diseases: A nationwide cohort study. Int. J. Epidemiol..

[187] Fireman E.M., Fireman Klein E. (2024). Association between silicosis and autoimmune disease. Curr. Opin. Allergy Clin. Immunol..

[188] Otsuki T., Maeda M., Murakami S., Hayashi H., Miura Y., Kusaka M., Nakano T., Fukuoka K., Kishimoto T., Hyodoh F. (2007). Immunological Effects of Silica and Asbestos. Cell. Mol. Immunol..

[189] Morotti A., Sollaku I., Franceschini F., Cavazzana I., Fredi M., Sala E., De Palma G. (2022). Systematic Review and Meta-analysis on the Association of Occupational Exposure to Free Crystalline Silica and Rheumatoid Arthritis. Clin. Rev. Allergy Immunol..

[190] Min Y.S., Kim M.G., Ahn Y.S. (2021). Rheumatoid arthritis in silica-exposed workers. Int. J. Environ. Res. Public Health.

[191] Speck-Hernandez C.A., Montoya-Ortiz G. (2012). Silicon, a Possible Link between Environmental Exposure and Autoimmune Diseases: The Case of Rheumatoid Arthritis. Arthritis.

[192] Ilar A., Klareskog L., Saevarsdottir S., Wiebert P., Askling J., Gustavsson P., Alfredsson L. (2019). Occupational exposure to asbestos and silica and risk of developing rheumatoid arthritis: Findings from a Swedish population-based case-control study. RMD Open.

[193] Blanc P.D., Trupin L., Yelin E.H., Schmajuk G. (2022). Assessment of Risk of Rheumatoid Arthritis Among Underground Hard Rock and Other Mining Industry Workers in Colorado, New Mexico, and Utah. JAMA Netw. Open.

[194] Cohen Tervaert J.W., Kappel R.M. (2013). Silicone implant incompatibility syndrome (SIIS): A frequent cause of ASIA (Shoenfeld’s syndrome). Immunol. Res..

[195] Fadil A., Muaidi Q.I., Alayat M.S., AlMatrafi N.A., Subahi M.S., Alshehri M.A. (2025). The Effectiveness of closed kinetic chain exercises in individuals with knee osteoarthritis: A systematic review and meta-analysis. PLoS ONE.

[196] Steinmetz J.D., Culbreth G.T., Haile L.M., Rafferty Q., Lo J., Fukutaki K.G., Cruz J.A., Smith A.E., Vollset S.E., Brooks P.M. (2023). Global, regional, and national burden of osteoarthritis, 1990-2020 and projections to 2050: A systematic analysis for the Global Burden of Disease Study 2021. Lancet Rheumatol..

[197] Mauri C., Cerulli C., Grazioli E., Minganti C., Tranchita E., Scotto di Palumbo A., Parisi A. (2025). Role of exercise on pain, functional capacity, and inflammatory biomarkers in osteoarthritis: A systematic review and meta-analysis. Ann. Phys. Rehabil. Med..

[198] Palazzo C., Nguyen C., Lefevre-Colau M.M., Rannou F., Poiraudeau S. (2016). Risk factors and burden of osteoarthritis. Ann. Phys. Rehabil. Med..

[199] Wei W., Qi X., Cheng B., He D., Qin X., Zhang N., Zhao Y., Chu X., Shi S., Cai Q. (2024). An atlas of causal association between micronutrients and osteoarthritis. Prev. Med..

[200] Carroll G.J., Breidahl W.H., Olynyk J.K. (2012). Characteristics of the arthropathy described in hereditary hemochromatosis. Arthritis Care Res..

[201] Xu J., Zhang S., Tian Y., Si H., Zeng Y., Wu Y., Liu Y., Li M., Sun K., Wu L. (2022). Genetic Causal Association between Iron Status and Osteoarthritis: A Two-Sample Mendelian Randomization. Nutrients.

[202] Radakovich L.B., Burton L.H., Culver L.A., Afzali M.F., Marolf A.J., Olver C.S., Santangelo K.S. (2022). Systemic iron reduction via an iron deficient diet decreases the severity of knee cartilage lesions in the Dunkin-Hartley guinea pig model of osteoarthritis. Osteoarthr. Cartil..

[203] Dixon S.J., Lemberg K.M., Lamprecht M.R., Skouta R., Zaitsev E.M., Gleason C.E., Patel D.N., Bauer A.J., Cantley A.M., Yang W.S. (2012). Ferroptosis: An iron-dependent form of nonapoptotic cell death. Cell.

[204] Zhang S., Xu J., Si H., Wu Y., Zhou S., Shen B. (2022). The Role Played by Ferroptosis in Osteoarthritis: Evidence Based on Iron Dyshomeostasis and Lipid Peroxidation. Antioxidants.

[205] Li H., Jiang X., Xiao Y., Zhang Y., Zhang W., Doherty M., Nestor J., Li C., Ye J., Sha T. (2023). Combining single-cell RNA sequencing and population-based studies reveals hand osteoarthritis-associated chondrocyte subpopulations and pathways. Bone Res..

[206] Pan Z., He Q., Zeng J., Li S., Li M., Chen B., Yang J., Xiao J., Zeng C., Luo H. (2022). Naringenin protects against iron overload-induced osteoarthritis by suppressing oxidative stress. Phytomedicine.

[207] Prasadam I., Schrobback K., Kranz-Rudolph B., Fischer N., Sonar Y., Sun A.R.J., Secondes E., Klein T., Crawford R., Subramaniam V.N. (2024). Effects of iron overload in human joint tissue explant cultures and animal models. J. Mol. Med..

[208] Camacho A., Simão M., Ea H.K., Cohen-Solal M., Richette P., Branco J., Cancela M.L. (2016). Iron overload in a murine model of hereditary hemochromatosis is associated with accelerated progression of osteoarthritis under mechanical stress. Osteoarthr. Cartil..

[209] Fang Z., Wang C., Zhu J., Gou Y. (2024). Iron overload promotes hemochromatosis-associated osteoarthritis via the mTORC1-p70S6K/4E-BP1 pathway. Int. Immunopharmacol..

[210] Jin Z., Zhang H., Bai L., Yue L., Zhang W., Liang J., Chang B., Yang Y., Hu Z., Chen L. (2023). Synovium is a sensitive tissue for mapping the negative effects of systemic iron overload in osteoarthritis: Identification and validation of two potential targets. J. Transl. Med..

[211] Xiang Z., Mei H., Wang H., Yao X., Rao J., Zhang W., Xu A., Lu L. (2025). Cuproptosis and its potential role in musculoskeletal disease. Front. Cell Dev. Biol..

[212] Wang Y., Sun Y., Jie T., Wang M., Zhang S., Yang H., Jian W., Dai D., Xu R., Yue B. (2025). Association between serum Copper-Zinc-Selenium mixture and multiple health outcomes. Bioact. Mater..

[213] Zhou J., Liu C., Sun Y., Francis M., Ryu M.S., Grider A., Ye K. (2021). Genetically predicted circulating levels of copper and zinc are associated with osteoarthritis but not with rheumatoid arthritis. Osteoarthr. Cartil..

[214] Zhou H., Zhang Y., Tian T., Wang B., Pan Y. (2024). Meta-analysis of the Relationship Between Zinc and Copper in Patients with Osteoarthritis. Biol. Trace Elem. Res..

[215] Luo H., Zhang Y., Meng C., Li C., Jia D., Xu Y. (2024). The effect of copper and vitamin D on osteoarthritis outcomes: A Mendelian randomization study. Medicine.

[216] Nam E., Han J., Suh J.M., Yi Y., Lim M.H. (2018). Link of impaired metal ion homeostasis to mitochondrial dysfunction in neurons. Curr. Opin. Chem. Biol..

[217] Brancaccio D., Gallo A., Piccioli M., Novellino E., Ciofi-Baffoni S., Banci L. (2017). [4Fe-4S] cluster assembly in mitochondria and its impairment by copper. J. Am. Chem. Soc..

[218] Urrutia P.J., Aguirre P., Tapia V., Carrasco C.M., Mena N.P., Núñez M.T. (2017). Cell death induced by mitochondrial complex I inhibition is mediated by Iron Regulatory Protein 1. Biochim. Biophys. Acta (BBA)-Mol. Basis Dis..

[219] Tandon M., Chetla N., Hodges J., Koul A., Dharia S., Shah D., Samayamanthula S., Raghuwanshi J.S., Sitsabeshon A., Oommen N. (2024). Mechanical Considerations and Clinical Implications of Joint Arthroplasty Metallosis. Cureus.

[220] Matusiewicz H., Richter M. (2022). Metal ions release from metallic orthopedic implants exposed to tribocorrosion and electrochemical corrosion conditions in simulated body fluids: Clinical context and in vitro experimental investigations. World J. Adv. Res. Rev..

[221] Dutta A., Nutt J., Slater G., Ahmed S. (2021). Review: Trunnionosis leading to modular femoral head dissociation. J. Orthop..

[222] Vendittoli P.A., Amzica T., Roy A.G., Lusignan D., Girard J., Lavigne M. (2011). Metal Ion Release With Large-Diameter Metal-on-Metal Hip Arthroplasty. J. Arthroplast..

[223] Stołtny T., Dobrakowski M., Augustyn A., Rokicka D., Kasperczyk S. (2023). The concentration of chromium and cobalt ions and parameters of oxidative stress in serum and their impact on clinical outcomes after metaphyseal hip arthroplasty with modular metal heads. J. Orthop. Surg. Res..

[224] Tower S.S., Cho C.S., Bridges R.L., Gessner B.D. (2021). Prevalence of Cobalturia among Adults with Joint Replacements. JAMA Netw. Open.

[225] Fu S., Meng H., Freer F., Kwon J., Shelton J.C., Knight M.M. (2020). Sub-toxic levels of Co^2+^ are anti-inflammatory and protect cartilage from degradation caused by IL-1β. Clin. Biomech..

[226] Díez-Tercero L., Delgado L.M., Bosch-Rué E., Perez R.A. (2021). Evaluation of the immunomodulatory effects of cobalt, copper and magnesium ions in a pro inflammatory environment. Sci. Rep..

[227] Moreno-Reyes R., Mathieu F., Boelaert M., Begaux F., Suetens C., Rivera M.T., Nève J., Perlmutter N., Vanderpas J. (2003). Selenium and iodine supplementation of rural Tibetan children affected by Kashin-Beck osteoarthropathy. Am. J. Clin. Nutr..

[228] Moreno-Reyes R., Suetens C., Mathieu F., Begaux F., Zhu D., Rivera M.T., Boelaert M., Nève J., Perlmutter N., Vanderpas J. (1998). Kashin-Beck osteoarthropathy in rural Tibet in relation to selenium and iodine status (abstract). N. Engl. J. Med..

[229] Ren F.L., Guo X., Zhang R.J., Wang S.J., Zuo H., Zhang Z.T., Geng D., Yu Y., Su M. (2007). Effects of selenium and iodine deficiency on bone, cartilage growth plate and chondrocyte differentiation in two generations of rats. Osteoarthr. Cartil..

[230] Zhang Y., Zhao X., Shan L., Liu M., Zhang Z., Wang Z., Zhang X., Meng H., Song Y., Zhang W. (2024). Chronic Iodine Intake Excess Damages the Structure of Articular Cartilage and Epiphyseal Growth Plate. Biol. Trace Elem. Res..

[231] Wang D., Zhang L., He Y., Wu J., Xia F., Li Q., Luo X. (2022). Identification for heavy metals exposure on osteoarthritis among aging people and Machine learning for prediction: A study based on NHANES 2011-2020. Front. Public Health.

[232] Farì G., Santagati D., Pignatelli G., Scacco V., Renna D., Cascarano G., Vendola F., Bianchi F.P., Fiore P., Ranieri M. (2021). Collagen Peptides, in Association with Vitamin C, Sodium Hyaluronate, Manganese and Copper, as Part of the Rehabilitation Project in the Treatment of Chronic Low Back Pain. Endocr. Metab. Immune Disord.-Drug Targets.

[233] Shi H., Wang H., Yu M., Su J., Zhao Z., Gao T., Zhang Q., Wei Y. (2024). Serum trace elements and osteoarthritis: A meta-analysis and Mendelian randomization study. J. Trace Elem. Med. Biol..

[234] Martínez-Nava G.A., Mendoza-Soto L., Fernández-Torres J., Zamudio-Cuevas Y., Reyes-Hinojosa D., Plata-Rodríguez R., Olivos-Meza A., Ruíz-Huerta E.A., Armienta-Hernández M.A., Hernández-Álvarez E. (2020). Effect of cadmium on the concentration of essential metals in a human chondrocyte micromass culture. J. Trace Elem. Med. Biol..

[235] Fernandez-Moreno M., Soto-Hermida A., Pertega S., Oreiro N., Fernandez-Lopez C., Rego-Perez I., Blanco F.J. (2011). Mitochondrial DNA (mtDNA) haplogroups and serum levels of anti-oxidant enzymes in patients with osteoarthritis. BMC Musculoskelet. Disord..

[236] Ostalowska A., Birkner E., Wiecha M., Kasperczyk S., Kasperczyk A., Kapolka D., Zon-Giebel A. (2006). Lipid peroxidation and antioxidant enzymes in synovial fluid of patients with primary and secondary osteoarthritis of the knee joint. Osteoarthr. Cartil..

[237] Zang Z.S., Xu Y.M., Lau A.T.Y. (2016). Molecular and pathophysiological aspects of metal ion uptake by the zinc transporter ZIP8 (SLC39A8). Toxicol. Res..

[238] Yang W.M., Lv J.F., Wang Y.Y., Xu Y.M., Lin J., Liu J., Chen J.J., Wang X.Z. (2023). The Daily Intake Levels of Copper, Selenium, and Zinc Are Associated with Osteoarthritis but Not with Rheumatoid Arthritis in a Cross-sectional Study. Biol. Trace Elem. Res..

[239] Zeng W., Hong E., Ye W., Ma L., Cun D., Huang F., Jiang Z. (2025). Mendelian randomization of serum micronutrients and osteoarthritis risk: Focus on zinc. Nutr. J..

[240] Qu X., Yang H., Yu Z., Jia B., Qiao H., Zheng Y., Dai K. (2020). Serum zinc levels and multiple health outcomes: Implications for zinc-based biomaterials. Bioact. Mater..

[241] Jakoniuk M., Biegaj M., Kochanowicz J., Łysoń T., Lankau A., Wilkiel M., Socha K. (2022). Relationship between Selected Micronutrient Concentrations, Total Antioxidant Status, Pain Severity, and the Image of 1H MR Spectroscopy in Degenerative Spine Disease: A Case-Control Study. J. Clin. Med..

[242] Choi W.S., Chun J.S. (2017). Upregulation of lipocalin-2 (LCN2) in osteoarthritic cartilage is not necessary for cartilage destruction in mice. Osteoarthr. Cartil..

[243] Lee M., Won Y., Shin Y., Kim J.H., Chun J.S. (2016). Reciprocal activation of hypoxia-inducible factor (HIF)-2α and the zinc-ZIP8-MTF1 axis amplifies catabolic signaling in osteoarthritis. Osteoarthr. Cartil..

[244] Li L., Cao J., Li L., Wu G., Xiao J. (2024). Associations of Blood Cadmium Levels With Osteoarthritis Among US Adults in NHANES 2013-2018. J. Occup. Environ. Med..

[245] Jakoniuk M., Kochanowicz J., Lankau A., Wilkiel M., Socha K. (2022). Concentration of Selected Macronutrients and Toxic Elements in the Blood in Relation to Pain Severity and Hydrogen Magnetic Resonance Spectroscopy in People with Osteoarthritis of the Spine. Int. J. Environ. Res. Public Health.

[246] Eduviges Z.C.Y., Martínez-Nava G., Reyes-Hinojosa D., Mendoza-Soto L., Fernández-Torres J., López-Reyes A., Olivos-Meza A., Armienta-Hernández M.A., Ruíz-Huerta E.A., de Jesús González-Guadarrama M. (2020). Impact of cadmium toxicity on cartilage loss in a 3D in vitro model. Environ. Toxicol. Pharmacol..

[247] Bodo M., Balloni S., Lumare E., Bacci M., Calvitti M., Dell’Omo M., Murgia N., Marinucci L. (2010). Effects of sub-toxic Cadmium concentrations on bone gene expression program: Results of an in vitro study. Toxicol. In Vitro.

[248] Chen X., Wang G., Li X., Gan C., Zhu G., Jin T., Wang Z. (2013). Environmental level of cadmium exposure stimulates osteoclasts formation in male rats. Food Chem. Toxicol..

[249] Urzì Brancati V., Aliquò F., Freni J., Pantano A., Galipò E., Puzzolo D., Minutoli L., Marini H.R., Campo G.M., D’Ascola A. (2024). The Effects of Seleno-Methionine in Cadmium-Challenged Human Primary Chondrocytes. Pharmaceuticals.

[250] Marsit C.J. (2015). Influence of environmental exposure on human epigenetic regulation. J. Exp. Biol..

[251] Chauhan S., Dunlap K., Duffy L.K. (2019). Effects of methylmercury and theaflavin digallate on adipokines in mature 3T3-L1 adipocytes. Int. J. Mol. Sci..

[252] Zioła-Frankowska A., Dąbrowski M., Kubaszewski Ł., Rogala P., Kowalski A., Frankowski M. (2017). An analysis of factors affecting the mercury content in the human femoral bone. Environ. Sci. Pollut. Res..

[253] Kosik-Bogacka D.I., Lanocha-Arendarczyk N., Kot K., Ciosek Z., Zietek P., Karaczun M., Pilarczyk B., Tomza-Marciniak A., Podlasinska J., Kalisinska E. (2017). Effects of biological factors and health condition on mercury and selenium concentrations in the cartilage, meniscus and anterior cruciate ligament. J. Trace Elem. Med. Biol..

[254] DeMartini J., Wilson A., Powell J.S., Powell C.S. (2001). Lead Arthropathy and Systemic Lead Poisoning from an Intraarticular Bullet. Am. J. Roentgenol..

[255] Nelson A.E., Shi X.A., Schwartz T.A., Chen J.C., Renner J.B., Caldwell K.L., Helmick C.G., Jordan J.M. (2011). Whole blood lead levels are associated with radiographic and symptomatic knee osteoarthritis: A cross-sectional analysis in the Johnston County Osteoarthritis Project. Arthritis Res. Ther..

[256] Khaled Abo-Elmaaty R. (2017). The correlation between blood lead levels and knee osteoarthritis: A preliminary Egyptian study. Int. J. Clin. Rheumatol..

[257] Carmouche J.J., Puzas J.E., Zhang X., Tiyapatanaputi P., Cory-Slechta D.A., Gelein R., Zuscik M., Rosier R.N., Boyce B.F., O’Keefe R.J. (2005). Lead exposure inhibits fracture healing and is associated with increased chondrogenesis, delay in cartilage mineralization, and a decrease in osteoprogenitor frequency. Environ. Health Perspect..

[258] Holz J.D., Beier E., Sheu T.J., Ubayawardena R., Wang M., Sampson E.R., Rosier R.N., Zuscik M., Puzas J.E. (2012). Lead induces an osteoarthritis-like phenotype in articular chondrocytes through disruption of TGF-β signaling. J. Orthop. Res..

[259] Vaziri N.D., Khan M. (2007). Interplay of reactive oxygen species and nitric oxide in the pathogenesis of experimental lead-induced hypertension. Clin. Exp. Pharmacol. Physiol..

[260] Yan R., Ding J., Yang Q., Zhang X., Han J., Jin T., Shi S., Wang X., Zheng Y., Li H. (2023). Lead acetate induces cartilage defects and bone loss in zebrafish embryos by disrupting the GH/IGF-1 axis. Ecotoxicol. Environ. Saf..

[261] Zoeger N., Roschger P., Hofstaetter J.G., Jokubonis C., Pepponi G., Falkenberg G., Fratzl P., Berzlanovich A., Osterode W., Streli C. (2006). Lead accumulation in tidemark of articular cartilage. Osteoarthr. Cartil..

[262] Dahlstrand H., Stark A., Anissian L., Hailer N.P. (2009). Elevated Serum Concentrations of Cobalt, Chromium, Nickel, and Manganese After Metal-On-Metal Alloarthroplasty of the Hip: A Prospective Randomized Study. J. Arthroplast..

[263] Cracchiolo A., Revell P. (1982). Metal Concentration in Synovial Fluids of Patients with Prosthetic Knee Arthroplasty. Clin. Orthop. Relat. Res..

[264] Savarino L., Cadossi M., Chiarello E., Fotia C., Greco M., Baldini N., Giannini S. (2014). How do metal ion levels change over time in hip resurfacing patients? A cohort study. Sci. World J..

[265] Brodziak-Dopierała B., Kwapuliński J., Sobczyk K., Kowol J. (2011). The occurrence of nickel and other elements in tissues of the hip joint. Ecotoxicol. Environ. Saf..

[266] Navratilova P., Vejvodova M., Vaculovic T., Slaninova I., Emmer J., Tomas T., Ryba L., Burda J., Pavkova Goldbergova M. (2024). Cytotoxic effects and comparative analysis of Ni ion uptake by osteoarthritic and physiological osteoblasts. Sci. Rep..

[267] Arumugam M., Jeyaraman N., Ramasubramanian S., Hari R., Jeyaraman M. (2024). Predictive Modeling of Osteoarthritis Using Biochemical Markers: A Cross-Sectional Analysis. J. Orthop. Case Rep..

[268] Wahl L., Samson Chillon T., Seemann P., Ohrndorf S., Ochwadt R., Becker W., Schomburg L., Hoff P. (2025). Serum selenium, selenoprotein P and glutathione peroxidase 3 in rheumatoid, psoriatic, juvenile idiopathic arthritis, and osteoarthritis. J. Nutr. Biochem..

[269] Ba Y., Sun L., Zuo J., Yu S.Y., Yang S., Ding L.M., Feng Z.C., Li Z.Y., Zhou G.Y., Yu F.F. (2022). Association of oxidative stress and Kashin–Beck disease integrated Meta and Bioinformatics analysis. Osteoarthr. Cartil..

[270] Cao J.J., Gregoire B.R., Zeng H. (2012). Selenium deficiency decreases antioxidative capacity and is detrimental to bone microarchitecture in mice. J. Nutr..

[271] Mcclung J.P., Roneker C.A., Mu W., Lisk D.J., Langlais P., Liu F., Lei X.G. (2004). Development of insulin resistance and obesity in mice overexpressing cellular glutathione peroxidase. Proc. Natl. Acad. Sci. USA.

[272] Masuko K., Schomburg L., Aleemuddin Quamri M., Tan Y. (2023). A national cross-sectional analysis of selenium intake and risk of osteoarthritis: NHANES 2003–2016. Front. Public Health.

[273] Pizzorno L. (2015). Nothing Boring About Boron. Integr. Med..

[274] Nielsen F.H., Eckhert C.D. (2020). Boron. Adv Nutr..

[275] Gaby A.R. (1999). Natural treatments for osteoarthritis. Altern. Med. Rev..

[276] Helliwell T.R., Kelly S.A., Walsh H.P.J., Klenerman L., Haines J., Clark R., Roberts N.B. (1996). Elemental Analysis of Femoral Bone From Patients With Fractured Neck of Femur or Osteoarthrosis. Bone.

[277] Peng X.U., Lingxia Z., Schrauzer G.N., Xiong G. (2000). Selenium, Boron, and Germanium Deficiency in the Etiology of Kashin-Beck Disease. Biol. Trace Elem. Res..

[278] Fang W., Wu P., Hu R., Huang Z. (2003). Environmental Se-Mo-B Deficiency and its possible effects on crops and Keshan-Beck disease (KBD) in the Chousang area, Yao County, Shaanxi Province, China. Environ. Geochem. Health.

[279] Karmacharya P., Chakradhar R., Ogdie A. (2021). The epidemiology of psoriatic arthritis: A literature review. Best Pract. Res. Clin. Rheumatol..

[280] Kishimoto M., Deshpande G.A., Fukuoka K., Kawakami T., Ikegaya N., Kawashima S., Komagata Y., Kaname S. (2021). Clinical features of psoriatic arthritis. Best Pract. Res. Clin. Rheumatol..

[281] Azuaga A.B., Ramírez J., Cañete J.D. (2023). Psoriatic Arthritis: Pathogenesis and Targeted Therapies. Int. J. Mol. Sci..

[282] Oriente P., Scarpa R., Pucino A., Torella M., Riccio A., Oriente C.B. (1984). Supportive laboratory findings in psoriatic arthritis. Clin. Rheumatol..

[283] Gröber U., Tsiami S., Chillon T.S., Rousis E., Kisters K., Karmeli S., Kiltz U., Schomburg L., Baraliakos X. (2025). Trace Element Deficiency in Axial Spondyloarthritis and Psoriatic Arthritis in Relation to Markers of Inflammation and Remission. Int. J. Mol. Sci..

[284] Gao Y., Li X., Liu T., Liu Z. (2021). The Effect of Methotrexate on Serum Levels of Trace/Mineral Elements in Patients with Psoriatic Arthritis. Biol. Trace Elem. Res..

[285] (1995). Fatty acids and antioxidant micronutrients in psoriatic arthritis. J. Reumathol..

[286] Yen J.H., Tsai W.C., Lin C.H., Ou T.T., Hu C.J., Liu H.W. (2004). Manganese Superoxide Dismutase Gene Polymorphisms in Psoriatic Arthritis. Dis. Markers.

[287] Clemmensen O.J., Siggaard-Andersen J., Worm A.M., Stahl D., Frost F., Bloch I. (1980). Psoriatic arthritis treated with oral zinc sulphate. Br. J. Dermatol..

[288] Leibovici V., Statter M., Weinrauch L., Tzfoni E., Matzner Y. (1990). Effect of zinc therapy on neutrophil chemotaxis in psoriasis. Isr. J. Med. Sci..

[289] Markiewicz-Górka I., Chowaniec M., Martynowicz H., Wojakowska A., Jaremków A., Mazur G., Wiland P., Pawlas K., Poręba R., Gać P. (2022). Cadmium Body Burden and Inflammatory Arthritis: A Pilot Study in Patients from Lower Silesia, Poland. Int. J. Environ. Res. Public Health.

[290] Deyab G., Hokstad I., Aaseth J., Småstuen M.C., Whist J.E., Agewall S., Lyberg T., Tveiten D., Hjeltnes G., Zibara K. (2018). Effect of anti-rheumatic treatment on selenium levels in inflammatory arthritis. J. Trace Elem. Med. Biol..

[291] Wei Y., Zhang S., Shao F., Sun Y. (2025). Ankylosing spondylitis: From pathogenesis to therapy. Int. Immunopharmacol..

[292] Givian A., Azizan A., Jamshidi A., Mahmoudi M., Farhadi E. (2025). Iron metabolism in rheumatic diseases. J. Transl. Autoimmun..

[293] Chang S., Tang M., Zhang B., Xiang D., Li F. (2022). Ferroptosis in inflammatory arthritis: A promising future. Front. Immunol..

[294] Wang X., Qiu L., Yang Z., Wu C., Xie W., Zhang J., Li W., Li W., Gao Y., Zhang T. (2024). Association between serum iron status and the risk of five bone and joint-related diseases: A Mendelian randomization analysis. Front. Endocrinol..

[295] Yang M., Yu H., Xu K., Xie J., Zheng H., Feng R., Wang J., Xu P. (2023). No evidence of a genetic causal relationship between ankylosing spondylitis and iron homeostasis: A two-sample Mendelian randomization study. Front. Nutr..

[296] Zhang P., Chen H., Zhang Y., Liu Y., Zhu G., Zhao W., Shang Q., He J., Zhou Z., Shen G. (2024). Dry and wet experiments reveal diagnostic clustering and immune landscapes of cuproptosis patterns in patients with ankylosing spondylitis. Int. Immunopharmacol..

[297] Wei B., Wang S., Li S., Gu Q., Yue Q., Tang Z., Zhang J., Liu W. (2025). Unveiling Cuproptosis-Driven Molecular Clusters and Immune Dysregulation in Ankylosing Spondylitis. J. Inflamm. Res..

[298] Sun X., Deng Y., Ma Y., Shao M., Ni M., Zhang T., Wang X., Xu S., Chen Y., Xu S. (2022). Common mineral nutrients in ankylosing spondylitis: A 2-sample Mendelian randomization study. Int. J. Rheum. Dis..

[299] Yen J.H., Tsai W.C., Chen C.J., Lin C.H., Ou T.T., Hu C.J., Liu H.W. (2003). Cytochrome P450 1A1 and manganese superoxide dismutase genes polymorphisms in ankylosing spondylitis. Immunol. Lett..

[300] Shiue I. (2015). Relationship of environmental exposures and ankylosing spondylitis and spinal mobility: US NHAENS, 2009-2010. Int. J. Environ. Health Res..

[301] Hulander E., Zverkova Sandström T., Beckman Rehnman J., Law L., Söderberg S., Forsblad-d’Elia H. (2023). Patients with radiographic axial spondylarthritis have an impaired dietary intake—A cross-sectional study with matched controls from northern Sweden. Arthritis Res. Ther..

[302] Santacruz J.C., Mantilla M.J., Pulido S., Isaza J.R., Tuta E., Agudelo C.A., Londono J. (2023). A Practical Overview of the Articular Manifestations of Systemic Lupus Erythematosus. Cureus.

[303] Ceccarelli F., Perricone C., Cipriano E., Massaro L., Natalucci F., Capalbo G., Leccese I., Bogdanos D., Spinelli F.R., Alessandri C. (2017). Joint involvement in systemic lupus erythematosus: From pathogenesis to clinical assessment. Semin. Arthritis Rheum..

[304] Carini M., Fredi M., Cavazzana I., Bresciani R., Ferrari F., Monti E., Franceschini F., Biasiotto G. (2023). Frequency Evaluation of the Interleukin-6 −174G>C Polymorphism and Homeostatic Iron Regulator (HFE) Mutations as Disease Modifiers in Patients Affected by Systemic Lupus Erythematosus and Rheumatoid Arthritis. Int. J. Mol. Sci..

[305] Wincup C., Sawford N., Rahman A. (2021). Pathological mechanisms of abnormal iron metabolism and mitochondrial dysfunction in systemic lupus erythematosus. Expert Rev. Clin. Immunol..

[306] Dong W., Xu H., Wei W., Ning R., Chang Y. (2024). Advances in the study of ferroptosis and its relationship to autoimmune diseases. Int. Immunopharmacol..

[307] Zheng Y., Yan F., He S., Luo L. (2024). Targeting ferroptosis in autoimmune diseases: Mechanisms and therapeutic prospects. Autoimmun. Rev..

[308] Lai B., Wu C.H., Wu C.Y., Luo S.F., Lai J.H. (2022). Ferroptosis and Autoimmune Diseases. Front. Immunol..

[309] Liu T., Huang Y., Wang Y., Shen H. (2025). Disrupting the immune homeostasis: The emerging role of macrophage ferroptosis in autoimmune diseases. Int. Immunopharmacol..

[310] Chang J., Wu Q., Wang G. (2025). Research advancements in the association between prevalent trace metals and connective tissue diseases. Environ. Geochem. Health.

[311] Tóth C.N., Baranyai E., Csípő I., Tarr T., Zeher M., Posta J., Fábián I. (2017). Elemental Analysis of Whole and Protein Separated Blood Serum of Patients with Systemic Lupus Erythematosus and Sjögren’s Syndrome. Biol. Trace Elem. Res..

[312] Stejskal V., Reynolds T., Bjørklund G. (2015). Increased frequency of delayed type hypersensitivity to metals in patients with connective tissue disease. J. Trace Elem. Med. Biol..

[313] Bjørklund G., Dadar M., Aaseth J. (2018). Delayed-type hypersensitivity to metals in connective tissue diseases and fibromyalgia. Environ. Res..

[314] Ye D., Sun X., Guo Y., Shao K., Qian Y., Huang H., Liu B., Wen C., Mao Y. (2021). Genetically determined selenium concentrations and risk for autoimmune diseases. Nutrition.

